# Ancient DNA unravels the history of chickens in the Baltic Sea region and the continuity of landrace lineages

**DOI:** 10.1038/s41437-026-00842-9

**Published:** 2026-04-13

**Authors:** Suvi Olli, Rudolf Gustavsson, Hanna Kivikero, Lembi Lõugas, Kristiina Mannermaa, Giedrė Piličiauskienė, Eve Rannamäe, Jeremy B. Searle, Laura Kvist, Johanna Honka

**Affiliations:** 1 Ecology and Genetics Research Unit, University of Oulu, Oulu, Finland; 2 Stiftelsen kulturmiljövård, Pilgatan 8D, Västerås, Sweden; 3 Department of Philosophy, History, and Art Studies, University of Helsinki, Helsinki, Finland; 4 Archaeological Research Collection, Tallinn University, Tallinn, Estonia; Department of Zoology, Institute of Ecology and Earth Sciences, University of Tartu, Tartu, Estonia; 5 Department of Cultures, archaeology, University of Helsinki, Helsinki, Finland; 6 Faculty of History, Department of Archaeology, Vilnius University, Vilnius, Lithuania; 7 Department of Archaeology, Institute of History and Archaeology, University of Tartu, Tartu, Estonia; 8 Department of Ecology and Evolutionary Biology, Cornell University, Ithaca, NY USA

**Keywords:** Animal breeding, DNA sequencing

## Abstract

Very little is known about the origins and history of domestic chickens (*Gallus gallus domesticus*) in northern Europe due to a lack of existing documentary and ancient DNA evidence from this region. Therefore, we conducted ancient DNA analyses and radiocarbon dating of archaeological chicken bones from the Baltic Sea region (Finland, Estonia, and Lithuania). We sequenced a 201-bp long fragment of the mitochondrial control region as well as SNPs (single nucleotide polymorphisms) from the *thyroid-stimulating hormone receptor* (*TSHR*) gene and the *β-carotene dioxygenase 2* (*BCDO2*) gene, comparing with modern Finnish and Estonian landrace chickens, as well as with other ancient and modern chickens. All studied ancient chickens belonged to a prevalent E1 mitochondrial haplogroup, except one individual from the Åland Islands (haplogroup B). Allele frequencies differed between ancient Baltic and Finnish chickens from Åland Islands in *TSHR* and *BCDO2* genes, with Åland harbouring more individuals with grey skin. Interestingly, yellow-skinned chickens were more common in mainland Finland and Baltic countries during ancient times than in central and southern Europe. Mitochondrial haplogroup A was present in modern Finnish landrace chickens but not in ancient samples from the early Finnish Iron Age to the early modern period (3^rd^–18^th^ century CE), indicating later introgression. Both Estonian and Finnish landrace chickens had a higher frequency of the *TSHR* wild-type allele than the modern reference samples. Based on our results, the ancient chickens from the Åland Islands differed from other ancient chickens from the Baltic Sea region, and the landrace chickens differ from other modern chickens.

## Introduction

Although chicken meat and eggs are among the most consumed animal products globally, with approximately 140 million tonnes of meat and 1.7 billion tonnes of eggs produced annually, with a rising trend (FAO 2025), many aspects of the domestication history of the species remain unknown. For example, the origin, domestication centre(s), and domesticated subspecies of the chicken have been debated over the years, with controversies arising from excavation and recovery biases of small bones, difficulties in identifying galliform species from each other, and lack of direct radiocarbon dating of specimens (for review see Peters et al. 2024). Modern genomic evidence suggests that the chicken was domesticated from the red junglefowl (*Gallus gallus*), likely from the subspecies *G. gallus spadiceus* (Wang et al. 2020). After domestication, chickens were transported across Southeast and South Asia, where they admixed with other red junglefowl subspecies and *Gallus* species (Wang et al. 2020). Mohenjo-Daro, an archaeological site in the Indus Valley around 2000 BCE (Before Common Era), provided the earliest evidence of domestic chickens (Zeuner 1963). However, upon re-examination, the species identification of the bones was questionable, and they could have been later intrusions from upper layers (Peters et al. 2022; West and Zhou 1989). To date, the earliest unambiguous chickens have been recovered from the site of Ban Non Wat in central Thailand, dated to 1650–1250 BCE (Peters et al. 2022), though southwestern China could harbour earlier chicken remains from 2480–2210 BCE (Peng et al. 2022). There have been claims for early chicken domestication in China (6000–8000 BCE; West and Zhou 1989; Xiang et al. 2014), but these claims have been refuted as belonging to *Phasianidae* bones or implausible due to climate modelling, which indicated that the past climate in the area was not suitable for the red junglefowl (Barton et al. 2020; Deng et al. 2014; Eda et al. 2016; Peng et al. 2015; Peters et al. 2015; Peters et al. 2016).

Dispersal post-domestication has been one of the most studied questions. Mitochondrial DNA from modern and ancient samples has been used to study the similarity of chickens worldwide and to draw conclusions about possible dispersal routes (Miao et al. 2013; Storey et al. 2007, 2010, 2012, 2013; Thomson et al. 2014). Initially ancient DNA (aDNA) was used as evidence of pre-Columbian introduction of chickens to Central and South America (Storey et al. 2007, 2012), but later studies showed that this result was probably due to contaminated reagents (Gongora et al. 2008; Thomson et al. 2014), as modern chicken DNA is known to be present in laboratory chemicals (Leonard et al. 2007). Most aDNA studies of chickens have focused on the dispersal of chickens from Asia to Oceania and Central and South America (Storey et al. 2007, 2010, 2012, 2013; Thomson et al. 2014), but the history of domestic chickens has also been studied in Europe (Girdland Flink et al. 2014; Storey et al. 2012). Studies of mitochondrial aDNA suggest that one haplogroup, named E1, has been dominant for a large part of the breeding history in Europe and western Russia., as all studied central and southern European chickens from around the 3^rd^ century BCE to the 18^th^ century CE (Common Era) and western Russian chickens from the 9^th^ to the 18^th^ century CE belong to this haplogroup (Dyomin et al. 2017; Girdland Flink et al. 2014; Lebrasseur et al. 2021), except one western Russian sample from the 18^th^ century CE that has been assigned to haplogroup C1 (Dyomin et al. 2017). The E1 haplogroup continues to be dominant in most parts of the world (Miao et al. 2013).

There are no aDNA studies of chickens from northern Europe, and knowledge of ancient chickens from this region is very limited since they are seldom mentioned in written sources. This may partly be due to the smaller economic value of chickens in the past compared to, for example, cattle and horses. Hence, chickens were rarely included in records (Bläuer 2019; Walker and Meijer 2020). The earliest chicken findings from Europe are from Italy and have been dated to 780 – 540 cal BCE, indicating that chickens were introduced to Europe during the 1^st^ millennium BCE (Best et al. 2022). Chicken findings from France and Britain suggest that by the 5^th^ and 6^th^ century CE, they had been introduced further north (Best et al. 2022). However, it has been suggested that the introduction of chickens to northern parts of Europe may have taken over 1000 years from the initial introduction to southern Europe (Best et al. 2022). In the Baltic Sea region, the earliest chicken findings are dated to: 685–880 cal CE in Finland (Ukkonen and Mannermaa 2017; Wessman et al. 2018), 200 cal BCE–5 cal CE in Estonia (Ehrlich et al. 2021), 200 BCE–200 CE in Lithuania (Vėlius et al. 2025), 400 BCE–550 CE and 1–100 CE in Sweden (Ericson and Tyrberg 2004; Lepiksaar 1977; reviewed in Tyrberg 2002), 800 CE and 887–986 cal CE in Norway (Barrett et al. 2007; Walker et al. 2025), and 202 cal BCE–21 cal CE in Denmark (Gotfredsen 2013; Peters et al. 2022; Raahauge 2002), but only Finnish, Estonian and Danish samples have been radiocarbon dated.

In addition to dispersal, selection, especially human-driven artificial selection, has been studied in chickens (Elferink et al. 2012; Rubin et al. 2010). A whole-genome resequencing study found that several loci show selective sweeps in modern chickens (Rubin et al. 2010). One of these identified sweep loci was the *TSHR* locus, which codes for the thyroid-stimulating hormone receptor. A non-conservative glycine to arginine amino acid substitution at residue 558 (chr5:40,089,599 G/A) was identified as the causal mutation of the selective sweep. This SNP (single nucleotide polymorphism) is possibly linked to shifts in seasonal mating, causing earlier egg laying at sexual maturity, as well as reduced aggressiveness towards other chickens and reduced fear of humans, which are common features in domesticated animals (Karlsson et al. 2015, 2016; Rubin et al. 2010). The function of this SNP has not been experimentally validated in chicken, but CRISPR-Cas genome-edited house mice (*Mus musculus*) made homozygous for the respective arginine had significant physiological and metabolic differences compared to wild-type individuals (Wang et al. 2021). Hence, the *TSHR*-Gly558Arg mutation could be biologically functional (Wang et al. 2021). This locus was suggested to have played a crucial role in the domestication history of chickens, as essentially all modern chickens carry the sweep allele (Rubin et al. 2010). However, based on aDNA studies, this locus has been under selection rather recently, beginning 1100 years ago, and the sweep allele has been fixed during the last 500 years (Girdland Flink et al. 2014; Loog et al. 2017; Lyu et al. 2022). Interestingly, the timing of selection for this locus correlates with the change of husbandry practices in Europe due to Christian fasting practices, which restricted meat consumption from four-legged animals and increased the consumption of chicken meat (Loog et al. 2017). Also, other factors, like urbanisation and population growth, led to an increase in chicken consumption as people kept small flocks of chickens to provide eggs and meat for households (Loog et al. 2017; Lyu et al. 2022).

Another locus suggested to be under selection during the breeding history of the chicken is the *BCDO2* (*β-carotene dioxygenase 2*) gene, which codes for an enzyme that cleaves colourful carotenoids into colourless apocarotenoids (Eriksson et al. 2008; Kiefer et al. 2001; Rubin et al. 2010). A recessive mutation in this locus has been found to cause a yellow skin phenotype in chickens, provided that the diet has enough carotenoids (Eriksson et al. 2008). Individuals with a dominant, wild-type allele of this gene have a white or grey skin colour, while most modern chicken breeds have yellow skin (Eriksson et al. 2008). Genetic evidence indicates that domestic chickens acquired the yellow skin allele from the grey junglefowl (*Gallus sonneratii*) through admixture after the initial domestication from the red junglefowl (Eriksson et al. 2008). Based on aDNA, the yellow skin colour-causing allele was acquired before chickens were brought to Europe, but none of the central and southern European chickens from the 3^rd^ century BCE to the 18^th^ century CE were found to be homozygous for the recessive yellow skin allele; thus, none had yellow skin, and only a few were heterozygous (Girdland Flink et al. 2014). The yellow skin allele has been driven to a higher frequency during recent breed formations (Girdland Flink et al. 2014; Loog et al. 2017), probably due to aesthetic factors, and since yellow skin colour was viewed as an indicator of health and higher nutrient content, for example, it has been suggested that yellow-skinned chickens were less likely to have avian diseases such as coccidiosis (Ndenga et al. 2017; Sunde 1992).

Most modern chickens currently raised in Europe belong to commercial breeds that were founded after the Industrial Revolution of the 18^th^ and 19^th^ centuries. Since their founding, these breeds have been developed through intensive selection and crossbreeding (Bell 2002). Therefore, their genetic make-up differs from that of the ancient chicken populations once present in Europe. However, in several countries around the Baltic Sea, some lineages of native landrace chickens are believed to have persisted, for example, in Finland and Estonia (Eesti Maakana Selts 2025; NordGen 2025). Landrace chickens are locally adapted chicken breeds or populations that have been raised in the area for a long time, and crosses with commercial production breeds are avoided; thus, they may descend from ancient chickens. Landrace chickens in Finland and Estonia have been rescued from near extinction based on a small number of individuals per lineage. The low numbers of remaining landrace individuals reflect the lower productivity of landraces compared with highly improved commercial breeds. The Finnish landrace chicken (suomalainen maatiaiskana) is an official breed with ten family lines (Piikkiö, Alho, Hornio, Savitaipale, Ilmajoki, Kiuruvesi, Häme, Iitti, Tyrnävä, and Jussila), named after the town or region where they were found (NordGen 2025). An additional two populations exist (Lindell and Luumäki) but are not included in the conservation program due to undocumented origins (NordGen 2025). The Finnish landrace chicken is a light egg layer and a multipurpose breed of small to medium size with variable plumage colours (NordGen 2025). In the 1970s, the remaining individuals from different family lines originating from remote villages were saved from extinction, and in 1998, a conservation program was initiated (NordGen 2025). The Estonian landrace chicken (eesti maakana) is not acknowledged as a breed, but in the late 1920s and 1930s, several family lines were established, and conservation work began. Estonian landrace chickens are multi-coloured and often have a tuft on the back of the head, and they are divided into two types: small and large chickens (Eesti Maakana Selts 2025). Currently, seven lineages (Vormsi, Keedika, Lõo, Liivi, Viru, Võru, and Härmä) and their mixes exist (Eesti Maakana Selts 2025). No landrace chicken populations are known from Lithuania.

Landrace breeds are well-adapted to the environments they have been raised in for a long time, and they often have more genetic diversity and better disease resistance than commercial breeds (Fulton et al. 2017; Malomane et al. 2019; Muir et al. 2008). For example, the Finnish landrace chickens are better adapted to cold, can forage by themselves during summer, and have maintained the tendency to brood and take care of offspring. Finnish landrace chickens have maintained high levels of genetic diversity as well as disease resistance, even though the lineages have been rescued from a small number of individuals (Berres et al. 2020; Fulton et al. 2017). In addition, the Finnish landrace chicken lineages are genetically distinct (Berres et al. 2020; Fulton et al. 2017). Given their higher genetic diversity and greater resistance to diseases, landrace chickens could represent a valuable genetic resource for the development of modern production breeds exhibiting low levels of genetic diversity, particularly as environmental conditions change and new diseases emerge.

In this study, we aimed to assess the genetic similarity between ancient chickens from the Baltic Sea region and those previously studied from central and southern Europe and western Russia. For this purpose, we (1) extracted aDNA from archaeological bones of Finnish, Estonian, and Lithuanian chickens and analysed a 201-bp fragment of mitochondrial DNA to study maternal evolutionary lineages. (2) We analysed allele frequencies for nuclear SNPs of the *TSHR* and *BCDO2* genes and compared the results with previously studied central and southern European ancient chickens. (3) We radiocarbon dated selected ancient chicken samples to establish a chronology of chicken finds. (4) We aimed to evaluate the similarity of the modern landrace chickens from Finland and Estonia with the ancient chickens from these regions. As genetic similarity may indicate continuity of ancient chicken lineages to modern landrace lineages, we compared the mitochondrial sequences and the same SNPs of modern landrace chickens to the ancient ones from the same geographical regions.

## Materials and methods

### Sample material

**Fig. 1:**
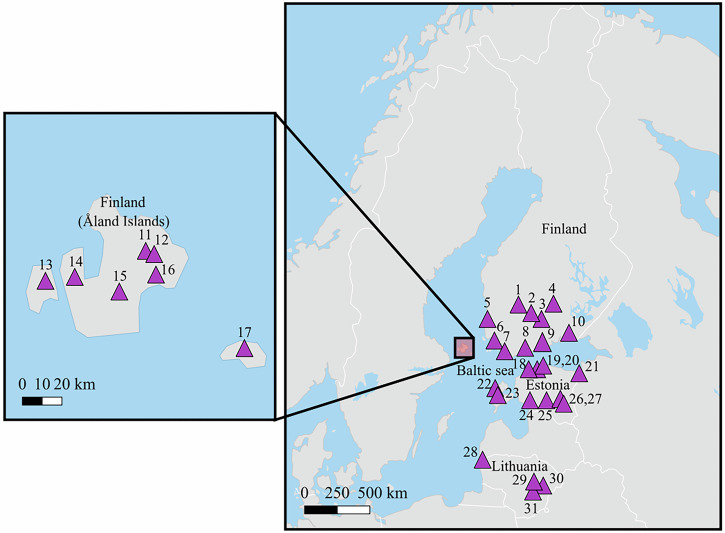
Sampling sites of the archaeological chicken (*Gallus gallus domesticus*) bones. Sites marked by triangles: 1 Pirkkala, 2 Hämeenlinna, 3 Janakkala, 4 Hartola, 5 Eurakoski, 6 Turku, 7 Hiittinen, 8 Raasepori, 9 Vantaa, 10 Kotkansaari, 11 Saltvik Kvarnbo Kohagen, 12 Saltvik Kvarnbo, 13 Eckerö, 14 Hammarland, 15 Jomala, 16 Sund, 17 Kökar, 18 Ilmandu, 19 Iru, 20 Jõelähtme, 21 Joaorg at Narva, 22 Saaremaa, Kurevere, 23 Saaremaa, Loona, 24 Pärnu, 25 Viljandi, 26 Tartu, 27 Lohkva, 28 Klaipėda, 29 Kernavė, 30 Vilnius, and 31 Trakai. For details, see Text S1 and Table S1.

Our sample set included 113 ancient chicken bone samples and 103 feather samples from modern landrace chickens (Table S1, Text S1). The ancient chicken bones were selected based on the availability of archaeological material for analysis and, especially for the Estonian samples, the existence of previous radiocarbon dating and stable isotope analyses was a consideration for the choice of specimens (Aguraiuja-Lätti et al. 2022; Ehrlich et al. 2021; Malve et al. 2023). Of the ancient bone samples, 69 were from Finland from the 3^rd^ to the 18^th^ century CE, including 26 from mainland Finland and 43 from the Åland Islands (an autonomous region in the Finnish archipelago). The Åland Islands are a group of islands around 100 km from mainland Finland (Fig. 1). Records of chicken transport between the mainland and Åland are scarce; hence, there may have been some levels of genetic isolation between chickens from these regions. Therefore, we handle these areas separately. Twenty-six ancient chicken samples came from Lithuania from the 13^th^ to the 16^th^ century CE and 18 from Estonia from the 7^th^ to the 20^th^ century CE, of which two originated from Saaremaa Island (Fig. 1, Text S1, Table S1). For context, the Iron Age in Finland, Estonia and Lithuania was from the 5^th^ century BCE to the beginning of the 13^th^ century CE. However, the Åland Islands use Swedish chronology, where the Iron Age ends in the middle of the 11^th^ century CE. The Iron Age was followed by the Middle Ages and the early modern period, which lasted until the 16^th^ and the 19^th^ century CE respectively in Finland, Estonia and Lithuania.

We collected 39 feather samples from all ten remaining lineages of the Finnish landrace chicken, with two to nine samples per lineage. Further, we collected 64 feather samples from Estonian landrace chicken populations, representing all remaining lineages and their mixes, with one to 23 samples per lineage/lineage mix.

### Radiocarbon dating

We selected 24 archaeological chicken bones among our samples for radiocarbon dating based on uncertain archaeological stratigraphy. For other samples, we used dating based on archaeological stratigraphy, and for six samples, radiocarbon dates were previously published (Table S1, Fig. S1). The radiocarbon dating was performed from 0.6–1 g of bone in the Vilnius Radiocarbon Laboratory using a Single Stage Accelerator Mass Spectrometer (SSAMS, NEC, USA), a Low Energy Accelerator (LEA, Ionplus AG, Zürich) and Automated Graphitization Equipment AGE-3 (Ionplus AG, Zürich). The samples were pre-treated with acid-base-acid procedure and collagen extraction according to Molnár et al. (2013). The radiocarbon ages were calibrated to calendar years using the OxCal 4.4 program (Bronk Ramsey 2009) with Bomb 13 NH1 (Hua et al. 2013) for aGal99 and aGal112, and IntCal20 (Reimer et al. 2020) for other radiocarbon-dated samples. To determine if the chickens had a marine component in their diet, which would require corrections in the radiocarbon dating results, stable isotopes of nitrogen and carbon were analysed from the same samples in the Vilnius Radiocarbon Laboratory using an Elementar Isoprime Vision mass spectrometer connected to a Vario Isotope Cube elemental analyser. Stable isotope values were below -20.0‰ for carbon (*δ*¹³C) and below 13.1‰ for nitrogen (*δ*¹⁵N), indicating no marine component in the diet (Olli et al. manuscript in preparation). Thus, corrections for the marine diet were not needed.

### DNA extraction and amplification of ancient samples

DNA was extracted from the chicken bone fragments as in Honka et al. (2018) in clean room facilities dedicated to aDNA. We used similar precautions as in Honka et al. (2018) to avoid contamination of the samples. Primer pair GG144F/GG387R from Storey et al. (2007) was used to amplify a 201-bp region of the mitochondrial control region. We designed primer pair Gal-TSHR-F/Gal-TSHR-R based on the chicken whole-genome sequence (assembly: bGalGal1.mat.broiler.GRCg7b) to amplify a 112-bp region containing the missense mutation in *TSHR* (glycine to arginine in residue 558; Rubin et al. 2010), suspected to be linked to shifts in seasonal mating between wild and domesticated chickens. Similarly, we designed primer pair Gal-BCDO2-F/Gal-BCDO2-R to amplify a 105-bp region containing the SNP found to be in complete association with yellow skin colour (chr24:6,273,428 adenine/guanine, in which the guanine is associated with the yellow skin colour; Eriksson et al. 2008).

PCR reactions were performed twice for all samples in 12.5 µL reaction volumes using 1× PCR buffer (HotStarTaq DNA Polymerase, Qiagen), 0.2 µM of each primer, 0.2 mM dNTPs, 1 mM MgCl_2_, 1 mg/mL BSA (Bovine Serum Albumin), 1 U of HotStarTaq DNA Polymerase (Qiagen) and 1 µL of extracted DNA. The thermal profile consisted of 95 °C for 15 min, followed by 55 cycles of 94 °C for 30 s, annealing temperature dependent on the primer pair (see Table S2) for 30 s, and 72°C for 30 s, with a final extension at 72 °C for 7 min.

PCR products were run on a 2.5% agarose gel to check for amplification. If amplification of a PCR band of the correct size and nonspecific bands occurred, the band with the correct size was extracted from the gel using GeneJET Gel Extraction Kit (Thermo Scientific) according to the manufacturer’s instructions. PCR products were sequenced using BigDye Terminator v.3.1 (Applied Biosystems) with 5 μM PCR primers in both directions for mitochondrial DNA and one direction for the nuclear genes (Table S2) and run on an ABI 3730 (Applied Biosystems). If two independent reactions did not yield successful sequences, PCR amplification and sequencing were repeated.

### Authentication

A strict workflow was followed to avoid modern DNA contamination of the ancient samples (Cooper and Poinar 2000). All bone fragments were handled in dedicated aDNA clean room facilities that are physically separated from modern DNA facilities at the Centre for Material Analysis at the University of Oulu, Finland. Sterile filter pipette tips were used, and all equipment and working surfaces were cleaned with bleach or DNA and RNA decontaminating DNA-ExitusPlus^TM^ (PanReac AppliChem). Bone drilling for DNA extraction was performed in a separate room from the rest of the DNA extraction steps and PCR setup to avoid contaminating samples with bone powder. Bone drilling was done in a fume hood that was UV sterilised prior to work. The PCR reaction set-up was performed in a separate dedicated UV-sterilising PCR workstation (Peqlab, Fareham, United Kingdom). Post-PCR work was performed in physically separated laboratory facilities at the University of Oulu. Negative controls were used in all laboratory steps to control for contamination of reagents, equipment, and samples. Negative controls from all steps produced no amplifications, indicating no contaminations in the reagents or equipment used and no contamination from other samples or modern DNA. PCR amplification and sequencing were performed in at least two independent reactions to identify post-mortem base modifications and sequencing errors. There were detectable post-mortem base modifications or polymerase errors in ancient sequences, which is expected from ancient samples. In these cases, the sample was amplified and sequenced again, and the correct base was determined as the base that was amplified from two independent reactions.

### DNA extraction and amplification of modern samples

To extract DNA from the modern landrace chicken feathers, the tip of the calamus, the membrane inside the calamus, as well as the blood clot between the calamus and rachis (Horváth et al. 2005) were cut into smaller pieces. QuickExtract^TM^ DNA Extraction Solution (Lucigen) was used for the extraction according to the manufacturer’s protocol with a few exceptions; depending on the amount of sample material, 100–300 μL of QuickExtract Solution was used instead of 0.5 mL, and samples were incubated at 65 °C for 15 min instead of 6 min.

The same three primer pairs used for the ancient samples were used to amplify DNA from the modern chicken samples (Table S2). PCR reactions were performed in 10 µL reaction volumes using 1× Qiagen Multiplex PCR Master Mix (Qiagen), 0.2 µM of each primer, RNase-free water (Qiagen), and 1 µL of extracted DNA. The thermal profile consisted of 95 °C for 15 min, followed by 40 cycles of 94 °C for 30 s, with the annealing temperature dependent on the primer pair (see Table S2) for 90 s, and 72°C for 90 s with a final extension at 72 °C for 10 min. Agarose gel electrophoresis and sequencing were performed similarly to the ancient samples (see DNA extraction and amplification of ancient samples).

### Sequence analysis of mitochondrial DNA

All ancient and modern sequences were edited using the program CodonCode Aligner v.4.0.4. (CodonCode Corporation, Centerville, Massachusetts, USA) by trimming low-quality ends of the basecalled sequences and correcting errors in basecalling by manually checking the chromatograms base-by-base. Sequences were aligned using the ClustalW algorithm (Thompson et al. 1994) implemented in the CodonCode Aligner. For mitochondrial reference haplotypes, we downloaded 448 modern sequences from GenBank (Table S3) and used haplogroup and haplotype naming according to Miao et al. (2013). In addition, we downloaded ancient reference sequences of 39 central and southern European chickens from Girdland Flink et al. (2014) and 5 ancient western Russian reference sequences from Dyomin et al. (2017) from GenBank (Table S3). Some ancient reference sequences were renamed following Miao et al. (2013) (Table S3). All sequences were aligned using the program BioEdit 7.7.1. (Hall 1999). The program PopART (Leigh and Bryant 2015) was used to construct a median-joining network (Bandelt et al. 1999) using zero as an ε value to assign haplotypes for the samples based on all reference sequences. Due to the large number of haplotypes in haplogroups, we simplified the haplotype network for clarity by showing one sequence per haplogroup, except for those containing our ancient samples, for which all matching modern reference sequences were included. Haplotype diversity (Nei 1987) values were calculated with DnaSP v.6.12.03 (Rozas et al. 2017). For haplogroup comparisons, we used ancient central and southern European chickens from Girdland Flink et al. (2014) and ancient western Russian chickens from Dyomin et al. (2017) and Lebrasseur et al. (2021) and modern European chickens from Miao et al. (2013) as reference haplogroup frequencies. For modern reference frequencies from Miao et al. (2013), the sampled chicken breed was not always reported, and therefore, the modern European reference set could include a small number of landrace chickens. In addition, we constructed a temporal statistical parsimony network for Finnish, Estonian and Lithuanian ancient samples and modern Finnish and Estonian landrace samples using the TempNet (Prost and Anderson 2011) R-script (R Core Team 2023).

### SNP analysis of *TSHR* and *BCDO2*

All ancient and modern sequences were edited using the program CodonCode Aligner v.4.0.4. (CodonCode Corporation, Centerville, Massachusetts, USA) similar to the mitochondrial sequences. The nuclear alleles were scored by eye from electropherograms. Allele frequencies of *TSHR* for comparison were taken from Rubin et al. (2010) for modern commercial chicken breeds and from Girdland Flink et al. (2014) for ancient central and southern European chickens. Allele frequencies of *BCDO2* for comparison were taken from Eriksson et al. (2008) for modern commercial chicken breeds and from Girdland Flink et al. (2014) for ancient central and southern European chickens.

## Results

### Radiocarbon dating

The radiocarbon ages of the samples analysed in this study varied from 1638 ± 34 BP (before present) to 191 ± 31 BP, and the calibrated calendar years varied from 266 – 541 cal CE to 1648 – modern cal CE with 95.4% probability (Table S1, Fig. S1). Two of the previously dated Estonian bones are dated to the late 20^th^ century (aGal99 and aGal112) (Ehrlich et al. 2021). The radiocarbon dating done for this study was mostly concordant with dating based on archaeological stratigraphy. Nine samples were around one to two centuries younger or older than expected based on archaeological stratigraphy, and only one sample was substantially older (aGal81, four centuries) than expected based on archaeological stratigraphy (Table S1). Other radiocarbon-dated samples matched or narrowed the dating based on archaeological stratigraphy (Table S1).

### Mitochondrial DNA haplotypes

The 201-bp long mitochondrial control region fragment was successfully amplified and sequenced from two independent reactions for 87 samples among the total of 113 ancient samples (77.0%) and for all 103 modern landrace chicken samples (Table S1). For ancient samples, there were differences in DNA preservation between samples from different geographic regions (Table S1). Twenty-three out of 26 Lithuanian samples (88.5%), 17 out of 18 Estonian samples (94.4%), and 35 out of 43 Finnish samples from Åland Islands (81.4%) yielded amplifiable mitochondrial DNA. Mitochondrial DNA preservation was the lowest among the samples from mainland Finland, as only 12 out of 26 samples (46.2%) yielded mitochondrial control region sequences.

**Fig. 2:**
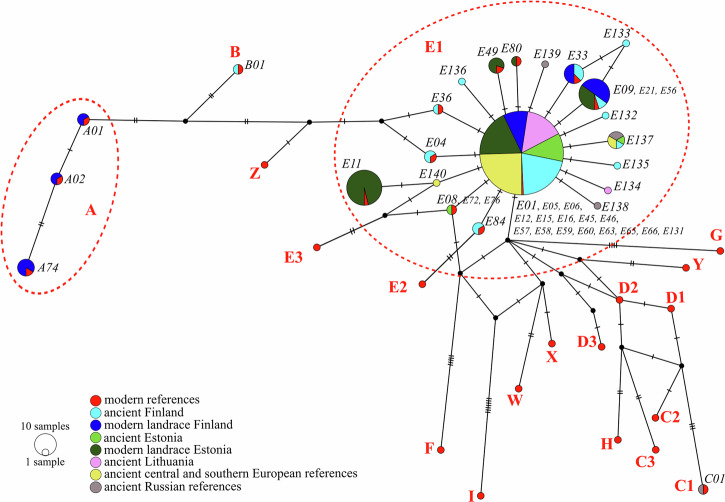
Median-joining haplotype network constructed from a 201-bp mitochondrial control region fragment of ancient and modern landrace chicken (Gallus gallus domesticus) samples, as well as reference sequences from central and southern European and western Russian ancient chickens and modern chicken breeds. Only the reference haplotypes that are identical to our samples are included for haplogroups A, B, and E1, and other haplogroups are presented as one haplotype for clarity of the network figure. The size of the circles is proportional to the number of samples having that haplotype. The bolded letters and dashed-lined circles represent the haplogroups, and letters in italics represent haplotypes. Tick marks represent mutational differences between haplotypes. See Fig. S2 for a haplotype network including all haplotypes from all haplogroups.

**Fig. 3:**
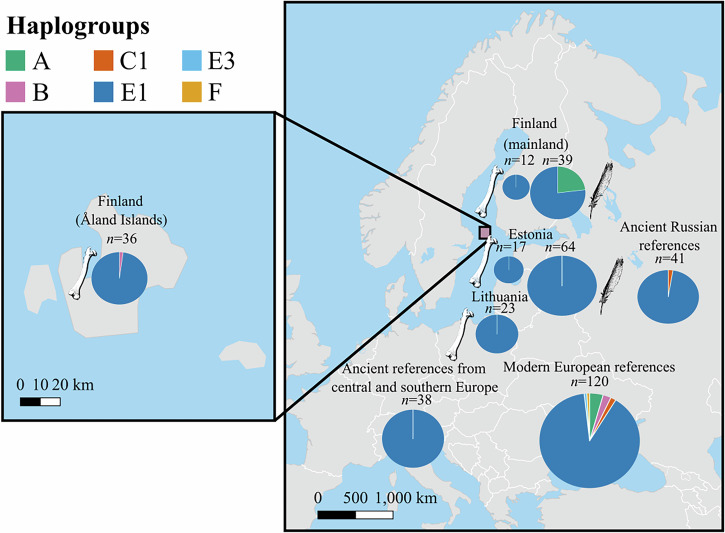
Mitochondrial haplogroups of ancient and modern chicken (*Gallus gallus domesticus*) samples from Finland, Estonia and Lithuania. The pie charts represent the frequencies of haplogroups pooled over the countries and the Åland Islands (Finnish archipelago). *n* stands for the number of individuals and is proportional to the size of the circle. The bone symbol indicates our ancient samples, and the feather symbol our modern landrace samples. For reference, we included haplogroup frequencies of ancient chickens from western Russia, central and southern Europe, as well as modern chickens from different European countries.

Due to using a short fragment, some haplotypes were identical within the studied region, but differ when using a longer mitochondrial DNA fragment as in Miao et al. (2013): E01 was identical with E05, E06, E12, E15, E16, E45, E46, E57, E58, E59, E60, E63, E65, E66 and E131, E08 was identical with E72 and E76, and E09 was identical with E21 and E56 (named according to Miao et al. 2013; Fig. 2, S2). Most of our ancient chicken samples (78.0% of successfully sequenced samples) from the three countries belonged to the E01/E05/E06/E12/E15/E16/E45/E46/E57/E58/E59/E60/E63/E65/E66/E131 group (hereafter referred to as the E01 haplotype for short). Among the ancient samples, five (aGal03, aGal17, aGal49, aGal58 and aGal66) had a unique haplotype not found among the modern or ancient reference sequences and were given haplotype names following the system in Miao et al. (2013) (Table S1). These sequences were closely grouped with the main ancient haplotype E01 and related haplotypes, indicating that they belong to haplogroup E1 (Table S1, Figs. 2, 3). All except one of our ancient samples (aGal81) belonged to haplogroup E1. This was a sample from the Åland Islands which was dated to 266-541 cal CE (1638 ± 34 BP) (Fig. S1, Table S1) and which had haplotype B01, belonging to haplogroup B. Modern Finnish landrace chickens mainly belonged to haplogroup E1, but nine samples out of the 39 (23.1%) belonged to haplogroup A (Table S1, Figs. 2, Fig. 3). All Estonian modern landrace chickens belonged to haplogroup E1 (Table S1, Figs. 2, 3). Haplotype numbers (*H*) varied from two in ancient Lithuanian samples to ten in the ancient Åland Islands samples (Table 1). Haplotype diversity values (*h*) ranged from 0.22 in ancient Estonian samples to 0.79 in modern Finnish landrace samples (Table 1).

**Table 1:** Mitochondrial haplotype number (*H*) and haplotype diversity (*h*) values for Finland (mainland and Åland Islands), Estonia and Lithuania from ancient chicken (*Gallus gallus domesticus*) bone samples and for the modern Finnish and Estonian landrace chicken populations from feather samples.

Region	Sample size	*H*	*h*
Ancient Finland (Åland Islands)	35	10	0.56
Ancient Finland (mainland)	12	4	0.60
Modern Finnish landrace	39	5	0.79
Ancient Estonia	17	3	0.22
Modern Estonian landrace	64	5	0.66
Ancient Lithuania	23	2	0.38

#### *TSHR*

**Fig. 4:**
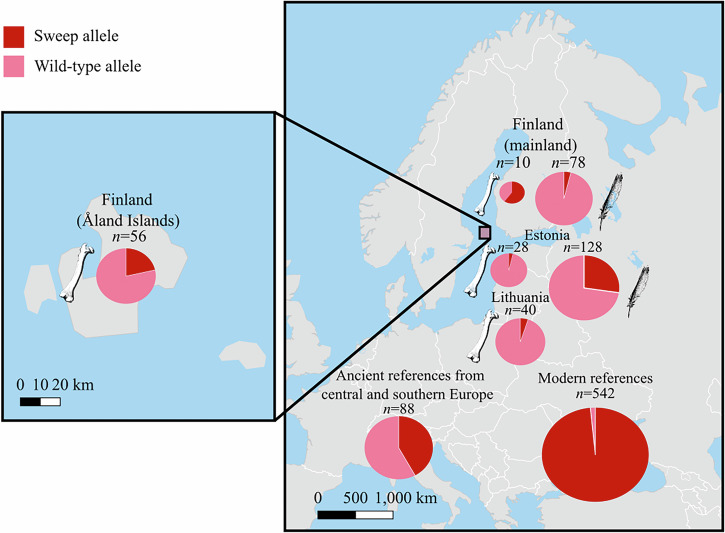
*Thyroid-stimulating hormone receptor* (*TSHR*) genotyping results from different regions around the Baltic Sea. The pie charts represent the frequencies of two alleles of the *TSHR* pooled over countries and the Åland Islands. *n* stands for the number of allele copies sampled, and the size of the circle is proportional to that. The bone symbol indicates our ancient samples and the feather symbol our modern landrace samples. For reference, we included allele frequencies of ancient chickens from central and southern Europe, as well as modern chickens.

All modern landrace chicken samples were successfully sequenced for the studied region of *TSHR*, and 67 of the 113 ancient samples (59.3%) yielded results for this fragment (Table S1). However, for eight ancient samples (11.9%), we obtained sequencing results only from one reaction, despite multiple attempts (Table S1). Among ancient samples, sweep allele (Arg) frequencies were 21.4% in Åland Islands (12/56), 60.0% in mainland Finland (6/10), 3.6% in Estonia (1/28), and 5.0% in Lithuania (2/40), with the remaining allele copies being wild-type (Gly) in each group (Fig. 4, Table S1). In modern landraces, sweep allele frequencies were 3.8% in Finland (3/78) and 27.3% in Estonia (35/128), with the remaining allele copies being wild-type in both groups (Fig. 4, Table S1).

The wild-type allele of the *TSHR* gene was the most prevalent among both our ancient chickens and modern landrace chickens, and only very few individuals were homozygous for the sweep allele (Table 2, Table S1, Fig. 4). Among the ancient chickens, heterozygosity levels were low (Table 2). Heterozygotes were rare (7.7%) among the modern Finnish landrace chickens, which were otherwise homozygous for the wild-type allele (92.3%). Modern Estonian landrace chickens had the highest number of heterozygous individuals (29.7%). Among ancient Finnish mainland chickens, 40.0% of individuals were homozygous for the sweep allele, but only five individuals had successful sequencing results. The second highest number of sweep allele homozygotes was in the modern Estonian landrace population (12.5%) (Table 2).

**Table 2:** *Thyroid-stimulating hormone receptor* (*TSHR*) genotyping results from Finland (mainland and Åland Islands), Estonia and Lithuania from ancient chicken samples and the modern Finnish and Estonian landrace chicken populations.

**Region**	**Sample size**	**Genotype**		
		**Arg/Arg**	**Arg/Gly**	**Gly/Gly**
Ancient Finland (Åland Islands)	28	3 (10.7%)	6 (21.4%)	19 (67.9%)
Ancient Finland (mainland)	5	2 (40.0%)	1 (20.0%)	2 (40.0%)
Modern Finnish landrace	39	0 (0%)	3 (7.7%)	36 (92.3%)
Ancient Estonia	14	0 (0%)	1 (7.1%)	13 (92.9%)
Modern Estonian landrace	64	8 (12.5%)	19 (29.7%)	37 (57.8%)
Ancient Lithuania	20	1 (5.0%)	0 (0%)	19 (95.0%)

The data tallies individuals homozygous and heterozygous for the sweep (Arg) and wild-type allele (Gly) and their percentage representation.

#### BCDO2

**Fig. 5:**
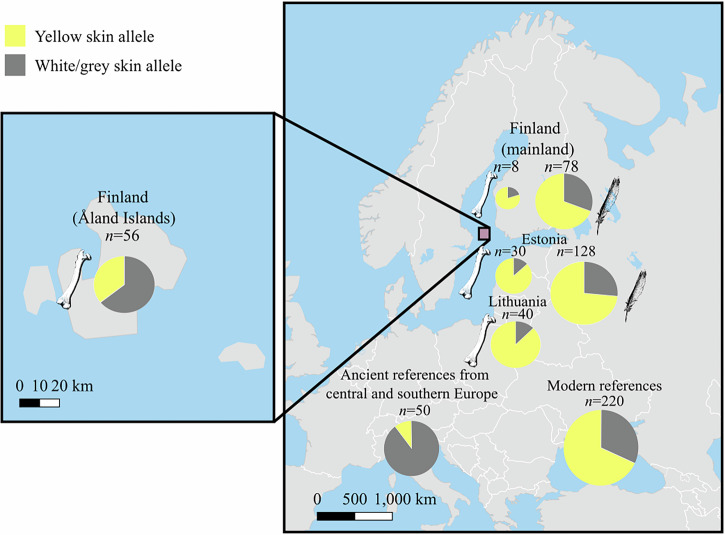
*β-carotene dioxygenase 2* (*BCDO2*) genotyping results from different regions around the Baltic Sea. The pie charts represent the frequencies of two alleles of the *BCDO2* pooled over countries and the Åland Islands. *n* stands for the number of allele copies, and the size of the circle is proportional to that. The bone symbol indicates our ancient samples, and the feather symbol our modern landrace samples. For reference, we included allele frequencies of ancient chickens from central and southern Europe, as well as of modern chickens.

All modern landrace chicken samples were successfully sequenced for the studied fragment of *BCDO2*, and 67 of the 113 ancient samples (59.3%) yielded results for this region. However, five (7.5%) of the ancient samples yielded sequences from only one independent reaction, despite multiple attempts (Table S1). Among ancient samples, yellow skin colour allele (Y) frequencies were 35.7% in Åland Islands (20/56), 87.5% in mainland Finland (7/8), 86.7% in Estonia (26/30), and 87.5% in Lithuania (35/40), with the remaining allele copies being white/grey skin colour allele (W) in each group (Fig. 5, Table S1). In modern landraces, yellow skin colour allele frequencies were 69.2% in Finland (54/78) and 73.4% in Estonia (94/128 alleles), with the remaining allele copies being the white/grey skin colour allele in each group (Fig. 5, Table S1).

The white/grey skin colour was more common among the ancient Åland Islands samples, whereas the yellow form was more common among both ancient and modern samples from mainland Finland and Estonia and the ancient samples from Lithuania (Tables 3, S1, Fig. 5). Only homozygotes for the yellow skin allele have a yellow skin colour. Therefore, 71.4% of ancient Åland Islands chickens, 25.0% of ancient mainland Finnish chickens, 38.5% of modern Finnish landrace chickens, 26.7% of ancient Estonian chickens, 32.8% of modern Estonian landrace chickens, and 15.0% of ancient Lithuanian chickens had white or grey skin colour (Tables 3, S1).

**Table 3:** *β-carotene dioxygenase 2* (*BCDO2*) genotyping results from Finland (mainland and Åland Islands), Estonia and Lithuania from ancient chicken samples and the modern Finnish and Estonian landrace chicken populations.

Region	Sample size	Genotype		
		W/W	W/Y	Y/Y
Ancient Finland (Åland Islands)	28	16 (57.1%)	4 (14.3%)	8 (28.6%)
Ancient Finland (mainland)	4	0 (0%)	1 (25.0%)	3 (75.0%)
Modern Finnish landrace	39	9 (23.1%)	6 (15.4%)	24 (61.5%)
Ancient Estonia	15	0 (0%)	4 (26.7%)	11 (73.3%)
Modern Estonian landrace	64	13 (20.3%)	8 (12.5%)	43 (67.2%)
Ancient Lithuania	20	2 (10.0%)	1 (5.0%)	17 (85.0%)

The data tallies individuals homozygous and heterozygous for the white/grey allele (W) and the yellow allele (Y) and their percentage representation.

## Discussion

Our oldest chicken sample from Finland was from the early Finnish Iron Age from the Åland Islands, dating to 266–541 cal CE (Table S1, Fig. S1). This is now the oldest radiocarbon-dated chicken finding from Finland, surpassing previously described samples from Salo and Levänluhta, in southern and western Finland, dating to 685 – 880 cal CE (Ukkonen and Mannermaa 2017; Wessman et al. 2018). This suggests that chickens were introduced to Finland as early as during the first few centuries of the Common Era, as they were to other northern European countries. This finding, as well as two Estonian samples previously radiocarbon dated (aGal99 and aGal112), were substantially older or younger than expected based on archaeological stratigraphy, which highlights the tendency of small bones to move through the stratigraphy. This phenomenon has also been observed in other chicken studies (Best et al. 2022; Ehrlich et al. 2021; Girdland Flink et al. 2014; Peters et al. 2022; for a review, see Peters et al. 2024). Because of this, direct radiocarbon dating should be performed to verify, for example, the earliest chicken findings from different regions. Direct radiocarbon dating would benefit the studies of other bird species as well, especially if the bone sizes are small.

Other Finnish samples range from the 9^th^ to the 18^th^ century CE based on radiocarbon dating and archaeological context (Table S1, Fig. S1). For our Estonian and Lithuanian samples, the radiocarbon and archaeological stratigraphy dating range from the 7^th^ to the 20^th^ century CE and from the 13^th^ to the 16^th^ century CE, respectively. The oldest chicken findings are from 200 cal BCE–5 cal CE in Estonia (Ehrlich et al. 2021) and 200 BCE–200 CE in Lithuania (Vėlius et al. 2025), although the earliest Lithuanian finding has not been radiocarbon dated.

Most Finnish chicken samples from the mainland did not yield amplification of the studied nuclear SNPs, and over half of the samples yielded no amplifiable mitochondrial DNA (Table S1). This was expected since conditions in mainland Finland are not ideal for DNA preservation due to the acidity of the soil. On the other hand, the Åland Island samples showed much better DNA preservation, with over 80% of samples yielding amplifiable mitochondrial DNA, which was expected since the soil is more calciferous than in mainland Finland. DNA preservation was also good in Estonia and Lithuania.

All ancient chickens studied, except one, belonged to mitochondrial haplogroup E1 (Fig. 3, Table S1). All ancient European chickens reported in previous studies, and all except one western Russian chicken, also belonged to haplogroup E1 (Dyomin et al. 2017; Girdland Flink et al. 2014; Lebrasseur et al. 2021). Based on this, the currently most common haplogroup E1 has been the main haplogroup for most of the chicken breeding history in Europe. Possibly the first chickens brought to Europe and western Russia belonged to haplogroup E1, or in the case of several independent introduction events, chickens carrying the E1 haplogroup were always introduced, or only chickens with this haplogroup were bred on a large scale. While haplogroup E1 is the most common haplogroup among modern chickens in Europe, haplogroups A, B, C1, E3, and F are found in Europe as well, although in lower frequencies (Miao et al. 2013). Haplogroup A has been found in the United Kingdom, Hungary, and the Netherlands, haplogroup B in Germany and Hungary, and haplogroups C1, E3, and F in the Netherlands (Miao et al. 2013). Interestingly, one Finnish archaeological sample from the Åland Islands originating from Kvarnbo settlement in Saltvik municipality had haplotype B01 of haplogroup B (Fig. 3, Table S1). This bone was excavated from a stone-lined post hole (Text S1). This is the earliest finding of any haplogroup other than E1 in Europe since this sample was dated to 266–541 cal CE (Early Iron Age in the Finnish chronology), representing also the oldest radiocarbon-dated chicken bone in Finland (Fig. 3, Table S1). As haplogroup B is distinct from haplogroup E1 (Fig. 2 and S2), its presence in Åland could indicate that this individual has been brought from another population than the other European ancient chickens. One possibility for the location of the source population could be Sweden, as it is close to the Åland Islands, but as no aDNA results of Swedish chickens are available, this cannot be verified. Because no other individuals from this haplogroup have been reported, our finding suggests that only a few individuals of this haplogroup were introduced or that individuals of this haplogroup were not bred on a larger scale. Previously, only one other ancient chicken sample close to our study region has been reported to carry a haplogroup other than E1. This one was from Pskov, western Russia, from the 18^th^ century cal CE, carrying haplogroup C1 (Dyomin et al. 2017).

Most modern Finnish landrace chickens also belonged to haplogroup E1, but nine samples had haplogroup A (Fig. 3, Table S1). Haplogroup A was found in Piikkiö, Ilmajoki, Kiuruvesi, Savitaipale, and Alho lineages, but due to limited sample sizes of the ten landrace lineages, the presence of this haplogroup in other lineages cannot be excluded (Table S1). Since haplogroup A was not found among the ancient Finnish mainland samples, which have been dated to the 10^th^–18^th^ century CE, this could indicate that after the 18^th^ century, chickens carrying haplogroup A were brought to Finland and mixed into the landrace lineages. Haplogroup A is common in modern Asian chickens, while in Europe it has been found in the United Kingdom, Hungary, and the Netherlands (Miao et al. 2013). The Finnish chicken caretaking book from 1900 lists several foreign breed groups, such as Asian breeds, tufted breeds, Dorking chickens, Mediterranean chickens and Hamburg chickens (Forselius 1900), making it possible that the A haplogroup was acquired from foreign breeds. Another explanation is that due to the small sample size among the ancient mainland Finland samples, the haplogroup remained undetected. Landrace chicken lineages from the Åland Islands no longer exist, and haplogroup A was not found among the ancient samples from the Åland Islands.

All modern Estonian landrace chickens belonged to haplogroup E1, as did the ancient Estonian samples. But not all haplotypes found among modern landrace samples were found among the ancient Estonian chickens, and the haplotype diversity value for modern Estonian landrace chickens was notably higher than for ancient Estonian chickens (Fig. 3, Tables S1, 1). This could either indicate that chickens with more diverse haplotypes were brought to Estonia quite recently (our bone samples are from the 8^th^–20^th^ century CE) or that these haplotypes were present during ancient times but were not found among our samples. The elimination of these competing hypotheses is not possible due to issues related to archaeological material, such as small and non-random sampling and DNA preservation.

It has been suggested that the SNP in *TSHR* is linked to shifts in seasonal mating, causing earlier egg laying at sexual maturity, as well as reduced aggressiveness towards other chickens and diminished fear of humans. This allele had differing frequencies in the ancient Finnish samples from the Åland Islands and mainland, and ancient Finnish samples differed from ancient Estonian and Lithuanian samples, as the sweep allele had a higher frequency in ancient Finland. However, in all countries, the wild-type allele was more common, with the exception of mainland Finland, but as noted earlier, the sample size for this region is small (Fig. 4, Table S1). Among the central and southern European ancient chickens, the sweep allele, for which the modern breeds are mainly homozygous, was almost as common as the wild-type allele (Girdland Flink et al. 2014). This could imply that the sweep allele was selected for in central and southern Europe, while there was less intensive selection in Finland and no selection in Estonia and Lithuania. Another explanation is that the chickens of the Baltic Sea region had a different origin from the more southern chickens. There are no published data for this gene from any closely located regions for comparison.

The modern Finnish landrace chickens had a lower frequency of the sweep allele than the ancient Finnish samples from the mainland or the Åland Islands. However, the small sample size of mainland Finland needs to be taken into consideration, as well as the fact that there are no landrace chicken lineages from the Åland Islands. The majority of the modern Finnish landraces are homozygous for the wild-type allele, while only a few are heterozygotes, and none are homozygous for the sweep allele. This may have resulted from a small number of founder individuals, a population bottleneck, human selection or random drift. The modern Estonian landrace chickens had a higher frequency of the sweep allele than the modern Finnish landrace chickens or the ancient Estonian ones. Still, the wild-type allele is the main allele in the modern landrace chickens in both countries (Fig. 4, Tables 2, S1). Possibly, the Estonian landrace chicken populations originate from more heterogeneous populations than the Finnish landrace chickens.

Among ancient chickens from the Åland Islands, the most common phenotype at the *BCDO2* locus was white/grey skin colour, whereas in mainland Finland (with a limited sample size), Estonia and Lithuania, the majority of ancient samples were homozygous for the yellow skin allele (Table 3, Fig. 5, Table S1). Most ancient central and southern European chickens have been found to be homozygous for the white/grey allele, and only some were heterozygotes; thus, none expressed yellow skin, as it is a recessive trait (Girdland Flink et al. 2014). This suggests that samples from the Åland Islands were more similar to central and southern European samples than to Estonian and Lithuanian samples for this trait. Possibly, in Estonia and Lithuania, the yellow skin allele, which is the main form for modern breeds, was under selection earlier than in the Åland Islands and central and southern Europe, since the samples from different regions are mainly from the same periods. This could imply that in part of their European range, chickens have been selected for yellow skin colour quite early on. It has been suggested that the yellow skin colour was favoured during the formation of modern breeds because people considered yellow skin colour as an indicator for higher nutrient content, health and absence of avian diseases (Ndenga et al. 2017; Sunde 1992). Perhaps the people in the Baltic region considered the skin colour more strongly as an indication of meat quality. The differing allele frequencies could also be due to chickens being brought to the Åland Islands from a different region or a different group of domestic chickens than the Estonian and Lithuanian chickens, or due to a lack of selection for skin colour. Denser sampling of Europe and western Russia is needed to verify whether these differences arise from artificial selection or different origins of the chickens.

Most of the modern Finnish landrace chickens were homozygous for the yellow skin allele of the *BCDO2* gene (Table 3, Fig. 5, Table S1). Three leg/skin colours are known from modern Finnish landrace chickens: ivory, grey and yellow. The number of ancient chickens genotyped for this trait in mainland Finland was low (4 individuals), and therefore, reliable comparisons cannot be made. In addition, the ancient Finnish samples from the Åland Islands may not be representative of the ancient mainland population’s allele frequencies. Estonian modern landrace chickens, however, have similar frequencies of the two alleles as the ancient Estonian chickens (Table 3, Fig. 5, Table S1).

Based on mitochondrial DNA and allele frequencies of *TSHR* and *BCDO2*, it appears that the modern Estonian landrace chickens (sampled in 2024) resemble the ancient Estonian chickens (from the 7^th^ to the 20^th^ century CE) more than the modern Finnish landrace chickens (sampled in 2024) resemble the ancient Finnish chickens (from the 3^rd^ to the 18^th^ century CE) from the mainland and Åland Islands. This suggests that in Estonia, the landrace chicken lineages may have remained more similar than in Finland during their breeding history, which is surprising considering the history of several Estonian cities as important Hanseatic centres with multiple international contacts. Possibly, after the 18^th^ century, more diverse chickens were brought to Finland and mixed with the landrace chicken lineages, since mitochondrial haplogroup A is only found in modern samples, and the allele ratios of *TSHR* and *BCDO2* differ considerably among modern and ancient samples from Finland. Since haplogroup A is common in Asia and has been found in the United Kingdom, Hungary, and the Netherlands (Miao et al. 2013), chickens were perhaps brought to Finland from these regions. Chicken breed development in the 19^th^ century in Europe included the import of Asian breeds, exemplified by several Asiatic chicken breed groups listed in the Finnish chicken breeding guidebook (Forselius 1900), and thus this may present a plausible source of haplogroup A in modern Finnish landrace chickens. Forselius (1900) even encourages crossbreeding of Finnish landrace chickens with foreign breeds to establish robust chicken lineages for the less wealthy people. In addition, landrace chickens from Finland and Estonia differ notably from modern production breeds based on mitochondrial DNA and *TSHR* and *BCDO2* allele frequencies. Similar comparisons for Lithuanian samples cannot be made, as there are no landrace chickens in Lithuania, but the ancient Lithuanian chickens resemble more the ancient Estonian than the ancient Finnish chickens or modern landrace chickens from Estonia or Finland.

Our ancient dataset covers almost two thousand years of chicken history in northern Europe, but as the time range for different countries varies and the majority of our samples are from the Middle Ages (Fig. S3), we cannot reliably estimate temporal variation within this time frame. Only a small number of samples are from the Iron Age or early modern period, and they give similar results for the nuclear SNPs typed as the medieval samples, but the small sample size could bring bias and hide possible temporal variation. To more accurately estimate temporal variation, more samples from the underrepresented time frames would be needed.

When the ancient Finnish chickens from the Åland Islands and mainland are compared, they differ from each other, especially at *TSHR* and *BCDO2* allele frequencies, which could indicate that chickens were not frequently moved between these regions, and therefore, some level of genetic isolation may have existed. These differences could also be caused by the bias of a small sample size from mainland Finland. There is a record of 74 chickens being sent from Kastelholm Castle on the Åland Islands to Turku Castle on the mainland in 1562 (KA 735, National Archives of Finland). However, this was a case of resource movement between castles based on needs and not a regular action, and since other records of chickens being moved between the Åland Islands and the mainland are scarce, the frequency and scale of chicken transport between these regions remain unknown. In addition, a few records dating to times when Finland was under Swedish rule (from the 12^th^ to the early 19^th^ century, the same period as for our samples) show that chickens from the Åland Islands were exported to Sweden to be sold in Stockholm (Kivikero 2025). This suggests the possibility of chickens being transferred also from Sweden to the Åland Islands. If this was more frequent than the transport between the Åland Islands and mainland Finland, it could have promoted divergence of the chicken populations on the Åland Islands and in mainland Finland. When Finnish chickens, especially from Åland Islands, are compared to those from ancient Estonia and Lithuania, they are rather different based on *TSHR* and *BCDO2*, whereas the Estonian and Lithuanian ancient chickens seem to be very similar to each other (Tables 1 and 2, Figs. 4 and 5, Table S1). This indicates that the Finnish chickens were, to some extent, isolated from Estonian and Lithuanian chickens, and there was perhaps little or no trade of chickens between Finland and the Baltic countries. Within the Baltic countries, chickens were perhaps transported more because land transport would have been easier than crossing the Baltic Sea (Gulf of Finland). It is also possible that Finnish and Baltic chickens had a different origin. Perhaps the Baltic chickens have a more eastern origin than the Finnish chicken, which could have their origin in the west (Åland Islands) or the northern regions of Russia (mainland Finland). Since the Åland Islands samples differ clearly from the other regions, and one of the oldest chickens from the Åland Islands had a different mitochondrial haplogroup than other ancient samples, the import of chickens from Sweden may have played a role in the characteristics of the Åland Islands’ population. To make more precise conclusions, samples from other surrounding European and Russian regions, as well as genome-wide studies, are needed. For example, approaches such as admixture modelling or whole-genome sequencing of archaeological samples would provide deeper insights into population relationships beyond mitochondrial data and a few nuclear loci. In addition, comparisons with SNP panel data from modern Finnish landraces, such as those reported by Berres et al. (2020), may also allow for a more comprehensive assessment of the genetic continuity of landrace lineages.

## Conclusions

Based on our results, ancient Finnish chickens, especially from the Åland Islands, differed genetically from other Baltic chickens, as indicated by allele frequencies of the *TSHR* and *BCDO2* genes, despite these regions being separated only by the narrow Gulf of Finland. Chickens from the Åland Islands were the most distinctive, also harbouring a mitochondrial haplogroup not detected in any other ancient chicken sample thus far. North European chickens also appear to differ from central and southern European chickens from the same periods for *TSHR* and *BCDO2*. The landrace chicken lineages in Estonia are more similar to ancient ones than landrace lineages in Finland, suggesting that perhaps recently, more diverse chickens were brought to Finland and mixed into the landrace lineages. Landrace chickens in Finland and Estonia differ from the modern production breeds. Thus, native landrace breeds are important reservoirs of genetic diversity for the production breeds. This reservoir is needed, for example, to provide disease resistance, to lower levels of inbreeding and inbreeding depression, and to safeguard future adaptations to the changing environment.

## Supplementary information

Supplementary information

Supplementary information

Supplementary information

## Data Availability

Obtained sequences of mitochondrial control region (identical sequences represented by one sequence) are deposited into GenBank with accession numbers: PV459710–PV459728. The sequenced nuclear SNPs of *TSHR* and *BCDO2* are given in Table S1.
