# Supplementary material for: Ancient DNA unravels the history of chickens in the Baltic Sea region and the continuity of landrace lineages
Source: Heredity (Edinb). 2026 Apr 13;135(6):397–408. doi: 10.1038/s41437-026-00842-9 (PMC13354571; doi:10.1038/s41437-026-00842-9)
Supplement: Supplementary file 1 — Supplementary information [file 41437_2026_842_MOESM1_ESM.docx]

**Supplementary information**

**Text S1.** Archaeological context of sampling sites

**Finland (Mainland)**

**Pirkkala, Tursiannotko** is a Late Iron Age and Early Medieval (c. 700–1300 CE) rural inland settlement site located in the southern part of Finland. The site was excavated between 1987 and 2022. Two chicken bones (aGal04, aGal05) were sampled from excavations conducted by Sami Raninen in 2012 from blackish grey clay soil (Y1) interpreted to be an activity layer, as well as under a wooden structure (possibly a floor) connected to the blackish grey soil (Raninen, 2013). The samples come from material stored at the Finnish Heritage Agency under collection numbers KM39258:936, KM 39258:950.

**Hämeenlinna, Varikkoniemi** is a Late Iron Age and Early Medieval (c. 800–1300 CE) rural settlement site in the central part of Finland. The site was excavated by Eeva-Liisa and Hans Peter Schulz between 1986 and 1993. One chicken bone (aGal02) was sampled from excavations conducted in 1987 from a building (structure 3) dated to the later phase (c. 1000–1300) of the settlement site (Schultz & Schultz, 1989). The sample comes from material stored at the Finnish Heritage Agency under collection number KM23703:1980.

**Hämeenlinna, Hattelmala** is an Iron Age (c. 500 BCE–1150 CE) rural settlement site in the central part of Finland. The site was excavated by Eeva-Liisa and Hans Peter Schulz in 1989. One chicken bone (aGal39) was sampled from a context described as the topsoil in the outskirts of the interpreted settlement site (Schultz & Schultz, 1990). The sample comes from material stored at the Finnish Heritage Agency under collection number KM24743:468.

**Janakkala, Hakoinen Castle** is a medieval (c. 1150–1500 CE) hillfort located in the central part of Finland. The site was excavated by Jouni Rinne in 1909. Two chicken bones (aGal18, aGal21) were sampled: one from a structure located inside the main castle and the other inside the castle walls in a structure interpreted to be a well (Rinne, 1909). The samples come from material stored at the Finnish Heritage Agency under collection numbers KM5455:21, KM5455:74.

**Hartola, Uusi-Ruskeala** is a rural settlement site in southern Finland, dated from the Late Iron Age to the early modern period (c. 1000–1700 CE). The site was excavated by Petro Pesonen in 2009. One chicken bone (aGal40) was sampled from a structure (8b) interpreted likely to be a trash pit (Pesonen, 2010). The sample comes from material stored at the Finnish Heritage Agency under collection number KM37985:58.

**Eurakoski, Eurajoki Liinmaa Castle** is a medieval (c. 1300–1500 CE) castle and settlement site located on a small island in the south-western coast of Finland. The site was excavated in 1886 by Hjalmar Appelgren. Three chicken bones (aGal08, aGal09, aGal10) were sampled from a ditch that was excavated on the southern corner of the castle courtyard (Appelgren, 1886). The samples come from material stored at the Finnish Heritage Agency under collection numbers KM2498:16.

**Turku, Aboa Vetus** is a town plot from the middle of the early modern period (c. 1500–1550 CE) in the town of Turku. The site is located on the premises of Aboa Vetus/Ars Nova Museum and was excavated in 2014 and 2015 as a part of citizen science outreach of the museum (Lehtonen & Aalto, 2015). Three chicken bones (aGal06, aGal07, aGal08) were sampled from a context interpreted to be a kitchen. The samples are stored at the Aboa Vetus/Ars Nova Museum.

**Hiittinen, Högholmen**is a medieval (c. 1150–1500 CE) settlement site on an island in the Archipelago Sea near the city of Turku. The site has been interpreted to have trading activity. The bone material comes from the top of the hill at Högholmen, where excavations were carried out inside building remains by Torsten Edgren during 1974, 1975 and 1976 (Edgren, 1996). Two chicken bones (aGal15, aGal65) were sampled from the later excavated materials. The samples come from material stored at the Finnish Heritage Agency under collection number KM77058:128.

**Raasepori, Junkarsborg** is a medieval hillfort (c. 1350–1450 CE) located on the coast of southern Finland. The site was excavated by Jouko Voionmaa in 1951, who left little documentation and no reports of the work he did. Archaeologist Tarja Knuutinen has reconstructed large parts of the excavated material, which likely comes from the inside of the bank surrounding the main castle, on the north-eastern part of the castle. The castle was in use during a short period at the end of the 14^th^ century and the beginning of the 15^th^ century, based on ceramics finds. More recent finds dated to the 18^th^ and 19^th^ centuries have been identified in the material, indicating also a more recent use of the site. Six chicken bones (aGal13, aGal14, aGal16, aGal17, aGal19, aGal20) were sampled from uncertain contexts from the site. The samples come from material stored at the Finnish Heritage Agency under collection numbers KM351100:61, KM51092:20, KM51092:29, KM51092:35, KM52106:28, KM53110:14.

**Vantaa, Gubbacka** is a hamlet on the southern coast of Finland, dated to the medieval and early modern period (1400–1600 CE). The sample comes from excavations conducted by Veli-Pekka Suhonen in 2003. One chicken bone (aGal41) was sampled from a cultural layer (Y213) related to a square stone structure (R203) interpreted to be an oven (Suhonen, 2003). The sample comes from material stored at the Finnish Heritage Agency under collection number KM2003102:643.

**Kotka, Kotkansaari** is a late early modern and modern (c. 1790–1850 CE) urban settlement site located on the south-eastern coast of Finland. The site was excavated by Marita Kykyri in 2012. Two chicken bones (aGal01, aGal03) were sampled from lower cultural layers from a building that was used as a military headquarters and a senior officers’ building (Kykyri, 2013). The samples come from material stored at the Finnish Heritage Agency under collection numbers KM39174:262, KM39174:263.

**Finland (Åland Islands)**

**Saltvik, Kvarnbo Kohagen** **Sa 14.7** is an Iron Age (c. 650–1050 CE) rural settlement site located on the main island. The site was excavated in 1958 by Matts Dreijer and 1960 Stig Dreijer. Six Iron Age buildings with visible stone foundations and a small area outside of them were excavated. Three chicken bones (aGal82, aGal83, aGal84) were sampled from building 5 and from an area with cultural layers west of house 5 (Drejer, 1958; Drejer, 1960). The samples come from material stored at the Ålands Museum under collection numbers ÅM328:72, ÅM347:85, ÅM347:131.

**Saltvik, Kvarnbo** **Sa 14.9** is an Iron Age (c. 650–1050 CE) rural settlement site located on the main island. The site was excavated by Kristin Ilves in 2016 and has several buildings represented by postholes dating from c. 400 CE to 1050 CE. One chicken bone (aGal81) was sampled from a stone-lined post hole A276, not connected to any specific building (Ilves, 2017). The sample comes from material stored at the Ålands Museum under collection number ÅM783:985.

**Eckerö, Thors** **E 3.5** is an early modern period (c. 1600–1800 CE) rural settlement site located on the western part of the islands. The site with cultural layers and the remains of a burned building was excavated by Rudolf Gustavsson in 2007. One chicken bone (aGal86) was sampled from layers connected to the burned building (Gustavsson, 2009). The sample comes from material stored at the Ålands Museum under collection number ÅM739:107.

**Hammarland, Kattby** **Ha 18.4** is an Iron Age (c. 650–1050 CE) rural settlement site located on the northern part of the main island. The site was excavated by Milton Núñez in 1973. Besides dwellings and everyday activities, the site has remains from bronze and antler working. One chicken bone (aGal70) was sampled from cultural layers, at 20–30 cm depth (Núñez, 1973). The sample comes from material stored at the Ålands Museum under collection number ÅM459e:760.

**Jomala, Brömsängsbacken** **Jo 10.3** is an Iron Age (c. 650–1050 CE) rural settlement site located on the main island. The site was excavated by Björn Cederhvarf in 1904 and 1914. The site has several buildings with visible stone foundations. Two chicken bones (aGal79, aGal80) were sampled from building VIII and cultural layer A in square 162, respectively (Cederhvarf, 1904; Cederhvarf, 1914). The samples come from material stored at the Finnish Heritage Agency under collection numbers NM4617:231, NM6769:460.

**Sund, Kastelholm Castle** is a medieval and early modern period castle (c. 1350–1700 CE) located on the main island. The site has been excavated several times during the past 200 years. For the study, 28 chicken bones were sampled from three areas that were excavated between 1983 and 1985. One of the areas (KS5) was outside the north-eastern wall from where 12 samples (aGal53, aGal55, aGal56, aGal58, aGal59, aGal61, aGal62, aGal63, aGal67, aGal68, aGal73, aGal75) were taken from KS5 excavated in 1983, together with four samples (aGal51, aGal66, aGal77, aGal88) from the same area excavated in 1984 (Carlsson 1987). The other two areas (KS8 and KS35) were located in the castle courtyard. One sample (aGal90) was taken from KS35 (Elfwendahl & Åqvist, 1987) and seven samples (aGal52, aGal54, aGal64, aGal71, aGal76, aGal85, aGal91) from KS8 excavated in 1983 (Elfwendahl, 1988). One sample (aGal74) was taken from a chicken bone from the Tower (inside the castle) excavated in 1980 (Törnblom, 1980). The samples come from material stored at the Ålands Museum under collection numbers ÅM516:17, ÅM542:2, ÅM542:6, ÅM542:12, ÅM542:16, ÅM542:29, ÅM542:30, ÅM542:31, ÅM542:80, ÅM542:159, ÅM542:161, ÅM542:191, ÅM542:195, ÅM542:221, ÅM542:227, ÅM542:242, ÅM542:256, ÅM542:264, ÅM542:370, ÅM556:1, ÅM565:4, ÅM565:8, ÅM565:26.

**Kökar Friary/Presbytery** is a medieval Franciscan friary which, after the Reformation, functioned as a presbytery located on the outer archipelago of the Åland Islands. Six chicken bones (aGal57, aGal60, aGal69, aGal78, aGal87, aGal89) were sampled from a building interpreted to be a refectory, excavated between 1982 and 1983 by Kenneth Gustavsson (Gustavsson, 1994). The samples come from material stored at the Ålands Museum under collection numbers ÅM538:53, ÅM538:204, ÅM553:94, ÅM553:151.

**Estonia**

**Ilmandu III *tarand*-grave** is a Bronze Age stone grave in northern Estonia. The context of the animal bones in stone graves can be unclear, i.e. whether they are contemporaneous with the burials. Several animal specimens from Estonian stone graves have been radiocarbon dated to later periods than the Bronze Age, showing the mixed nature of the sites (e.g. Rannamäe et al., 2016). The same applies to the analysed chicken bone (aGal100, specimen ID AI-6009/AZ-14:2) from the Ilmandu III *tarand*-grave, which has been radiocarbon dated to the Pre-Viking Age around 665–775 cal CE (Ehrlich et al., 2021, Table 1). The background for this chicken bone is thus unknown.

**Iru** settlement/hillfort site in northern Estonia includes archaeological evidence from the 3rd millennium BCE until the 11th century CE (Lang, 1996; Tõnisson, 2008, 187 and references therein). Mixed contexts have made it difficult to assign animal remains to specific periods. The analysed chicken bone (aGal96, specimen ID AI-5302/1986/AZ-2:001) was radiocarbon dated to the Viking Age around 675–950 cal CE (Ehrlich et al., 2021, Table 1), and thus originates from the period of the site when it was in active use as a fort (Tvauri 2012, 45–46). Most likely, the chicken bone is part of food remains.

**Jõelähtme stone-cist cemetery** is a Middle Bronze Age burial site in northern Estonia. The context of the animal bones in stone graves can be unclear, i.e. whether they are contemporaneous with the burials. Several animal specimens from Estonian stone graves have been radiocarbon dated to later periods than the Bronze Age, showing the mixed nature of the sites (e.g. Rannamäe et al., 2016). The same applies to the analysed chicken bone (aGal99, specimen ID AI-5306/AZ-1148) from Jõelähtme, which is modern according to radiocarbon dating (Ehrlich et al., 2021, Table 1). The background for this chicken bone is thus unknown.

**Joaorg at Narva settlement/hilltop site** in north-eastern Estonia was inhabited from the Mesolithic to the Modern Period (Ehrlich et al., 2021, 166 and references therein). The analysed chicken bone (aGal98, specimen ID AI-4101/1957/AZ-1) is early modern/modern, around 1675–1945 CE according to radiocarbon dating (Ehrlich et al., 2021, Table 1) and is probably part of food refuse of the settlement inhabitants.

**Kurevere stone grave cemetery** is a burial ground in Saaremaa Island, western Estonia. It was used as a burial ground from the Late Bronze Age to the Late Iron Age (Ehrlich et al., 2021, 165 and references therein). The context of the animal bones in stone graves can be unclear, i.e. whether they are contemporaneous with the burials. Several animal specimens from Estonian stone graves have been radiocarbon dated to later periods than the Bronze Age, showing the mixed nature of the sites (e.g. Rannamäe et al., 2016). The same applies to the analysed chicken bone (aGal112, specimen ID AI-1394/1877/AZ-1) from Kurevere, which is modern according to radiocarbon dating (Ehrlich et al., 2021, Table 1). The background for this chicken bone is thus unknown.

**Loona settlement** is a Neolithic and Early Bronze Age settlement in Saaremaa Island, western Estonia. The settlement site was partly disturbed by a Bronze and Early Iron Age stone-cist grave and a 13th–14th century CE cemetery, making it impossible to date animal remains by context (Ehrlich et al., 2021, 165–166 and references therein). The analysed chicken bone (aGal97, specimen ID AI-4129/1958/AZ-1) is medieval/early modern, around 1505–1795 cal CE according to radiocarbon dating (Ehrlich et al., 2021, Table 1). The background for this chicken bone is thus unknown.

**Pärnu, Põhja St** is an urban site in south-western Estonia, in the town of Pärnu. The analysed chicken bone (aGal109, specimen ID PäMu-A-2570/AZ-31:1) comes from an early modern context, dated by associated finds to the 17^th^ and 18^th^ centuries (Rannamäe et al., 2023, Table 1), and would thus be associated with food remains of the urban inhabitants.

**Viljandi Castle** is a castle of the Teutonic Order in southern Estonia, one of the most powerful in the territory of present-day Estonia. The two analysed chicken bones (aGal101, specimen ID VM-10922/AZ-71:13; aGal106, specimen ID VM-10846/AZ-16:1) are both medieval and represent most likely food refuse (Haak & Valk 2002; Haak 2004).

**Viljandi, Pikk 4** is an urban site in southern Estonia, in the town of Viljandi. The analysed chicken bone (aGal105, specimen ID TÜ-3007/AZ-1:285) comes from a medieval deposit, dated by context from the mid-13th to the beginning of the 14th century (Haak et al., 2012), and would thus be associated with medieval food remains of the urban inhabitants.

**Viljandi, Laidoneri väljak 10** is an urban site in southern Estonia, in the town of Viljandi. The analysed chicken bone (aGal107, specimen ID VM-10942/AZ-20:496) comes from a medieval deposit, dated by context from the 2nd half of the 13th to the beginning of the 15th century (Rannamäe et al., 2023, Table 1), and would thus be associated with medieval food remains of the urban inhabitants.

**Tartu, Jakobi 2** is an urban site in southern Estonia, in the town of Tartu. The analysed chicken bone (aGal102, specimen ID TM-A-188/AZ-12:223) comes from a medieval layer of the 13th to 14th century, dated by associated finds (Tvauri 2011), and would thus be associated with medieval food remains of the urban inhabitants.

**Tartu, St. Mary's Cemetery** is an urban burial site in southern Estonia, in the town of Tartu. The cemetery was used from the 2^nd^ half of the 13^th^ century to at least the beginning of the 18^th^ century (Malve et al., 2012, 147). The analysed chicken bone (aGal104, specimen ID TM-A-202/AZ-41:01) comes not directly from a burial context, but from a nearby deposit. According to the radiocarbon dating conducted in this study the chicken bone originates from 1176–1276 cal CE, i.e. from the Late Iron Age / Early Middle Ages (Fig. S1; Table S1).

**Tartu, Lossi 36–38** is an urban site in southern Estonia, in the town of Tartu. The three analysed chicken bones (aGal108, specimen ID TM-A-194/AZ-20:03; aGal111, specimen ID TM-A-194/AZ-21:04; aGal113, specimen ID TM-A-194/AZ-17:04) were radiocarbon dated in this study and originate from 1053–1264 cal CE, 1175–1272 cal CE and 1279–1393 cal CE, respectively (Fig. S1, Table S1). By associated finds, sample aGal113 comes from a medieval layer just on top of the Late Iron Age layer, which creates a possibility that this bone find could be associated either with Late Iron Age inhabitants or medieval inhabitants. The other two samples (aGal108 and aGal111) come from medieval layers and would thus most probably represent food remains of the urban inhabitants.

**Tartu, Lutsu 12** is an urban site in southern Estonia, in the town of Tartu. The analysed chicken bone (aGal114, specimen ID TM-A-244/AZ-4:16) comes from a medieval cesspit, from around the 2nd half of the 14th century (Tvauri et al., 2017, 150) and would thus be associated with medieval food remains of the urban inhabitants.

**Lohkva settlement** is located in southern Estonia and is dated to the 11th to 17th century (Roog & Malve 2013). The site was excavated prior to road construction works. The cultural layer had been mixed by ploughing. Pits that were found underneath this layer were archaeologically investigated. The analysed chicken specimens (aGal103, specimen ID TÜ-2004/AZ-10:08; aGal110, specimen ID TÜ-2004/AZ-14:02) were dated by associated finds to the early modern period, 16th to 17th century. By osteological and DNA analyses, the two specimens are most likely from the same individual, and thus, only aGal103 was included in the analysis (see Table S1).

**Lithuania**

**Klaipėda Castle** (previous Memelburg castle of the Teutonic order) is located in western Lithuania (55.7058368, 21.1267312; WGS 84). It was built between 1252 and 1253 by the Livonian Order and in 1328 was passed over to the Teutonic Order, which made it the Order’s northernmost castle in Prussia. In 1257–1258, Memelburg was granted Lübeck city rights, but at the end of the 13th century, further urban and economic development failed due to the wars with local Curonians, Samogitians, and Lithuanians (Žulkus, 2002). Excavations in 2016 in the northern part of the castle yielded 4,808 pieces of animal remains dating from the late 13th to the 18th century. Bird bones (NISP 81) accounted for 1.7% of the total zooarchaeological collection, while most of the bird remains (NISP 75) date back to the late 13th–early 14th century (Ehrlich et al., 2020). Two chicken bones (aGal22 and aGal23) dated to the late 15th–16th century were included in this study.

**Kernavė, medieval town** (54.8865149, 24.851869, WGS 84) was a large trade and craft centre and a strongly fortified residence of the Grand Duke of Lithuania between the 13^th^ and 14^th^ centuries. The castle and town were attacked by the Teutonic Order and burned down in 1390 and were never rebuilt. Thus, all the medieval remains of the city were left buried under a layer of fire, undisturbed by the events of later times (Luchtanas et al., 2002; Minkevičius et al., 2024). Over 1000 bird bones were collected during the excavations of 1999–2022 (Piličiauskienė et al., in prep.). For this study, 13 chicken bones (aGal31, aGal35-aGal38, aGal42-aGal44 and aGal46-aGal50) dated to the 13^th^–14^th^ century were included.

**Vilnius, Lower Castle** (54.68604541, 25.28801783; WGS 84) was the central castle of the Grand Duke in the capital of the Grand Duchy of Lithuania from the early 14^th^ to the mid-17^th^ century. It was first built as a part of Vilnius defensive system, and later, after several stages of reconstruction, it became the residential palace. It was abandoned after the Muscovian attack in the mid-17^th^ century and completely demolished at the beginning of the 19^th^ century CE. During the excavations of 1988–2015, a faunal collection of c. 100,000 specimens was collected. By 2020, 70,111 pieces of mammal, bird and fish remains, weighing 4,020 kg and dated from the 13^th^ to the 19^th^ century, had been examined. In total, 3672 (5.2%) bird bones were found and analysed (Rumbutis et al., 2018; Ehrlich et al., 2023), and eight chicken bones (aGal25-aGal29 and aGal32-aGal34) from the cultural layers dated to the 13^th^–16^th^ c. were included in this study.

**Trakai Peninsula castle** (54.646101, 24.937175; WGS 84) is one more castle, founded by the Grand Dukes of Lithuania. It was started to be built in the second half of the 14^th^ century. The castle was attacked by the Teutonic Order several times between 1377 and 1391. It was the residence of the Grand Duke of Lithuania in the first half of the 15^th^ century. From the end of the 16^th^ century to the first half of the 17^th^ century, it was the seat of the court of the Trakai voivodeship, while one of the towers served as a prison. In 1655, during the war with Moscow, the castle was badly damaged. In 1720, the castle was given to the Dominicans, who established a friary there. During the 2022 archaeological investigations, a total of 3,058 animal bone fragments, dated from the late 14^th^ to the early 16^th^ century, were collected. Bird bones accounted for 7.9% of the collection (Piličiauskienė et al., 2024). Two chicken bones (aGal24 and aGal30), dated to the 15^th^ century, were used in this study.

**Trakai Island Castle** is a medieval fortress located on an island in lake Galvė. Near the same lake, the Trakai Peninsula Castle was also built. The construction of Trakai Island Castle began in the second half of the 14^th^ century under Grand Duke of Lithuania Kęstutis and was completed in the early 15^th^ century by his son, Vytautas the Great. The castle is surrounded by water on all sides. It served as one of the main residences of the Grand Dukes of Lithuania and was an important political, military, and diplomatic centre of the Grand Duchy of Lithuania until the mid-16^th^ century. Between 1655 and 1660, the castle was destroyed during the war with Moscow and subsequently abandoned. It was rebuilt in the 20^th^ century. During archaeological excavations in 2021 (Budvydas, 2023), 1361 specimens of animal remains, dated to the late 14^th^–early 15^th^ century, were collected. Bird bones (n = 213) accounted for 7.9% of the entire zooarchaeological collection. One chicken bone (aGal45), dated to the late 14^th^–16^th^ century CE, was used in this study. The zooarchaeological collection from Trakai Island Castle is stored in the repository of the Faculty of History, Vilnius University.

Sampling permits for bone samples used in this study: MV/00514/2024, ÅLR 2023/7687, AI PP 749–750, AI PP 752, AI PP 755–756, TÜ PP 149–154, TÜ PP 157–161.

**References**

Appelgren, H. (1886). Muinaiskalupäiväkirja, no. 2498, Eurajoki. Unpublished finds catalogue. National Board of Antiquities.

Budvydas U. (2023). Trakų salos pilies tyrimai 2021-2022 m. Archeologiniai tyrinėjimai Lietuvoje 2023 metais. Vilnius: Lietuvos archeologijos draugija, 152–156. (only in Lithuanian)

Carlsson, R. (1987). Rapport. Arkeologisk undersökning. 12.23 Kastelholm; Kastelholms slott. KS 5, öster om Norra längan och Östra längans norra del. In Kastelholms slott. Arkeologiska undersökningar 1982 och 1983; KS1-KS14, Taktegelanalys. Museibyrån, Kastelholm 1988:1. Ålandstryckerier: Mariehamn 1988. 141–265.

Cederhvarf, B. (1904). Muinaiskalupäiväkirja, no. 4617, Jomala. Unpublished finds catalogue. National Board of Antiquities.

Cederhvarf, B. (1914). Muinaiskalupäiväkirja, no. 6769, Jomala. Unpublished finds catalogue. National Board of Antiquities.

Dreijer, M. (1958). Berättelse över utgrävningen av hustomtningarna 2 och 5 i Kohagen i Kvarnbo år 1958. Unpublished excavation report. Ålands museum.

Dreijer, S. (1961). Berättelse över en arkeologisk undersökning av ett 64 m² stort område strax väster om hustomting 5 på Kohagabacken i Kvarnbo, Saltvik, 1960. Unpublished excavation report. Ålands museum.

Edgren, T. (1996). Arkeologi i Hitis utskär. *Tidskriften skärgård* 3/1996, 15–21.

Ehrlich F., Piličiauskienė G., & Blaževičius P. (2023). Bird remains from Vilnius Lower Castle, Lithuania (13th–19th centuries) reveal changes in social status and unusual bird pathologies. *International Journal of Osteoarchaeology* 33(4): 731–741. <https://doi.org/10.1002/oa.3193>

Ehrlich F., Piličiauskienė G., Ubė Urbonaitė M., & Rannamäe E. (2020). The meaning of eagles in the Baltic Region. A case study from the castle of the Teutonic Order in Klaipėda, Lithuania (13th–14th Century). *Archaeologia Lituana* 21: 59-78. DOI: 10.15388/ArchLit.2019.21.4

Ehrlich, F., Rannamäe, E., Laneman, M., Tõrv, M., Lang, V., Oras, E., & Lõugas, L. (2021). In search of Estonia’s earliest chicken. Estonian Journal of Archaeology, 25(2), 160−181. <https://doi.org/10.3176/arch.2021.2.04>

Elfwendahl, M., & Åqvist, C. (1987). Rapport. Arkeologisk undersökning. 12.23 Kastelholm; Kastelholms slott. KS35, Stora borggården. In Kastelholms slott. Arkeologiska undersökningar 1985–1989; KS30-KS52, Bibliografi, Myntsammanställning, Osteologisk analys, 14C analys. Museibyrån, Kastelholm 1991:1. Ålandstryckerier: Mariehamn 1991. 203–218.

Elfwendahl (1988). Rapport. Arkeologisk undersökning. 12.23 Kastelholm; Kastelholms slott. KS8, Stora borggårdens sydvästra del. In Kastelholms slott. Arkeologiska undersökningar 1982 och 1983; KS1-KS14, Taktegelanalys. Museibyrån, Kastelholm 1988:1. Ålandstryckerier: Mariehamn 1988. 287–355.

Gustavsson, K. (1994). Gustavsson, K. (1994). Franciskanerklostret på Kökar. Nytt ljus över medeltiden i Skärgårdshavet. *Historisk Tidskrift för Finland*, *79*, 494-518.

Gustavsson, R. (2009). Rapport. Arkeologisk undersökning av sentida kulturlager vid fornlämning E 3.5, Eckerö Kyrkoby, vid schaktövervakning i samband med ombyggnad av landsväg 1. Museibyråns arkeologiska rapport 2009:3 Unpublished excavation report. Ålands museum.

Haak, A. (2004). Archaeological investigations at Viljandi castle of the Teutonic Order and in Medieval Viljandi. Archaeological Fieldwork in Estonia, 2003, 107–121.

Haak, A., & Valk, H. (2002). Archaeological investigations of medieval and post-medieval Viljandi. Archaeological Fieldwork in Estonia, 2001, 91–104.

Haak, A., Rannamäe, E., Luik, H., & Maldre, L. (2012). Worked and unworked bone from the Viljandi castle of the Livonian Order (13th–16th centuries). – Lietuvos archeologija, 38, 295–338.

Ilves, K. (2017). De arkeologiska undersökningarna vid Kvarnbohallen år 2016. Fornlämning Sa 14.9, fastighet Johannisberg 2:36. Kvarnbo, Saltvik, Åland. Unpublished excavation report. Ålands museum.

Kykyri, M. (2013). Kotka, Kotkansaari Ruukinkatu 15 tontti 285-1-7-5. Kaupunkiarkeologinen kaivaus 2.7.-31.7.2012. Unpublished excavation report. National Board of Antiquities.

Lang, V. (1996). Muistne Rävala: muistised, kronoloogia ja maaviljelusliku asustuse kujunemine LoodeEestis, eriti Pirita jõe alamjooksu piirkonnas. (Muinasaja teadus, 4.) Eesti Teaduste Akadeemia ajaloo instituut, Tallinn.

Lehtonen, H., & Aalto, I. (2015). Turku II/1/3 Rettingin tontti/Aboa Vetus-museon alue. Suuren kivitalon kellarin K94:9 arkeologinen kaivaus 2015. Kaivauskertomus. Unpublished excavation report. Aboa Vetus & Ars Nova Museum.

Luchtanas A., Vėlius G., & Bitner-Wróblewska A. (2002). *Kernavė-Litewska Troja*. Państwowe Muzeum Archeologiczne: Warszawa.

Malve, M., Roog, R., & Tvauri, A. (2012). Preliminary results of the rescue excavation in St Mary’s churchyard and its surroundings in Tartu 2010–2011. Archaeological Fieldwork in Estonia, 2011, 137–150.

Minkevičius K., Vengalis R., Piličiauskienė G., Poškienė J., Pilkauskas M., & Vėlius G. (2025). Agricultural development in the southeastern Baltic region from the late Bronze Age to the Medieval period: a case study of Kernavė, southeast Lithuania. *Vegetation History and Archaeobotany* 34, 349–362. <https://doi.org/10.1007/s00334-024-01016-5>

Núñez, M. (1973). Åland – Hammarland – Kattby. The possible remains of building foundations from the Viking Period. Unpublished excavation report. Ålands museum.

Pesonen, P. 2010. Hartola Uusi-Ruskeala C. Historiallisen ajan asuinpaikan kaivaus. Unpublished excavation report. National Board of Antiquities.

Piličiauskienė G., Minkevičius K., Šmigelskas R., & Micelicaitė V. (2024). Naujausi zooarcheologinės ir archeobotaninės medžiagos iš Trakų pusiasalio pilies tyrimai. *Prakalbinta priešistorė*, ed. G. Zabiela, Klaipėdos universiteto leidykla: Klaipėda, pp. 260-278.

Raninen, S. (2013). Pirkkala Tursiannotko ja Pirkkalankylä (Bircala). Arkeologinen pelastuskaivaus esihistoriallisella asuinpaikalla ja historiallisella kylätontilla 2012. Unpublished excavation report. National Board of Antiquities.

Rannamäe, E., Lõugas, L., Speller, C. F., Valk, H., Maldre, L., Wilczyński, J., Mikhailov, A., & Saarma, U. (2016). Three thousand years of continuity in the maternal lineages of ancient sheep (Ovis aries) in Estonia. PLoS ONE, 11: 10, e0163676. https://doi.org/10.1371/journal.pone.0163676

Rannamäe, E., Maldre, L., Ehrlich, F., Nuut, S., Lõugas, L., Haak, A., & Aguraiuja-Lätti, Ü. (2023). Dataset on zooarchaeological records of Estonian medieval and early modern mammal remains. https://doi.org/10.23673/re-443

Rinne, J. (1909). Muinaiskalupäiväkirja, no. 5455, Janakkala. Unpublished finds catalogue. National Board of Antiquities.

Roog, R. & Malve, M. (2013). Rescue excavations on the settlement site and rural cemetery of Lohkva, Tartumaa. Archaeological Fieldwork in Estonia, 2012, 241–250.

Rumbutis S., Blaževičius P., & Piličiauskienė G. (2018). Paukščiai Vilniaus pilyse. *Vilniaus pilių fauna: nuo kepsnio iki draugo*. Vilniaus universiteto leidykla: Vilnius, pp. 103–131.

Schultz, E-L., & Schultz, H-P. (1989). Hämeenlinna (58) Varikkoniemi. Kaivauskertomus 1986–1988. Unpublished excavation report. National Board of Antiquities.

Schultz, E-L., & Schultz, H-P. (1990). Hämeenlinna (121) Hattemala Tyrnoja. Kaivauskertomus 1989. Unpublished excavation report. National Board of Antiquities.

Suhonen, V-P. (2003). Vantaan Länsisalmen Gubbackan autiotontin arkeologiset tutkimukset vuonna 2003. Unpublished excavation report. National Board of Antiquities.

Tõnisson, E. (2008). Eesti muinaslinnad. Eds A. Mäesalu & H. Valk. (Muinasaja teadus, 20.) Tartu, Tallinn.

Tvauri, A. (2011) Archaeological investigations at the courtyard of Jakobi Street 2 / Lossi Street 3, Tartu. Archaeological Fieldwork in Estonia, 2010, 179–186.

Tvauri, A. (2012). The Migration Period, Pre-Viking Age, and Viking Age in Estonia. (Estonian Archaeology, 4). Tartu University Press, Tartu.

Tvauri, A., Bernotas, R., & Läänelaid, A. (2017). Archaeological excavations at the courtyard of Lutsu Street 12, Tartu. Archaeological Fieldwork in Estonia, 2016, 147–154.

Törnblom, L. (1980). Utgrävningsrapport från Kuretornet i huvudborgen. Unpublished excavation report. Ålands Museum.

Žulkus V. (2002). *Viduramžių Klaipėda*. Žara: Vilnius.

**
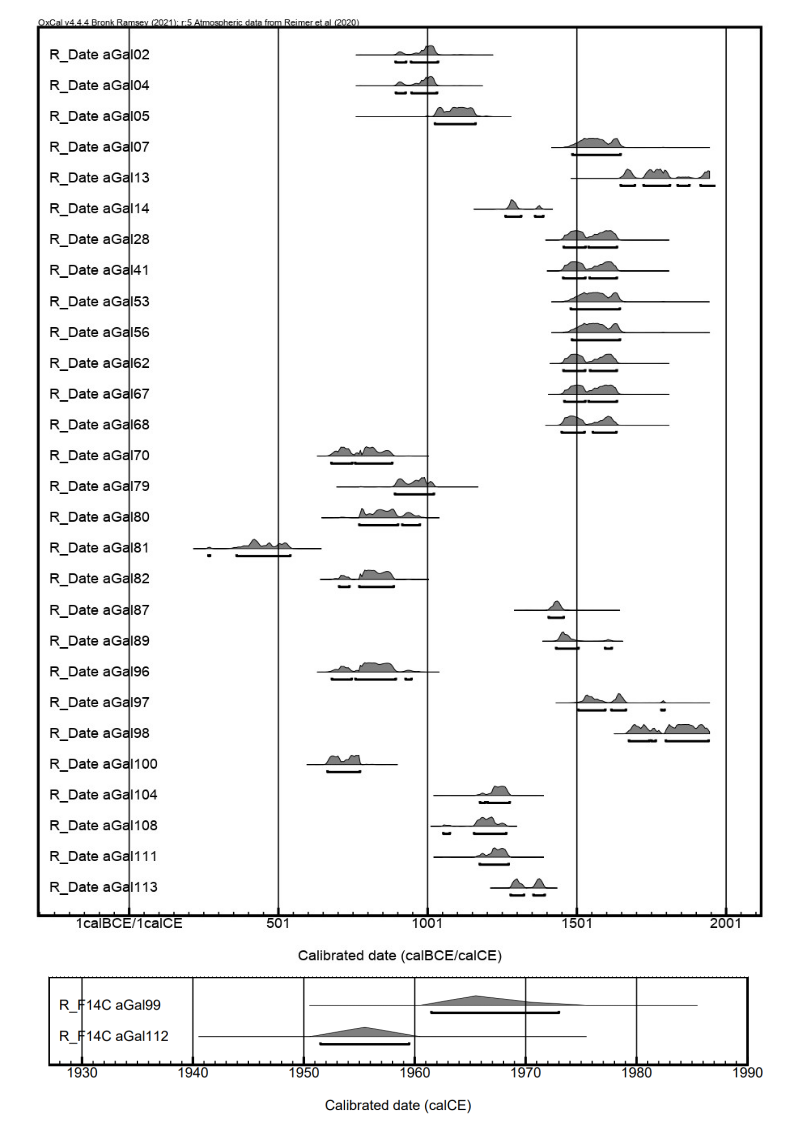
**

**Figure S1.** Calibrated ^14^C dates and Bayesian modelling of 28 bone collagen samples calibrated with the OxCal 4.4 program (Bronk Ramsey, 2009) with Bomb 13 NH1 (Hua et al., 2013) for aGal99 and aGal112 and IntCal20 (Reimer et al., 2020) for other Finnish, Estonian and Lithuanian chickens (*Gallus gallus domesticus*). Twenty-four of the samples were radiocarbon dated in this study, and for six samples published data were used. For details, see Table S1.

**References**

Bronk Ramsey, C. B. (2009). Bayesian analysis of radiocarbon dates. Radiocarbon, 51(1), 337–360. <https://doi.org/10.1017/s0033822200033865>

Hua, Q., Barbetti, M., & Rakowski, A. Z. (2013). Atmospheric radiocarbon for the period 1950–2010. *Radiocarbon*, *55*(4), 2059–2072. doi:10.2458/azu_js_rc.v55i2.16177

Reimer, P. J., Austin, W. E. N., Bard, E., Bayliss, A., Blackwell, P. G., Bronk Ramsey, C., Butzin, M., Cheng, H., Edwards, R. L., Friedrich, M., Grootes, P. M., Guilderson, T. P., Hajdas, I., Heaton, T. J., Hogg, A. G., Hughen, K. A., Kromer, B., Manning, S. W., Muscheler, R., … Talamo, S. (2020). The IntCal20 Northern Hemisphere radiocarbon age calibration curve (0-55 cal kBP). Radiocarbon, 62(4), 725–757. https://doi.org/10.1017/RDC.2020.41

**Table S2.** Primer pairs used for amplification of a 201-bp long fragment of mitochondrial control region, a 112 bp long fragment of *TSHR* gene (*thyroid-stimulating hormone receptor*) and a 105 bp long fragment of *BCDO2* gene (*β-carotene dioxygenase 2***)** in ancient and modern chicken (*Gallus gallus domesticus*) samples.

| **Primer pair** | **Forward primer 5’-3’** | **Reverse primer 5’-3’** | **Annealing temperature (°C)** | **Primer used for sequencing** | **Target region** | **Publication** |
| --- | --- | --- | --- | --- | --- | --- |
| GG144F/GG387R | ACCCATTATATGTATACGGGCATTAA | CGAGCATAACCAAATGGGTTAGA | 54 | F and R | D-loop | Storey et al. (2007) |
| Gal-TSHR-F/Gal-TSHR-R | GCTTTCTTCTTGCCCTTTTGC | ACAAAGACAACATAGGCTTCGG | 58 | R | SNP in *TSHR* linked to shifts in seasonal mating (missense mutation glycine to arginine in residue 558, chr5:40,089,599 G/A) | This study |
| Gal-BCDO2-F/Gal-BCDO2-R | GAGACCCTCTTACTCGGTGG | CCGCATTGTGGTCTCAGAA | 58 | F | SNP in *BCDO2* linked to yellow skin phenotype (chr24:6,273,428 A/G) | This study |

**References**

Storey, A. A., Ramírez, J. M., Quiroz, D., Burley, D. V., Addison, D. J., Walter, R., Anderson, A. J., Hunt, T. L., Athens, J. S., Huynen, L., & Matisoo-Smith, E. A. (2007). Radiocarbon and DNA evidence for a pre-Columbian introduction of Polynesian chickens to Chile. *Proceedings of the National Academy of Sciences USA*, *104(25),* 10335–10339. <https://doi.org/10.1073/pnas.0703993104>


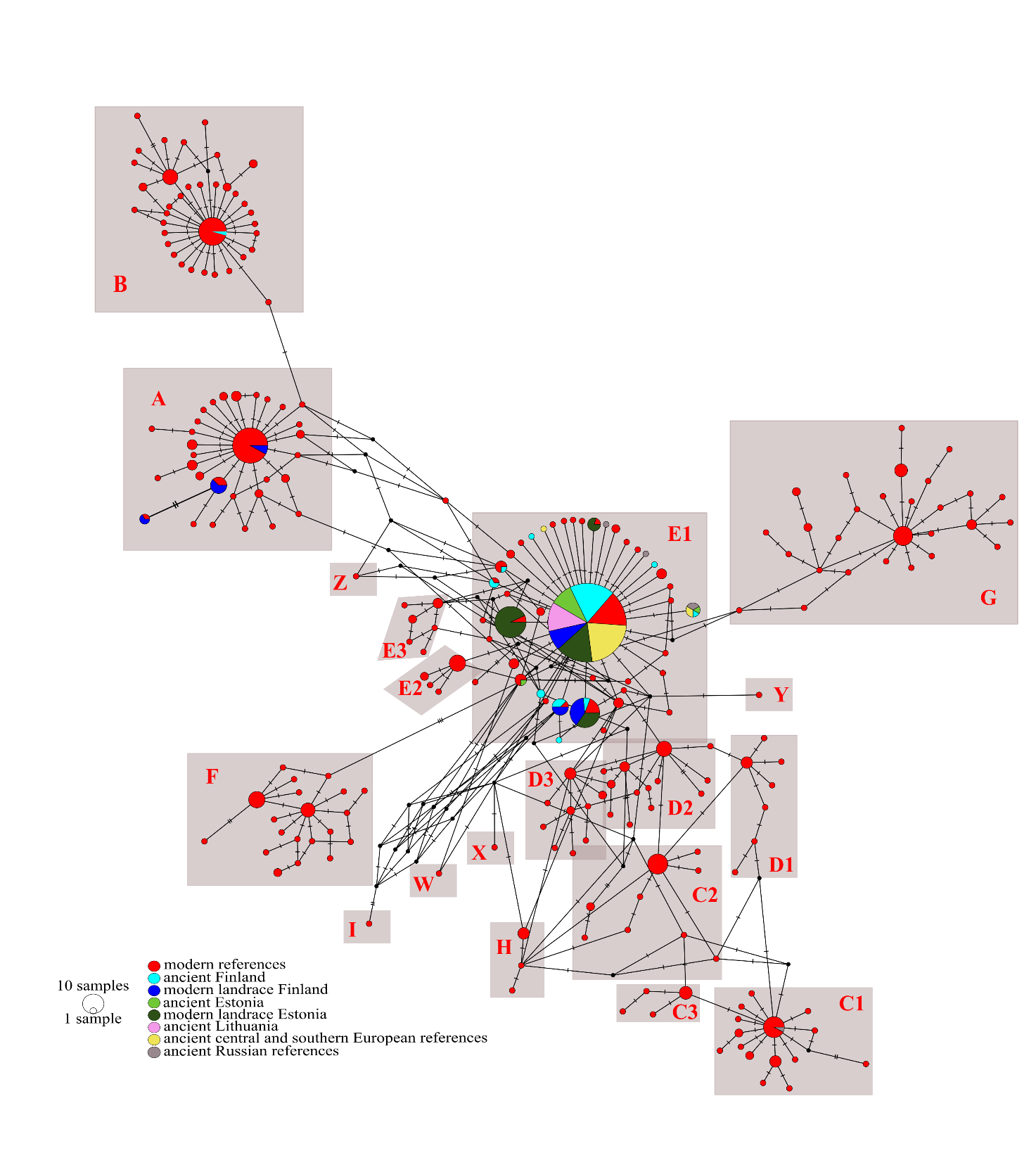


**Figure S2.** Median-joining haplotype network constructed from a 201-bp long mitochondrial control region fragment of ancient and modern landrace chicken (*Gallus gallus domesticus*) samples, as well as reference sequences from central and southern European and western Russian ancient chickens and modern chicken breeds. The size of the circles is proportional to the number of samples having that haplotype. The bolded letters and boxes represent haplogroups. Tick marks represent mutational differences between haplotypes.


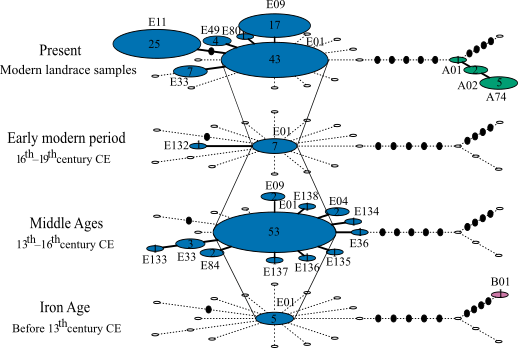


**Figure S3.** Temporal statistical parsimony network of the 201-bp long mitochondrial control region fragment of ancient Finnish, Estonian and Lithuanian samples, as well as modern Finnish and Estonian landrace chicken (*Gallus gallus domesticus*) samples. The size of the ellipses is proportional to the frequency of each haplotype, and the number of individuals is indicated with a number within the ellipse. Small white ellipses indicate haplotypes absent in that period. The number of black dots +1 connecting the haplotypes equals to the number of nucleotide differences between haplotypes.
