# Supplementary material for: Ancient DNA unravels the history of chickens in the Baltic Sea region and the continuity of landrace lineages
Source: Heredity (Edinb). 2026 Apr 13;135(6):397–408. doi: 10.1038/s41437-026-00842-9 (PMC13354571; doi:10.1038/s41437-026-00842-9)
Supplement: Supplementary file 2 — Supplementary information [file 41437_2026_842_MOESM2_ESM.pdf]

**Table S1.** Details of ancient chicken (*Gallus gallus domesticus* ) samples (aGal) and modern landrace chicken samples (GG) used in this study. Skeletal element, archaeological dating, radiocarbon dating and calibrated radiocarbon dating information are only filled for the ancient samples. (*) indicates new haplotype names given by us for haplotypes that belong to haplogroup E1 but are not among the reference sequences. For *TSHR* (*thyroid-stimulating hormone receptor* ), Gly means wild-type allele and Arg means sweep allele. For *BCDO2* (*β-carotene dioxygenase 2* ), Y means the recessive yellow skin colour-causing allele, and W means the dominant white/grey skin colour-causing allele. (x1) means that the SNP was sequenced only from one independent reaction from the ancient sample. (-) means that no result for this genetic region was obtained due to no amplification of the region. To have a wide time range of samples, radiocarbon dating was performed for samples selected based on the archaeological stratigraphy (i.e. those with ambiguous context). Previously radiocarbon dated samples have been re-calibrated in this study.

| Sample ID | Country/Sampling site of ancient sample or lineage of modern landrace sample | Sampling site number in Fig 1 | Skeletal element | Archaeological dating | Radiocarbon dating | Calibrated radiocarbon dating (95.4%) | mtDNA haplo-type | *TSHR* | *BCDO2* |
|---|---|---|---|---|---|---|---|---|---|
| aGal04 | Finland (mainland)/Pirkkala, Tursiannotko | 1 | Tarsometarsus | mid-10th century CE | 1057±32 BP | 894–1032 cal CE | E01 | Arg/Arg (x1) | Y/Y (x1) |
| aGal05 | Finland (mainland)/Pirkkala, Tursiannotko | 1 | Tarsometarsus | mid-10th century CE | 957±32 BP | 1025–1161 cal CE | E01 | Arg/Arg (x1) | Y/Y |
| aGal02 | Finland (mainland)/Hämeenlinna, Varikkoniemi | 2 | Tarsometarsus | 9th–14th century CE | 1051±33 BP | 893–1036 cal CE | - | - | - |
| aGal39 | Finland (mainland)/Hämeenlinna, Hattelmala | 2 | femur | 6th century BCE–12th century CE | | | - | - | - |
| aGal12 | Finland (mainland)/Janakkala, Hakoinen Castle | 3 | tibiotarsus | 14th–15th century CE | | | - | - | - |
| aGal18 | Finland (mainland)/Janakkala, Hakoinen Castle | 3 | femur | 14th–15th century CE | | | - | - | - |
| aGal21 | Finland (mainland)/Janakkala, Hakoinen Castle | 3 | radius | 14th–15th century CE | | | - | - | - |
| aGal72 | Finland (mainland)/Janakkala, Hakoinen Castle | 3 | radius | 13th–14th century CE | | | E137* | - | - |
| aGal40 | Finland (mainland)/Hartola, Uusi-Ruskeala | 4 | femur | 11th – 18th century CE | | | - | - | - |
| aGal09 | Finland (mainland)/Eurakoski, Eurajoki Liinmaa Castle | 5 | cranium | 14th–15th century CE | | | - | - | - |
| aGal10 | Finland (mainland)/Eurakoski, Eurajoki Liinmaa Castle | 5 | Tarsometarsus | 14th–15th century CE | | | - | - | - |
| aGal11 | Finland (mainland)/Eurakoski, Eurajoki Liinmaa Castle | 5 | cranium | 14th–15th century CE | | | - | - | - |
| aGal06 | Finland (mainland)/Turku, Aboa Vetus | 6 | tibiotarsus | 14th–15th century CE | | | E33 | - | - |
| aGal07 | Finland (mainland)/Turku, Aboa Vetus | 6 | tibiotarsus | 14th–15th century CE | 315±32 BP | 1486–1647 cal CE | E33 | Arg/Gly | - |
| aGal08 | Finland (mainland)/Turku, Aboa Vetus | 6 | tibiotarsus | 14th–15th century CE | | | - | - | - |
| aGal15 | Finland (mainland)/Hiittinen, Högholmen | 7 | humerus | 14th–15th century CE | | | - | - | - |
| aGal65 | Finland (mainland)/Hiittinen, Högholmen | 7 | humerus | 13th–14th century CE | | | - | - | - |
| aGal13 | Finland (mainland)/Raasepori, Junkarsborg | 8 | femur | 14th–15th century CE | 191±31 BP | 1648–1950 cal CE | E01 | - | W/W |
| aGal14 | Finland (mainland)/Raasepori, Junkarsborg | 8 | femur | 14th–15th century CE | 709±31 BP | 1262–1388 cal CE | E01 | Arg/Gly | Y/Y |

| | | | | | | | | | | |
|---|---|---|---|---|---|---|---|---|---|---|
| aGal16 | Finland (mainland)/Raasepori, Junkarsborg | 8 | tibiotarsus | 14th–15th century CE | | | - | - | - |
| aGal17 | Finland (mainland)/Raasepori, Junkarsborg | 8 | tibiotarsus | 14th–15th century CE | | | E133* | - | - |
| aGal19 | Finland (mainland)/Raasepori, Junkarsborg | 8 | femur | 14th–15th century CE | | | E01 | - | - |
| aGal20 | Finland (mainland)/Raasepori, Junkarsborg | 8 | femur | 14th–15th century CE | | | - | - | - |
| aGal41 | Finland (mainland)/Vantaa, Gubbacka | 9 | humerus | 15th – 17th century CE | 362±31 BP | 1455–1635 cal CE | E01 | Gly/Gly | - |
| aGal01 | Finland (mainland)/Kotka, Kotkansaari | 10 | Tarsometarsus | late 18th– mid-19th century CE | | | E01 | - | - |
| aGal03 | Finland (mainland)/Kotka, Kotkansaari | 10 | Tarsometarsus | late 18th– mid-19th century CE | | | E132* | - | - |
| aGal82 | Finland (Åland Islands)/Saltvik, Kvarnbo Kohagen | 11 | carpometacarpus | mid-6th–14th century CE | 1216±27 BP | 704–887 cal CE | E01 | Arg/Gly | W/Y |
| aGal83 | Finland (Åland Islands)/Saltvik, Kvarnbo Kohagen | 11 | radius | mid-6th–14th century CE | | | E01 | - | W/W |
| aGal84 | Finland (Åland Islands)/Saltvik, Kvarnbo Kohagen | 11 | carpometacarpus | mid-6th–14th century CE | | | - | - | - |
| aGal81 | Finland (Åland Islands)/Saltvik, Kvarnbo | 12 | scapula | 7th–mid-11th century CE | 1638±34 BP | 266–541 cal CE | B01 | Gly/Gly | Y/Y |
| aGal86 | Finland (Åland Islands)/Eckerö, Thors | 13 | ulna | 17th–19th century CE | | | - | - | - |
| aGal94 | Finland (Åland Islands)/Eckerö, Thors | 13 | ulna | 17th–19th century CE | | | - | - | - |
| aGal70 | Finland (Åland Islands)/Hammarland, Kattby | 14 | tibiotarsus | 7th–mid-11th century CE | 1238±32 BP | 678–882 cal CE | E01 | Arg/Arg | W/W |
| aGal79 | Finland (Åland Islands)/Jomala, Brömsängsbacken | 15 | tibiotarsus | 10th–mid-11th century CE | 1085±32 BP | 891–1022 cal CE | - | - | - |
| aGal80 | Finland (Åland Islands)/Jomala, Brömsängsbacken | 15 | tibiotarsus | 9th–mid-11th century CE | 1174±32 BP | 772–975 cal CE | - | - | - |
| aGal51 | Finland (Åland Islands)/Sund, Kastelholm Castle | 16 | tibiotarsus | early to mid-15th century CE | | | E01 | Gly/Gly | W/W |
| aGal52 | Finland (Åland Islands)/Sund, Kastelholm Castle | 16 | tibiotarsus | early to mid-15th century CE | | | E01 | Gly/Gly | W/W |
| aGal53 | Finland (Åland Islands)/Sund, Kastelholm Castle | 16 | tibiotarsus | mid to late 16th century CE | 320±32 BP | 1480–1645 cal CE | E84 | Arg/Gly | W/Y |
| aGal54 | Finland (Åland Islands)/Sund, Kastelholm Castle | 16 | tibiotarsus | early to mid-15th century CE | | | E36 | Gly/Gly | W/W |
| aGal55 | Finland (Åland Islands)/Sund, Kastelholm Castle | 16 | tibiotarsus | 17th–18th century CE | | | E01 | Arg/Gly | W/W |
| aGal56 | Finland (Åland Islands)/Sund, Kastelholm Castle | 16 | tibiotarsus | mid to late 16th century CE | 318±32 BP | 1484–1645 cal CE | E01 | Gly/Gly | W/W |
| aGal58 | Finland (Åland Islands)/Sund, Kastelholm Castle | 16 | tibiotarsus | mid to late 15th century CE | | | E135* | Gly/Gly(x1) | Y/Y |
| aGal59 | Finland (Åland Islands)/Sund, Kastelholm Castle | 16 | tibiotarsus | mid to late 15th century CE | | | E01 | Gly/Gly | W/Y |

| | | | | | | | | | |
|---|---|---|---|---|---|---|---|---|---|
| aGal61 | Finland (Åland Islands)/Sund, Kastelholm Castle | 16 | tibiotarsus | mid to late 16th century CE | | | E01 | Arg/Arg | W/W |
| aGal62 | Finland (Åland Islands)/Sund, Kastelholm Castle | 16 | tibiotarsus | 17th–18th century CE | 361±28 BP | 1456–1635 cal CE | E04 | Gly/Gly | W/W |
| aGal63 | Finland (Åland Islands)/Sund, Kastelholm Castle | 16 | tibiotarsus | mid to late 16th century CE | | | E04 | Gly/Gly | W/W |
| aGal64 | Finland (Åland Islands)/Sund, Kastelholm Castle | 16 | tibiotarsus | early to mid-16th century CE | | | - | - | - |
| aGal66 | Finland (Åland Islands)/Sund, Kastelholm Castle | 16 | tibiotarsus | mid to late 15th century CE | | | E136* | - | - |
| aGal67 | Finland (Åland Islands)/Sund, Kastelholm Castle | 16 | tibiotarsus | early to mid-15th century CE | 353±31 BP | 1459–1635 cal CE | E84 | Gly/Gly | W/W |
| aGal68 | Finland (Åland Islands)/Sund, Kastelholm Castle | 16 | tibiotarsus | 17th–18th century CE | 373±31 BP | 1450–1633 cal CE | E33 | Gly/Gly | Y/Y |
| aGal71 | Finland (Åland Islands)/Sund, Kastelholm Castle | 16 | radius | 13th–14th century CE | | | E01 | Gly/Gly | W/W |
| aGal73 | Finland (Åland Islands)/Sund, Kastelholm Castle | 16 | tibiotarsus | mid-11th–19th century CE | | | E01 | Gly/Gly | Y/Y |
| aGal74 | Finland (Åland Islands)/Sund, Kastelholm Castle | 16 | tibiotarsus | 17th–18th century CE | | | E01 | - | - |
| aGal75 | Finland (Åland Islands)/Sund, Kastelholm Castle | 16 | tibiotarsus | mid to late 15th century CE | | | E01 | Arg/Arg (x1) | W/W |
| aGal76 | Finland (Åland Islands)/Sund, Kastelholm Castle | 16 | tibiotarsus | 15th–16th century CE | | | - | - | - |
| aGal77 | Finland (Åland Islands)/Sund, Kastelholm Castle | 16 | tibiotarsus | early to mid-15th century CE | | | E01 | - | - |
| aGal85 | Finland (Åland Islands)/Sund, Kastelholm Castle | 16 | radius | 13th–14th century CE | | | E09 | Gly/Gly | Y/Y |
| aGal88 | Finland (Åland Islands)/Sund, Kastelholm Castle | 16 | tibiotarsus | early to mid-15th century CE | | | E01 | - | - |
| aGal90 | Finland (Åland Islands)/Sund, Kastelholm Castle | 16 | tibiotarsus | mid to late 16th century CE | | | E01 | Gly/Gly | Y/Y |
| aGal91 | Finland (Åland Islands)/Sund, Kastelholm Castle | 16 | tibiotarsus | early to mid-16th century CE | | | E01 | Arg/Gly | W/W |
| aGal92 | Finland (Åland Islands)/Sund, Kastelholm Castle | 16 | tibiotarsus | early to mid-16th century CE | | | E01 | Gly/Gly | Y/Y |
| aGal93 | Finland (Åland Islands)/Sund, Kastelholm Castle | 16 | synsacrum | 13th–14th century CE | | | E01 | Gly/Gly | W/W (x1) |
| aGal95 | Finland (Åland Islands)/Sund, Kastelholm Castle | 16 | radius | 13th–14th century CE | | | E01 | Gly/Gly | W/Y |
| aGal57 | Finland (Åland Islands)/Kökar Friary/Presbytery | 17 | femur | 12th–16th century CE | | | - | - | - |
| aGal60 | Finland (Åland Islands)/Kökar Friary/Presbytery | 17 | femur | 16th–19th century CE | | | E01 | Arg/Gly | Y/Y |
| aGal69 | Finland (Åland Islands)/Kökar Friary/Presbytery | 17 | femur | 12th–16th century CE | | | E01 | - | - |
| aGal78 | Finland (Åland Islands)/Kökar Friary/Presbytery | 17 | femur | 12th–16th century CE | | | E01 | - | - |

| | | | | | | | | | |
|---|---|---|---|---|---|---|---|---|---|
| aGal87 | Finland (Åland Islands)/Kökar Friary/Presbytery | 17 | femur | 12th–16th century CE | 480±31 BP | 1406–1457 cal CE | E09 | Arg/Gly | W/W |
| aGal89 | Finland (Åland Islands)/Kökar Friary/Presbytery | 17 | femur | 12th–19th century CE | 418±27 BP | 1431–1618 cal CE | E01 | Gly/Gly | - |
| aGal100 | Estonia/Ilmandu III *tarand* -grave | 18 | tibiotarsus | 8th–4th century BCE | 1293±24 BP (Ehrlich et al., 2021) | 665–774 cal CE | - | - | - |
| aGal96 | Estonia/Iru fortified settlement/hillfort | 19 | femur | 9th–6th century BCE | 1213±39 BP (Ehrlich et al., 2021) | 680–947 cal CE | E01 | Arg/Gly | Y/Y |
| aGal99 | Estonia/Jõelähtme stone-cist cemetery | 20 | humerus | 11th–9th century BCE | 162.17, 0.4 pMC (Ehrlich et al., 2021) | 1961–1973 cal CE | E01 | Gly/Gly | Y/Y |
| aGal98 | Estonia/Joaorg at Narva settlement/hilltop site | 21 | tibiotarsus | from the Mesolithic to the Modern Period | 130±30 BP (Ehrlich et al., 2021) | 1675–1942 cal CE | E01 | Gly/Gly(x1) | Y/Y |
| aGal112 | Estonia (Saaremaa)/Kurevere stone grave | 22 | tibiotarsus | 10th–11th century CE | 102.87, 0.32 pMC (Ehrlich et al., 2021) | 1951–1959 cal CE | E01 | Gly/Gly | Y/Y |
| aGal97 | Estonia (Saaremaa)/Loona settlement | 23 | tibiotarsus | 18th–13th century BCE | 280±30 BP (Ehrlich et al., 2021) | 1505–1795 cal CE | E01 | Gly/Gly(x1) | Y/Y (x1) |
| aGal109 | Estonia/Pärnu, Põhja St | 24 | femur | 17th–18th century CE | | | E01 | Gly/Gly | Y/Y |
| aGal101 | Estonia/Viljandi Castle | 25 | ulna | 14th–15th century CE | | | E01 | Gly/Gly | W/Y |
| aGal106 | Estonia/Viljandi Castle | 25 | humerus | 13th–16th century CE | | | E01 | Gly/Gly | Y/Y |
| aGal105 | Estonia/Viljandi, Pikk 4 | 25 | humerus | mid-13th–early 14th century CE | | | E138* | - | Y/Y |
| aGal107 | Estonia/Viljandi, Laidoneri väljak 10 | 25 | tibiotarsus | 2nd half of 13th–early 15th century CE | | | E01 | Gly/Gly | Y/Y |
| aGal102 | Estonia/Tartu, Jakobi 2 | 26 | tibiotarsus | 13th–14th century CE | | | E01 | - | - |
| aGal104 | Estonia/Tartu St. Mary's Cemetery | 26 | humerus | 13th–18th century CE | 812±30 BP | 1176–1276 cal CE | E01 | Gly/Gly | Y/Y |
| aGal108 | Estonia/Tartu, Lossi 36–38 | 26 | femur | 13th–16th century CE | 855±30 BP | 1053–1264 cal CE | E01 | - | W/Y |
| aGal111 | Estonia/Tartu, Lossi 36–38 | 26 | femur | 13th–16th century CE | 821±30 BP | 1175–1272 cal CE | E01 | Gly/Gly | W/Y |
| aGal113 | Estonia/Tartu, Lossi 36-38 | 26 | humerus | 13th–16th century CE | 664±29 BP | 1279–1393 cal CE | E08 | Gly/Gly(x1) | - |
| aGal114 | Estonia/Tartu, Lutsu 12 | 26 | tibiotarsus | 2nd half of the 14th century CE | | | E01 | Gly/Gly | W/Y |
| aGal103 | Estonia/Lohkva settlement | 27 | tibiotarsus | 16th–17th century CE | | | E01 | Gly/Gly | Y/Y |
| aGal22 | Lithuania/Klaipėda, (Memelburg), Castle of Teutonic Order | 28 | humerus | late 15th–16th century CE | | | E01 | - | - |
| aGal23 | Lithuania/Klaipėda, (Memelburg), Castle of Teutonic Order | 28 | humerus | late 15th–16th century CE | | | E01 | Gly/Gly | Y/Y |
| aGal31 | Lithuania/Kernave, Aukuras hill, Hillfort | 29 | humerus | 13th–14th century CE | | | E01 | Gly/Gly | Y/Y |
| aGal35 | Lithuania/Kernave, Aukuras hill, Hillfort | 29 | humerus | 13th–14th century CE | | | E01 | Gly/Gly | Y/Y |

| | | | | | | | | | |
|---|---|---|---|---|---|---|---|---|---|
| aGal36 | Lithuania/Kernave, Aukuras hill, Hilfort | 29 | tibiotarsus | 13th–14th century CE | | | E01 | Gly/Gly | Y/Y (x1) |
| aGal37 | Lithuania/Kernave, Aukuras hill, Hillfort | 29 | carpometacarpus | 13th–14th century CE | | | - | - | - |
| aGal38 | Lithuania/Kernave, Aukuras hill, Hilfort | 29 | humerus | 13th–14th century CE | | | E01 | Arg/Arg (x1) | Y/Y (x1) |
| aGal42 | Lithuania/Kernave, Aukuras hill, Hillfort | 29 | femur | 13th–14th century CE | | | E01 | Gly/Gly | W/W |
| aGal43 | Lithuania/Kernave, Aukuras hill, Hilfort | 29 | humerus | 13th–14th century CE | | | E01 | - | - |
| aGal44 | Lithuania/Kernave, Aukuras hill, Hillfort | 29 | femur | 13th–14th century CE | | | E01 | Gly/Gly | Y/Y |
| aGal46 | Lithuania/Kernave, Aukuras hill, Hilfort | 29 | sternum | 13th–14th century CE | | | E01 | - | - |
| aGal47 | Lithuania/Kernave, Aukuras hill, Hillfort | 29 | humerus | 13th–14th century CE | | | E01 | Gly/Gly | Y/Y |
| aGal48 | Lithuania/Kernave, Aukuras hill, Hilfort | 29 | tibiotarsus | 13th–14th century CE | | | E01 | Gly/Gly | Y/Y |
| aGal49 | Lithuania/Kernave, Aukuras hill, Hillfort | 29 | humerus | 13th–14th century CE | | | E134* | Gly/Gly | Y/Y |
| aGal50 | Lithuania/Kernave, Aukuras hill, Hilfort | 29 | radius | 13th–14th century CE | | | - | - | - |
| aGal25 | Lithuania/Vilnius, Lower Castle | 30 | humerus | 16th century CE | | | E01 | Gly/Gly | Y/Y |
| aGal26 | Lithuania/Vilnius, Lower Castle | 30 | humerus | 16th century CE | | | E01 | Gly/Gly | Y/Y |
| aGal27 | Lithuania/Vilnius, Lower Castle | 30 | tibiotarsus | late 14th–15th century CE | | | E01 | Gly/Gly | W/W |
| aGal28 | Lithuania/Vilnius, Lower Castle | 30 | tibiotarsus | 13th–mid-14th century CE | 357±32 BP | 1457–1635 cal CE | E01 | Gly/Gly | Y/Y |
| aGal29 | Lithuania/Vilnius, Lower Castle | 30 | tibiotarsus | 15th century CE | | | E01 | Gly/Gly | Y/Y |
| aGal32 | Lithuania/Vilnius, Lower Castle | 30 | tibiotarsus | late 14th–15th century CE | | | E01 | Gly/Gly | Y/Y |
| aGal33 | Lithuania/Vilnius, Lower Castle | 30 | tibiotarsus | late 14th–15th century CE | | | E01 | Gly/Gly | Y/Y |
| aGal34 | Lithuania/Vilnius, Lower Castle | 30 | tibiotarsus | late 14th–15th century CE | | | E01 | Gly/Gly | W/Y |
| aGal24 | Lithuania/Trakai, Peninsula castle | 31 | humerus | 15th century CE | | | E01 | Gly/Gly | Y/Y |
| aGal30 | Lithuania/Trakai, Peninsula castle | 31 | humerus | 15th century CE | | | E01 | Gly/Gly | Y/Y |
| aGal45 | Lithuania/Trakai, Island Castle | 31 | synsacrum | late 14th–16th century CE | | | - | - | - |
| GG01 | Finland/Piikkiö | | | | | | E01 | Gly/Gly | W/W |
| GG02 | Finland/Piikkiö | | | | | | A02 | Gly/Gly | W/W |

| | | | | |
|---|---|---|---|---|
| GG03 | Finland/Häme | E01 | Gly/Gly | W/Y |
| GG04 | Finland/Häme | E01 | Gly/Gly | Y/Y |
| GG05 | Finland/Häme | E01 | Gly/Gly | Y/Y |
| GG06 | Finland/Jussila | E33 | Gly/Gly | Y/Y |
| GG07 | Finland/Jussila | E33 | Gly/Gly | Y/Y |
| GG08 | Finland/Jussila | E33 | Gly/Gly | Y/Y |
| GG09 | Finland/Jussila | E33 | Gly/Gly | Y/Y |
| GG10 | Finland/Ilmajoki | E09 | Gly/Gly | Y/Y |
| GG11 | Finland/Ilmajoki | A02 | Gly/Gly | Y/Y |
| GG12 | Finland/Ilmajoki | E09 | Gly/Gly | Y/Y |
| GG13 | Finland/Ilmajoki | E09 | Gly/Gly | Y/Y |
| GG14 | Finland/Hornio | E01 | Gly/Gly | Y/Y |
| GG15 | Finland/Hornio | E01 | Gly/Gly | Y/Y |
| GG16 | Finland/Hornio | E01 | Gly/Gly | Y/Y |
| GG17 | Finland/Kiuruvesi | E01 | Gly/Gly | W/Y |
| GG18 | Finland/Kiuruvesi | E01 | Gly/Gly | W/W |
| GG19 | Finland/Kiuruvesi | E01 | Gly/Gly | W/Y |
| GG20 | Finland/Kiuruvesi | A74 | Gly/Gly | Y/Y |
| GG21 | Finland/Kiuruvesi | A74 | Arg/Gly | Y/Y |
| GG22 | Finland/Savitaipale | E01 | Gly/Gly | Y/Y |
| GG23 | Finland/Savitaipale | A01 | Gly/Gly | Y/Y |
| GG24 | Finland/Savitaipale | A01 | Gly/Gly | Y/Y |
| GG25 | Finland/Alho | A74 | Gly/Gly | W/W |
| GG26 | Finland/Alho | A74 | Arg/Gly | W/W |

| | | | | |
|---|---|---|---|---|
| GG27 | Finland/Alho | A74 | Arg/Gly | W/W |
| GG28 | Finland/Tyrnävä | E01 | Gly/Gly | W/W |
| GG29 | Finland/Tyrnävä | E09 | Gly/Gly | Y/Y |
| GG30 | Finland/Tyrnävä | E01 | Gly/Gly | W/Y |
| GG31 | Finland/Tyrnävä | E09 | Gly/Gly | Y/Y |
| GG32 | Finland/Tyrnävä | E09 | Gly/Gly | Y/Y |
| GG33 | Finland/Tyrnävä | E09 | Gly/Gly | Y/Y |
| GG34 | Finland/Tyrnävä | E09 | Gly/Gly | Y/Y |
| GG35 | Finland/Tyrnävä | E09 | Gly/Gly | Y/Y |
| GG36 | Finland/Tyrnävä | E09 | Gly/Gly | Y/Y |
| GG37 | Finland/Iiti | E01 | Gly/Gly | W/W |
| GG38 | Finland/Iiti | E01 | Gly/Gly | W/W |
| GG39 | Finland/Iiti | E01 | Gly/Gly | Y/Y |
| GG40 | Estonia/Liivi | E01 | Gly/Gly | Y/Y |
| GG41 | Estonia/Liivi | E49 | Gly/Gly | Y/Y |
| GG42 | Estonia/Liivi | E49 | Gly/Gly | Y/Y |
| GG43 | Estonia/mix of unknown landrace lineages | E01 | Gly/Gly | Y/Y |
| GG44 | Estonia/mix of unknown landrace lineages | E09 | Gly/Gly | Y/Y |
| GG45 | Estonia/mix of unknown landrace lineages | E01 | Gly/Gly | W/W |
| GG46 | Estonia/Lõo | E11 | Gly/Gly | Y/Y |
| GG47 | Estonia/Liivi | E49 | Gly/Gly | Y/Y |
| GG48 | Estonia/Liivi | E49 | Gly/Gly | Y/Y |
| GG49 | Estonia/mix of Lõo, Viru and Võru | E11 | Gly/Gly | W/W |
| GG50 | Estonia/landrace chicken from Põvvatu village | E09 | Gly/Gly | Y/Y |

| | | | | |
|---|---|---|---|---|
| GG51 | Estonia/mix of Vormsi and Keedika | E11 | Arg/Arg | Y/Y |
| GG52 | Estonia/mix of Vormsi and Keedika | E11 | Arg/Arg | Y/Y |
| GG53 | Estonia/mix of Härmä, Vormsi and Keedika | E11 | Gly/Gly | Y/Y |
| GG54 | Estonia/mix of Härmä, Vormsi and Keedika or mix of Vormsi and Keedika | E01 | Arg/Gly | Y/Y |
| GG55 | Estonia/mix of Härmä, Vormsi and Keedika or mix of Vormsi and Keedika | E11 | Gly/Gly | Y/Y |
| GG56 | Estonia/mix of Vormsi and Keedika | E01 | Gly/Gly | Y/Y |
| GG57 | Estonia/mix of Vormsi and Keedika | E11 | Arg/Gly | Y/Y |
| GG58 | Estonia/mix of Vormsi and Keedika | E01 | Arg/Gly | Y/Y |
| GG59 | Estonia/mix of Vormsi, Keedika and Liivi | E09 | Arg/Gly | Y/Y |
| GG60 | Estonia/mix of Vormsi, Keedika and Liivi | E01 | Gly/Gly | Y/Y |
| GG61 | Estonia/mix of Härma, Vormsi, Keedika and Liivi | E09 | Arg/Gly | Y/Y |
| GG62 | Estonia/mix of Vormsi and Keedika | E09 | Gly/Gly | Y/Y |
| GG63 | Estonia/mix of Vormsi and Keedika | E01 | Gly/Gly | Y/Y |
| GG64 | Estonia/mix of Vormsi and Keedika | E01 | Gly/Gly | Y/Y |
| GG65 | Estonia/mix of Vormsi and Keedika | E01 | Arg/Gly | Y/Y |
| GG66 | Estonia/mix of Vormsi and Keedika | E11 | Arg/Arg | Y/Y |
| GG67 | Estonia/mix of Vormsi and Keedika | E01 | Gly/Gly | Y/Y |
| GG68 | Estonia/mix of Vormsi and Keedika | E01 | Arg/Gly | Y/Y |
| GG69 | Estonia/mix of Vormsi and Keedika | E01 | Gly/Gly | Y/Y |
| GG70 | Estonia/mix of Vormsi and Keedika | E11 | Arg/Arg | Y/Y |
| GG71 | Estonia/mix of Vormsi and Keedika | E01 | Arg/Gly | Y/Y |
| GG72 | Estonia/mix of Vormsi and Keedika | E11 | Arg/Arg | Y/Y |
| GG73 | Estonia/mix of Vormsi and Keedika | E01 | Arg/Arg | Y/Y |
| GG74 | Estonia/Viru | E01 | Gly/Gly | W/W |

| | | | | |
|---|---|---|---|---|
| GG75 | Estonia/Võru | E01 | Gly/Gly | W/W |
| GG76 | Estonia/Lõo | E11 | Gly/Gly | W/W |
| GG77 | Estonia/mix of Vormsi and Keedika | E01 | Arg/Gly | W/Y |
| GG78 | Estonia/mix of Vormsi, Keedika, Lõo and Liivi | E80 | Gly/Gly | Y/Y |
| GG79 | Estonia/Lõo | E11 | Arg/Gly | W/Y |
| GG80 | Estonia/mix of Vormsi and Keedika | E01 | Arg/Gly | Y/Y |
| GG81 | Estonia/mix of Vormsi and Keedika | E01 | Gly/Gly | Y/Y |
| GG82 | Estonia/mix of Vormsi and Keedika | E01 | Gly/Gly | Y/Y |
| GG83 | Estonia/Lõo | E11 | Arg/Gly | W/W |
| GG84 | Estonia/Lõo | E11 | Arg/Gly | W/W |
| GG85 | Estonia/Lõo | E11 | Arg/Gly | W/W |
| GG86 | Estonia/Lõo | E11 | Gly/Gly | W/Y |
| GG87 | Estonia/Lõo | E11 | Arg/Gly | W/W |
| GG88 | Estonia/Lõo | E11 | Arg/Gly | W/W |
| GG89 | Estonia/Lõo | E11 | Gly/Gly | Y/Y |
| GG90 | Estonia/Lõo | E11 | Arg/Gly | W/W |
| GG91 | Estonia/Lõo | E11 | Gly/Gly | W/Y |
| GG92 | Estonia/Lõo | E11 | Gly/Gly | W/W |
| GG93 | Estonia/Võru | E01 | Gly/Gly | W/Y |
| GG94 | Estonia/Võru | E01 | Gly/Gly | W/Y |
| GG95 | Estonia/Võru | E01 | Gly/Gly | W/Y |
| GG96 | Estonia/Võru | E01 | Gly/Gly | W/Y |
| GG97 | Estonia/Võru | E01 | Gly/Gly | W/W |
| GG98 | Estonia/mix of Vormsi and Keedika | E01 | Arg/Gly | Y/Y |

| | | | | |
|---|---|---|---|---|
| GG99 | Estonia/mix of Vormsi, Keedika and Liivi | E09 | Arg/Arg | Y/Y |
| GG100 | Estonia/mix of Vormsi and Keedika or mix of Vormsi, Keedika and Liivi | E09 | Gly/Gly | Y/Y |
| GG101 | Estonia/mix of Vormsi, Keedika and Liivi | E11 | Gly/Gly | Y/Y |
| GG102 | Estonia/mix of Vormsi and Keedika | E11 | Arg/Gly | Y/Y |
| GG103 | Estonia/Linnamäe | E11 | Gly/Gly | Y/Y |

**References**

Ehrlich, F., Rannamäe, E., Laneman, M., Tõrv, M., Lang, V., Oras, E., & Lõugas, L. (2021). In search of Estonia's earliest chicken. Estonian Journal of Archaeology, 25(2), 160. https://doi.org/10.3176/arch.2021.2.04
