# Supplementary material for: Ancient DNA unravels the history of chickens in the Baltic Sea region and the continuity of landrace lineages
Source: Heredity (Edinb). 2026 Apr 13;135(6):397–408. doi: 10.1038/s41437-026-00842-9 (PMC13354571; doi:10.1038/s41437-026-00842-9)
Supplement: Supplementary file 3 — Supplementary information [file 41437_2026_842_MOESM3_ESM.pdf]

Table S3. GenBank accession numbers and associated citations for sequences used as chicken (*Gallus gallus domesticus*) reference sequences for mitochondrial control region analysis.

GenBank accession number	Haplogroup	Haplotype	Reference
**Modern references**			
GU447341	A	A01	Miao, Y.-W., Peng, M.-S., Wu, G.-S., Ouyang, Y.-N., Yang, Z.-Y., Yu, N., Liang, J.-P., Pianchou, G., Beja-Pereira, A., Mitra, B., Palanichamy, M. G., Baig, M., Chaudhuri, T. K., Shen, Y.-Y., Kong, Q.-P., Murphy, R. W., Yao, Y.-G., & Zhang, Y.-P. (2013). Chicken domestication: an updated perspective based on mitochondrial genomes. Heredity, 110(3), 277–282. https://doi.org/10.1038/hdy.2012.83
GU447807	A	A02	Miao, Y.-W., Peng, M.-S., Wu, G.-S., Ouyang, Y.-N., Yang, Z.-Y., Yu, N., Liang, J.-P., Pianchou, G., Beja-Pereira, A., Mitra, B., Palanichamy, M. G., Baig, M., Chaudhuri, T. K., Shen, Y.-Y., Kong, Q.-P., Murphy, R. W., Yao, Y.-G., & Zhang, Y.-P. (2013). Chicken domestication: an updated perspective based on mitochondrial genomes. Heredity, 110(3), 277–282. https://doi.org/10.1038/hdy.2012.83
HQ022886	A	A03	Chang CS, Chen CF, Berthouly-Salazar C, Chazara O, Lee YP, Chang CM et al (2012). A global analysis of molecular markers and phenotypic traits in local chicken breeds in Taiwan. Anim Genet 43: 172–182. https://doi.org/10.1111/j.1365-2052.2011.02226.x
D82921	A	A04	Fumihito A, Miyake T, Takada M, Shingu R, Endo T, Gojobori T et al (1996). Monophyletic origin and unique dispersal patterns of domestic fowls. Proc Natl Acad Sci USA 93: 6792–6795. https://doi.org/10.1073/pnas.93.13.6792
GU448571	A	A05	Miao, Y.-W., Peng, M.-S., Wu, G.-S., Ouyang, Y.-N., Yang, Z.-Y., Yu, N., Liang, J.-P., Pianchou, G., Beja-Pereira, A., Mitra, B., Palanichamy, M. G., Baig, M., Chaudhuri, T. K., Shen, Y.-Y., Kong, Q.-P., Murphy, R. W., Yao, Y.-G., & Zhang, Y.-P. (2013). Chicken domestication: an updated perspective based on mitochondrial genomes. Heredity, 110(3), 277–282. https://doi.org/10.1038/hdy.2012.83
GU448536	A	A06	Miao, Y.-W., Peng, M.-S., Wu, G.-S., Ouyang, Y.-N., Yang, Z.-Y., Yu, N., Liang, J.-P., Pianchou, G., Beja-Pereira, A., Mitra, B., Palanichamy, M. G., Baig, M., Chaudhuri, T. K., Shen, Y.-Y., Kong, Q.-P., Murphy, R. W., Yao, Y.-G., & Zhang, Y.-P. (2013). Chicken domestication: an updated perspective based on mitochondrial genomes. Heredity, 110(3), 277–282. https://doi.org/10.1038/hdy.2012.83
GU448535	A	A07	Miao, Y.-W., Peng, M.-S., Wu, G.-S., Ouyang, Y.-N., Yang, Z.-Y., Yu, N., Liang, J.-P., Pianchou, G., Beja-Pereira, A., Mitra, B., Palanichamy, M. G., Baig, M., Chaudhuri, T. K., Shen, Y.-Y., Kong, Q.-P., Murphy, R. W., Yao, Y.-G., & Zhang, Y.-P. (2013). Chicken domestication: an updated perspective based on mitochondrial genomes. Heredity, 110(3), 277–282. https://doi.org/10.1038/hdy.2012.83
GU447548	A	A08	Miao, Y.-W., Peng, M.-S., Wu, G.-S., Ouyang, Y.-N., Yang, Z.-Y., Yu, N., Liang, J.-P., Pianchou, G., Beja-Pereira, A., Mitra, B., Palanichamy, M. G., Baig, M., Chaudhuri, T. K., Shen, Y.-Y., Kong, Q.-P., Murphy, R. W., Yao, Y.-G., & Zhang, Y.-P. (2013). Chicken domestication: an updated perspective based on mitochondrial genomes. Heredity, 110(3), 277–282. https://doi.org/10.1038/hdy.2012.83
GU447419	A	A09	Miao, Y.-W., Peng, M.-S., Wu, G.-S., Ouyang, Y.-N., Yang, Z.-Y., Yu, N., Liang, J.-P., Pianchou, G., Beja-Pereira, A., Mitra, B., Palanichamy, M. G., Baig, M., Chaudhuri, T. K., Shen, Y.-Y., Kong, Q.-P., Murphy, R. W., Yao, Y.-G., & Zhang, Y.-P. (2013). Chicken domestication: an updated perspective based on mitochondrial genomes. Heredity, 110(3), 277–282. https://doi.org/10.1038/hdy.2012.83
GU448549	A	A11	Miao, Y.-W., Peng, M.-S., Wu, G.-S., Ouyang, Y.-N., Yang, Z.-Y., Yu, N., Liang, J.-P., Pianchou, G., Beja-Pereira, A., Mitra, B., Palanichamy, M. G., Baig, M., Chaudhuri, T. K., Shen, Y.-Y., Kong, Q.-P., Murphy, R. W., Yao, Y.-G., & Zhang, Y.-P. (2013). Chicken domestication: an updated perspective based on mitochondrial genomes. Heredity, 110(3), 277–282. https://doi.org/10.1038/hdy.2012.83
AF512098	A	A12	Liu, Y. P., Wu, G. S., Yao, Y. G., Miao, Y. W., Luikart, G., Baig, M., Beja-Pereira, A., Ding, Z. L., Palanichamy, M. G., & Zhang, Y. P. (2006). Multiple maternal origins of chickens: out of the Asian jungles. *Molecular phylogenetics and evolution*, *38* (1), 12–19. https://doi.org/10.1016/j.ympev.2005.09.014
AF512096	A	A13	Liu, Y. P., Wu, G. S., Yao, Y. G., Miao, Y. W., Luikart, G., Baig, M., Beja-Pereira, A., Ding, Z. L., Palanichamy, M. G., & Zhang, Y. P. (2006). Multiple maternal origins of chickens: out of the Asian jungles. *Molecular phylogenetics and evolution*, *38* (1), 12–19. https://doi.org/10.1016/j.ympev.2005.09.014
AF512077	A	A14	Liu, Y. P., Wu, G. S., Yao, Y. G., Miao, Y. W., Luikart, G., Baig, M., Beja-Pereira, A., Ding, Z. L., Palanichamy, M. G., & Zhang, Y. P. (2006). Multiple maternal origins of chickens: out of the Asian jungles. *Molecular phylogenetics and evolution*, *38* (1), 12–19. https://doi.org/10.1016/j.ympev.2005.09.014
GU447344	A	A15	Miao, Y.-W., Peng, M.-S., Wu, G.-S., Ouyang, Y.-N., Yang, Z.-Y., Yu, N., Liang, J.-P., Pianchou, G., Beja-Pereira, A., Mitra, B., Palanichamy, M. G., Baig, M., Chaudhuri, T. K., Shen, Y.-Y., Kong, Q.-P., Murphy, R. W., Yao, Y.-G., & Zhang, Y.-P. (2013). Chicken domestication: an updated perspective based on mitochondrial genomes. Heredity, 110(3), 277–282. https://doi.org/10.1038/hdy.2012.83
AF512232	A	A16	Liu, Y. P., Wu, G. S., Yao, Y. G., Miao, Y. W., Luikart, G., Baig, M., Beja-Pereira, A., Ding, Z. L., Palanichamy, M. G., & Zhang, Y. P. (2006). Multiple maternal origins of chickens: out of the Asian jungles. *Molecular phylogenetics and evolution*, *38* (1), 12–19. https://doi.org/10.1016/j.ympev.2005.09.014
GU448030	A	A17	Miao, Y.-W., Peng, M.-S., Wu, G.-S., Ouyang, Y.-N., Yang, Z.-Y., Yu, N., Liang, J.-P., Pianchou, G., Beja-Pereira, A., Mitra, B., Palanichamy, M. G., Baig, M., Chaudhuri, T. K., Shen, Y.-Y., Kong, Q.-P., Murphy, R. W., Yao, Y.-G., & Zhang, Y.-P. (2013). Chicken domestication: an updated perspective based on mitochondrial genomes. Heredity, 110(3), 277–282. https://doi.org/10.1038/hdy.2012.83
GU448229	A	A18	Miao, Y.-W., Peng, M.-S., Wu, G.-S., Ouyang, Y.-N., Yang, Z.-Y., Yu, N., Liang, J.-P., Pianchou, G., Beja-Pereira, A., Mitra, B., Palanichamy, M. G., Baig, M., Chaudhuri, T. K., Shen, Y.-Y., Kong, Q.-P., Murphy, R. W., Yao, Y.-G., & Zhang, Y.-P. (2013). Chicken domestication: an updated perspective based on mitochondrial genomes. Heredity, 110(3), 277–282. https://doi.org/10.1038/hdy.2012.83
GU448900	A	A19	Miao, Y.-W., Peng, M.-S., Wu, G.-S., Ouyang, Y.-N., Yang, Z.-Y., Yu, N., Liang, J.-P., Pianchou, G., Beja-Pereira, A., Mitra, B., Palanichamy, M. G., Baig, M., Chaudhuri, T. K., Shen, Y.-Y., Kong, Q.-P., Murphy, R. W., Yao, Y.-G., & Zhang, Y.-P. (2013). Chicken domestication: an updated perspective based on mitochondrial genomes. Heredity, 110(3), 277–282. https://doi.org/10.1038/hdy.2012.83
GU448070	A	A20	Miao, Y.-W., Peng, M.-S., Wu, G.-S., Ouyang, Y.-N., Yang, Z.-Y., Yu, N., Liang, J.-P., Pianchou, G., Beja-Pereira, A., Mitra, B., Palanichamy, M. G., Baig, M., Chaudhuri, T. K., Shen, Y.-Y., Kong, Q.-P., Murphy, R. W., Yao, Y.-G., & Zhang, Y.-P. (2013). Chicken domestication: an updated perspective based on mitochondrial genomes. Heredity, 110(3), 277–282. https://doi.org/10.1038/hdy.2012.83
GU448902	A	A21	Miao, Y.-W., Peng, M.-S., Wu, G.-S., Ouyang, Y.-N., Yang, Z.-Y., Yu, N., Liang, J.-P., Pianchou, G., Beja-Pereira, A., Mitra, B., Palanichamy, M. G., Baig, M., Chaudhuri, T. K., Shen, Y.-Y., Kong, Q.-P., Murphy, R. W., Yao, Y.-G., & Zhang, Y.-P. (2013). Chicken domestication: an updated perspective based on mitochondrial genomes. Heredity, 110(3), 277–282. https://doi.org/10.1038/hdy.2012.83
AY392248	A	A22	Liu, Y. P., Wu, G. S., Yao, Y. G., Miao, Y. W., Luikart, G., Baig, M., Beja-Pereira, A., Ding, Z. L., Palanichamy, M. G., & Zhang, Y. P. (2006). Multiple maternal origins of chickens: out of the Asian jungles. *Molecular phylogenetics and evolution*, *38* (1), 12–19. https://doi.org/10.1016/j.ympev.2005.09.014
AY392301	A	A23	Liu, Y. P., Wu, G. S., Yao, Y. G., Miao, Y. W., Luikart, G., Baig, M., Beja-Pereira, A., Ding, Z. L., Palanichamy, M. G., & Zhang, Y. P. (2006). Multiple maternal origins of chickens: out of the Asian jungles. *Molecular phylogenetics and evolution*, *38* (1), 12–19. https://doi.org/10.1016/j.ympev.2005.09.014
AY465960	A	A24	Liu, Y. P., Wu, G. S., Yao, Y. G., Miao, Y. W., Luikart, G., Baig, M., Beja-Pereira, A., Ding, Z. L., Palanichamy, M. G., & Zhang, Y. P. (2006). Multiple maternal origins of chickens: out of the Asian jungles. *Molecular phylogenetics and evolution*, *38* (1), 12–19. https://doi.org/10.1016/j.ympev.2005.09.014
GU447347	A	A25	Miao, Y.-W., Peng, M.-S., Wu, G.-S., Ouyang, Y.-N., Yang, Z.-Y., Yu, N., Liang, J.-P., Pianchou, G., Beja-Pereira, A., Mitra, B., Palanichamy, M. G., Baig, M., Chaudhuri, T. K., Shen, Y.-Y., Kong, Q.-P., Murphy, R. W., Yao, Y.-G., & Zhang, Y.-P. (2013). Chicken domestication: an updated perspective based on mitochondrial genomes. Heredity, 110(3), 277–282. https://doi.org/10.1038/hdy.2012.83
AB098670	A	A26	Komiyama, T., Ikeo, K., & Gojobori, T. (2003). Where is the origin of the Japanese gamecocks?. *Gene*, *317* (1-2), 195–202. https://doi.org/10.1016/s0378-1119(03)00703-0
AY645000	A	A27	Liu, Y. P., Wu, G. S., Yao, Y. G., Miao, Y. W., Luikart, G., Baig, M., Beja-Pereira, A., Ding, Z. L., Palanichamy, M. G., & Zhang, Y. P. (2006). Multiple maternal origins of chickens: out of the Asian jungles. *Molecular phylogenetics and evolution*, *38* (1), 12–19. https://doi.org/10.1016/j.ympev.2005.09.014
AB114073	A	A28	Komiyama, T., Ikeo, K., & Gojobori, T. (2004). The evolutionary origin of long-crowing chicken: its evolutionary relationship with fighting cocks disclosed by the mtDNA sequence analysis. *Gene*, *333*, 91–99. https://doi.org/10.1016/j.gene.2004.02.035
AB114078	A	A29	Komiyama, T., Ikeo, K., & Gojobori, T. (2004). The evolutionary origin of long-crowing chicken: its evolutionary relationship with fighting cocks disclosed by the mtDNA sequence analysis. *Gene*, *333*, 91–99. https://doi.org/10.1016/j.gene.2004.02.035
AB114081	A	A30	Komiyama, T., Ikeo, K., & Gojobori, T. (2004). The evolutionary origin of long-crowing chicken: its evolutionary relationship with fighting cocks disclosed by the mtDNA sequence analysis. *Gene*, *333*, 91–99. https://doi.org/10.1016/j.gene.2004.02.035
AB114084	A	A31	Komiyama, T., Ikeo, K., & Gojobori, T. (2004). The evolutionary origin of long-crowing chicken: its evolutionary relationship with fighting cocks disclosed by the mtDNA sequence analysis. *Gene*, *333*, 91–99. https://doi.org/10.1016/j.gene.2004.02.035
AB114085	A	A32	Komiyama, T., Ikeo, K., & Gojobori, T. (2004). The evolutionary origin of long-crowing chicken: its evolutionary relationship with fighting cocks disclosed by the mtDNA sequence analysis. *Gene*, *333*, 91–99. https://doi.org/10.1016/j.gene.2004.02.035
AY465977	A	A33	Liu et al. 2003 Direct Submission
AY588608	A	A34	Qu et al. 2004 Direct Submission
AM746039	A	A35	Muchadeyi, F. C., Eding, H., Simianer, H., Wollny, C. B., Groeneveld, E., & Weigend, S. (2008). Mitochondrial DNA D-loop sequences suggest a Southeast Asian and Indian origin of Zimbabwean village chickens. *Animal genetics*, *39* (6), 615–622. https://doi.org/10.1111/j.1365-2052.2008.01785.x
GU448825	A	A36	Miao, Y.-W., Peng, M.-S., Wu, G.-S., Ouyang, Y.-N., Yang, Z.-Y., Yu, N., Liang, J.-P., Pianchou, G., Beja-Pereira, A., Mitra, B., Palanichamy, M. G., Baig, M., Chaudhuri, T. K., Shen, Y.-Y., Kong, Q.-P., Murphy, R. W., Yao, Y.-G., & Zhang, Y.-P. (2013). Chicken domestication: an updated perspective based on mitochondrial genomes. Heredity, 110(3), 277–282. https://doi.org/10.1038/hdy.2012.83
AY588624	A	A37	Qu et al. 2004 Direct Submission
GU448832	A	A38	Miao, Y.-W., Peng, M.-S., Wu, G.-S., Ouyang, Y.-N., Yang, Z.-Y., Yu, N., Liang, J.-P., Pianchou, G., Beja-Pereira, A., Mitra, B., Palanichamy, M. G., Baig, M., Chaudhuri, T. K., Shen, Y.-Y., Kong, Q.-P., Murphy, R. W., Yao, Y.-G., & Zhang, Y.-P. (2013). Chicken domestication: an updated perspective based on mitochondrial genomes. Heredity, 110(3), 277–282. https://doi.org/10.1038/hdy.2012.83

GU447399	A	A39	Miao, Y.-W., Peng, M.-S., Wu, G.-S., Ouyang, Y.-N., Yang, Z.-Y., Yu, N., Liang, J.-P., Pianchou, G., Beja-Pereira, A., Mitra, B., Palanichamy, M. G., Baig, M., Chaudhuri, T. K., Shen, Y.-Y., Kong, Q.-P., Murphy, R. W., Yao, Y.-G., & Zhang, Y.-P. (2013). Chicken domestication: an updated perspective based on mitochondrial genomes. Heredity, 110(3), 277–282. https://doi.org/10.1038/hdy.2012.83
GU447951	A	A40	Miao, Y.-W., Peng, M.-S., Wu, G.-S., Ouyang, Y.-N., Yang, Z.-Y., Yu, N., Liang, J.-P., Pianchou, G., Beja-Pereira, A., Mitra, B., Palanichamy, M. G., Baig, M., Chaudhuri, T. K., Shen, Y.-Y., Kong, Q.-P., Murphy, R. W., Yao, Y.-G., & Zhang, Y.-P. (2013). Chicken domestication: an updated perspective based on mitochondrial genomes. Heredity, 110(3), 277–282. https://doi.org/10.1038/hdy.2012.83
GU448800	A	A41	Miao, Y.-W., Peng, M.-S., Wu, G.-S., Ouyang, Y.-N., Yang, Z.-Y., Yu, N., Liang, J.-P., Pianchou, G., Beja-Pereira, A., Mitra, B., Palanichamy, M. G., Baig, M., Chaudhuri, T. K., Shen, Y.-Y., Kong, Q.-P., Murphy, R. W., Yao, Y.-G., & Zhang, Y.-P. (2013). Chicken domestication: an updated perspective based on mitochondrial genomes. Heredity, 110(3), 277–282. https://doi.org/10.1038/hdy.2012.83
GU448664	A	A42	Miao, Y.-W., Peng, M.-S., Wu, G.-S., Ouyang, Y.-N., Yang, Z.-Y., Yu, N., Liang, J.-P., Pianchou, G., Beja-Pereira, A., Mitra, B., Palanichamy, M. G., Baig, M., Chaudhuri, T. K., Shen, Y.-Y., Kong, Q.-P., Murphy, R. W., Yao, Y.-G., & Zhang, Y.-P. (2013). Chicken domestication: an updated perspective based on mitochondrial genomes. Heredity, 110(3), 277–282. https://doi.org/10.1038/hdy.2012.83
GU447395	A	A43	Miao, Y.-W., Peng, M.-S., Wu, G.-S., Ouyang, Y.-N., Yang, Z.-Y., Yu, N., Liang, J.-P., Pianchou, G., Beja-Pereira, A., Mitra, B., Palanichamy, M. G., Baig, M., Chaudhuri, T. K., Shen, Y.-Y., Kong, Q.-P., Murphy, R. W., Yao, Y.-G., & Zhang, Y.-P. (2013). Chicken domestication: an updated perspective based on mitochondrial genomes. Heredity, 110(3), 277–282. https://doi.org/10.1038/hdy.2012.83
GU448605	A	A44	Miao, Y.-W., Peng, M.-S., Wu, G.-S., Ouyang, Y.-N., Yang, Z.-Y., Yu, N., Liang, J.-P., Pianchou, G., Beja-Pereira, A., Mitra, B., Palanichamy, M. G., Baig, M., Chaudhuri, T. K., Shen, Y.-Y., Kong, Q.-P., Murphy, R. W., Yao, Y.-G., & Zhang, Y.-P. (2013). Chicken domestication: an updated perspective based on mitochondrial genomes. Heredity, 110(3), 277–282. https://doi.org/10.1038/hdy.2012.83
GU447892	A	A45	Miao, Y.-W., Peng, M.-S., Wu, G.-S., Ouyang, Y.-N., Yang, Z.-Y., Yu, N., Liang, J.-P., Pianchou, G., Beja-Pereira, A., Mitra, B., Palanichamy, M. G., Baig, M., Chaudhuri, T. K., Shen, Y.-Y., Kong, Q.-P., Murphy, R. W., Yao, Y.-G., & Zhang, Y.-P. (2013). Chicken domestication: an updated perspective based on mitochondrial genomes. Heredity, 110(3), 277–282. https://doi.org/10.1038/hdy.2012.83
GU447339	A	A46	Miao, Y.-W., Peng, M.-S., Wu, G.-S., Ouyang, Y.-N., Yang, Z.-Y., Yu, N., Liang, J.-P., Pianchou, G., Beja-Pereira, A., Mitra, B., Palanichamy, M. G., Baig, M., Chaudhuri, T. K., Shen, Y.-Y., Kong, Q.-P., Murphy, R. W., Yao, Y.-G., & Zhang, Y.-P. (2013). Chicken domestication: an updated perspective based on mitochondrial genomes. Heredity, 110(3), 277–282. https://doi.org/10.1038/hdy.2012.83
GU448833	A	A47	Miao, Y.-W., Peng, M.-S., Wu, G.-S., Ouyang, Y.-N., Yang, Z.-Y., Yu, N., Liang, J.-P., Pianchou, G., Beja-Pereira, A., Mitra, B., Palanichamy, M. G., Baig, M., Chaudhuri, T. K., Shen, Y.-Y., Kong, Q.-P., Murphy, R. W., Yao, Y.-G., & Zhang, Y.-P. (2013). Chicken domestication: an updated perspective based on mitochondrial genomes. Heredity, 110(3), 277–282. https://doi.org/10.1038/hdy.2012.83
GU447954	A	A48	Miao, Y.-W., Peng, M.-S., Wu, G.-S., Ouyang, Y.-N., Yang, Z.-Y., Yu, N., Liang, J.-P., Pianchou, G., Beja-Pereira, A., Mitra, B., Palanichamy, M. G., Baig, M., Chaudhuri, T. K., Shen, Y.-Y., Kong, Q.-P., Murphy, R. W., Yao, Y.-G., & Zhang, Y.-P. (2013). Chicken domestication: an updated perspective based on mitochondrial genomes. Heredity, 110(3), 277–282. https://doi.org/10.1038/hdy.2012.83
GU447703	A	A49	Miao, Y.-W., Peng, M.-S., Wu, G.-S., Ouyang, Y.-N., Yang, Z.-Y., Yu, N., Liang, J.-P., Pianchou, G., Beja-Pereira, A., Mitra, B., Palanichamy, M. G., Baig, M., Chaudhuri, T. K., Shen, Y.-Y., Kong, Q.-P., Murphy, R. W., Yao, Y.-G., & Zhang, Y.-P. (2013). Chicken domestication: an updated perspective based on mitochondrial genomes. Heredity, 110(3), 277–282. https://doi.org/10.1038/hdy.2012.83
GU448228	A	A50	Miao, Y.-W., Peng, M.-S., Wu, G.-S., Ouyang, Y.-N., Yang, Z.-Y., Yu, N., Liang, J.-P., Pianchou, G., Beja-Pereira, A., Mitra, B., Palanichamy, M. G., Baig, M., Chaudhuri, T. K., Shen, Y.-Y., Kong, Q.-P., Murphy, R. W., Yao, Y.-G., & Zhang, Y.-P. (2013). Chicken domestication: an updated perspective based on mitochondrial genomes. Heredity, 110(3), 277–282. https://doi.org/10.1038/hdy.2012.83
DQ462562	A	A51	Song and Jiang. 2006 Direct Submission
GU448992	A	A52	Miao, Y.-W., Peng, M.-S., Wu, G.-S., Ouyang, Y.-N., Yang, Z.-Y., Yu, N., Liang, J.-P., Pianchou, G., Beja-Pereira, A., Mitra, B., Palanichamy, M. G., Baig, M., Chaudhuri, T. K., Shen, Y.-Y., Kong, Q.-P., Murphy, R. W., Yao, Y.-G., & Zhang, Y.-P. (2013). Chicken domestication: an updated perspective based on mitochondrial genomes. Heredity, 110(3), 277–282. https://doi.org/10.1038/hdy.2012.83
GU448334	A	A53	Miao, Y.-W., Peng, M.-S., Wu, G.-S., Ouyang, Y.-N., Yang, Z.-Y., Yu, N., Liang, J.-P., Pianchou, G., Beja-Pereira, A., Mitra, B., Palanichamy, M. G., Baig, M., Chaudhuri, T. K., Shen, Y.-Y., Kong, Q.-P., Murphy, R. W., Yao, Y.-G., & Zhang, Y.-P. (2013). Chicken domestication: an updated perspective based on mitochondrial genomes. Heredity, 110(3), 277–282. https://doi.org/10.1038/hdy.2012.83
GU447829	A	A55	Miao, Y.-W., Peng, M.-S., Wu, G.-S., Ouyang, Y.-N., Yang, Z.-Y., Yu, N., Liang, J.-P., Pianchou, G., Beja-Pereira, A., Mitra, B., Palanichamy, M. G., Baig, M., Chaudhuri, T. K., Shen, Y.-Y., Kong, Q.-P., Murphy, R. W., Yao, Y.-G., & Zhang, Y.-P. (2013). Chicken domestication: an updated perspective based on mitochondrial genomes. Heredity, 110(3), 277–282. https://doi.org/10.1038/hdy.2012.83
GU448307	A	A56	Miao, Y.-W., Peng, M.-S., Wu, G.-S., Ouyang, Y.-N., Yang, Z.-Y., Yu, N., Liang, J.-P., Pianchou, G., Beja-Pereira, A., Mitra, B., Palanichamy, M. G., Baig, M., Chaudhuri, T. K., Shen, Y.-Y., Kong, Q.-P., Murphy, R. W., Yao, Y.-G., & Zhang, Y.-P. (2013). Chicken domestication: an updated perspective based on mitochondrial genomes. Heredity, 110(3), 277–282. https://doi.org/10.1038/hdy.2012.83
DQ462521	A	A57	Song and Jiang. 2006 Direct Submission
DQ462548	A	A58	Song and Jiang. 2006 Direct Submission
GU448621	A	A59	Miao, Y.-W., Peng, M.-S., Wu, G.-S., Ouyang, Y.-N., Yang, Z.-Y., Yu, N., Liang, J.-P., Pianchou, G., Beja-Pereira, A., Mitra, B., Palanichamy, M. G., Baig, M., Chaudhuri, T. K., Shen, Y.-Y., Kong, Q.-P., Murphy, R. W., Yao, Y.-G., & Zhang, Y.-P. (2013). Chicken domestication: an updated perspective based on mitochondrial genomes. Heredity, 110(3), 277–282. https://doi.org/10.1038/hdy.2012.83
GU447332	A	A60	Miao, Y.-W., Peng, M.-S., Wu, G.-S., Ouyang, Y.-N., Yang, Z.-Y., Yu, N., Liang, J.-P., Pianchou, G., Beja-Pereira, A., Mitra, B., Palanichamy, M. G., Baig, M., Chaudhuri, T. K., Shen, Y.-Y., Kong, Q.-P., Murphy, R. W., Yao, Y.-G., & Zhang, Y.-P. (2013). Chicken domestication: an updated perspective based on mitochondrial genomes. Heredity, 110(3), 277–282. https://doi.org/10.1038/hdy.2012.83
DQ462563	A	A61	Song and Jiang. 2006 Direct Submission
DQ462553	A	A62	Song and Jiang. 2006 Direct Submission
AF512061	A	A63	Liu, Y. P., Wu, G. S., Yao, Y. G., Miao, Y. W., Luikart, G., Baig, M., Beja-Pereira, A., Ding, Z. L., Palanichamy, M. G., & Zhang, Y. P. (2006). Multiple maternal origins of chickens: out of the Asian jungles. *Molecular phylogenetics and evolution* , *38* (1), 12–19. https://doi.org/10.1016/j.ympev.2005.09.014
DQ462561	A	A64	Song and Jiang. 2006 Direct Submission
EU847752	A	A65	Kanginakudru, S., Metta, M., Jakati, R. D., & Nagaraju, J. (2008). Genetic evidence from Indian red jungle fowl corroborates multiple domestication of modern day chicken. *BMC evolutionary biology* , *8* , 174. https://doi.org/10.1186/1471-2148-8-174
GU448156	A	A66	Miao, Y.-W., Peng, M.-S., Wu, G.-S., Ouyang, Y.-N., Yang, Z.-Y., Yu, N., Liang, J.-P., Pianchou, G., Beja-Pereira, A., Mitra, B., Palanichamy, M. G., Baig, M., Chaudhuri, T. K., Shen, Y.-Y., Kong, Q.-P., Murphy, R. W., Yao, Y.-G., & Zhang, Y.-P. (2013). Chicken domestication: an updated perspective based on mitochondrial genomes. Heredity, 110(3), 277–282. https://doi.org/10.1038/hdy.2012.83
GU448684	A	A67	Miao, Y.-W., Peng, M.-S., Wu, G.-S., Ouyang, Y.-N., Yang, Z.-Y., Yu, N., Liang, J.-P., Pianchou, G., Beja-Pereira, A., Mitra, B., Palanichamy, M. G., Baig, M., Chaudhuri, T. K., Shen, Y.-Y., Kong, Q.-P., Murphy, R. W., Yao, Y.-G., & Zhang, Y.-P. (2013). Chicken domestication: an updated perspective based on mitochondrial genomes. Heredity, 110(3), 277–282. https://doi.org/10.1038/hdy.2012.83
GU447957	A	A68	Miao, Y.-W., Peng, M.-S., Wu, G.-S., Ouyang, Y.-N., Yang, Z.-Y., Yu, N., Liang, J.-P., Pianchou, G., Beja-Pereira, A., Mitra, B., Palanichamy, M. G., Baig, M., Chaudhuri, T. K., Shen, Y.-Y., Kong, Q.-P., Murphy, R. W., Yao, Y.-G., & Zhang, Y.-P. (2013). Chicken domestication: an updated perspective based on mitochondrial genomes. Heredity, 110(3), 277–282. https://doi.org/10.1038/hdy.2012.83
AB368436	A	A69	Sakahira 2007 Direct Submission
AB368430	A	A70	Sakahira 2007 Direct Submission
HM462123	A	A71	Berthouly-Salazar, C., Rognon, X., Van, T., Gély, M., Chi, C. V., Tixier-Boichard, M., Bed'Hom, B., Bruneau, N., Verrier, E., Maillard, J. C., & Michaux, J. R. (2010). Vietnamese chickens: a gate towards Asian genetic diversity. *BMC genetics* , *11* , 53. https://doi.org/10.1186/1471-2156-11-53
HM462124	A	A72	Berthouly-Salazar, C., Rognon, X., Van, T., Gély, M., Chi, C. V., Tixier-Boichard, M., Bed'Hom, B., Bruneau, N., Verrier, E., Maillard, J. C., & Michaux, J. R. (2010). Vietnamese chickens: a gate towards Asian genetic diversity. *BMC genetics* , *11* , 53. https://doi.org/10.1186/1471-2156-11-53
HQ189525	A	A73	Arora et al. 2010 Direct Submission
AM746034	A	A74	Muchadeyi, F. C., Eding, H., Simianer, H., Wollny, C. B., Groeneveld, E., & Weigend, S. (2008). Mitochondrial DNA D-loop sequences suggest a Southeast Asian and Indian origin of Zimbabwean village chickens. *Animal genetics* , *39* (6), 615–622. https://doi.org/10.1111/j.1365-2052.2008.01785.x
AM746038	A	A75	Muchadeyi, F. C., Eding, H., Simianer, H., Wollny, C. B., Groeneveld, E., & Weigend, S. (2008). Mitochondrial DNA D-loop sequences suggest a Southeast Asian and Indian origin of Zimbabwean village chickens. *Animal genetics* , *39* (6), 615–622. https://doi.org/10.1111/j.1365-2052.2008.01785.x
HM015606	A	A76	Dana, N., Megens, H. J., Crooijmans, R. P., Hanotte, O., Mwacharo, J., Groenen, M. A., & van Arendonk, J. A. (2011). East Asian contributions to Dutch traditional and western commercial chickens inferred from mtDNA analysis. *Animal genetics* , *42* (2), 125–133. https://doi.org/10.1111/j.1365-2052.2010.02134.x
GU448539	A	A77	Miao, Y.-W., Peng, M.-S., Wu, G.-S., Ouyang, Y.-N., Yang, Z.-Y., Yu, N., Liang, J.-P., Pianchou, G., Beja-Pereira, A., Mitra, B., Palanichamy, M. G., Baig, M., Chaudhuri, T. K., Shen, Y.-Y., Kong, Q.-P., Murphy, R. W., Yao, Y.-G., & Zhang, Y.-P. (2013). Chicken domestication: an updated perspective based on mitochondrial genomes. Heredity, 110(3), 277–282. https://doi.org/10.1038/hdy.2012.83
GU448678	A	A78	Miao, Y.-W., Peng, M.-S., Wu, G.-S., Ouyang, Y.-N., Yang, Z.-Y., Yu, N., Liang, J.-P., Pianchou, G., Beja-Pereira, A., Mitra, B., Palanichamy, M. G., Baig, M., Chaudhuri, T. K., Shen, Y.-Y., Kong, Q.-P., Murphy, R. W., Yao, Y.-G., & Zhang, Y.-P. (2013). Chicken domestication: an updated perspective based on mitochondrial genomes. Heredity, 110(3), 277–282. https://doi.org/10.1038/hdy.2012.83
GU447603	A	A79	Miao, Y.-W., Peng, M.-S., Wu, G.-S., Ouyang, Y.-N., Yang, Z.-Y., Yu, N., Liang, J.-P., Pianchou, G., Beja-Pereira, A., Mitra, B., Palanichamy, M. G., Baig, M., Chaudhuri, T. K., Shen, Y.-Y., Kong, Q.-P., Murphy, R. W., Yao, Y.-G., & Zhang, Y.-P. (2013). Chicken domestication: an updated perspective based on mitochondrial genomes. Heredity, 110(3), 277–282. https://doi.org/10.1038/hdy.2012.83

GU447342	B	B01	Miao, Y.-W., Peng, M.-S., Wu, G.-S., Ouyang, Y.-N., Yang, Z.-Y., Yu, N., Liang, J.-P., Pianchou, G., Beja-Pereira, A., Mitra, B., Palanichamy, M. G., Baig, M., Chaudhuri, T. K., Shen, Y.-Y., Kong, Q.-P., Murphy, R. W., Yao, Y.-G., & Zhang, Y.-P. (2013). Chicken domestication: an updated perspective based on mitochondrial genomes. Heredity, 110(3), 277–282. https://doi.org/10.1038/hdy.2012.83
AF512326	B	B02	Liu, Y. P., Wu, G. S., Yao, Y. G., Miao, Y. W., Luikart, G., Baig, M., Beja-Pereira, A., Ding, Z. L., Palanichamy, M. G., & Zhang, Y. P. (2006). Multiple maternal origins of chickens: out of the Asian jungles. *Molecular phylogenetics and evolution* , *38* (1), 12–19. https://doi.org/10.1016/j.ympev.2005.09.014
AF512309	B	B03	Liu, Y. P., Wu, G. S., Yao, Y. G., Miao, Y. W., Luikart, G., Baig, M., Beja-Pereira, A., Ding, Z. L., Palanichamy, M. G., & Zhang, Y. P. (2006). Multiple maternal origins of chickens: out of the Asian jungles. *Molecular phylogenetics and evolution* , *38* (1), 12–19. https://doi.org/10.1016/j.ympev.2005.09.014
AF512245	B	B04	Liu, Y. P., Wu, G. S., Yao, Y. G., Miao, Y. W., Luikart, G., Baig, M., Beja-Pereira, A., Ding, Z. L., Palanichamy, M. G., & Zhang, Y. P. (2006). Multiple maternal origins of chickens: out of the Asian jungles. *Molecular phylogenetics and evolution* , *38* (1), 12–19. https://doi.org/10.1016/j.ympev.2005.09.014
AF512089	B	B05	Liu, Y. P., Wu, G. S., Yao, Y. G., Miao, Y. W., Luikart, G., Baig, M., Beja-Pereira, A., Ding, Z. L., Palanichamy, M. G., & Zhang, Y. P. (2006). Multiple maternal origins of chickens: out of the Asian jungles. *Molecular phylogenetics and evolution* , *38* (1), 12–19. https://doi.org/10.1016/j.ympev.2005.09.014
AF512085	B	B06	Liu, Y. P., Wu, G. S., Yao, Y. G., Miao, Y. W., Luikart, G., Baig, M., Beja-Pereira, A., Ding, Z. L., Palanichamy, M. G., & Zhang, Y. P. (2006). Multiple maternal origins of chickens: out of the Asian jungles. *Molecular phylogenetics and evolution* , *38* (1), 12–19. https://doi.org/10.1016/j.ympev.2005.09.014
AF512299	B	B07	Liu, Y. P., Wu, G. S., Yao, Y. G., Miao, Y. W., Luikart, G., Baig, M., Beja-Pereira, A., Ding, Z. L., Palanichamy, M. G., & Zhang, Y. P. (2006). Multiple maternal origins of chickens: out of the Asian jungles. *Molecular phylogenetics and evolution* , *38* (1), 12–19. https://doi.org/10.1016/j.ympev.2005.09.014
GU447890	B	B08	Miao, Y.-W., Peng, M.-S., Wu, G.-S., Ouyang, Y.-N., Yang, Z.-Y., Yu, N., Liang, J.-P., Pianchou, G., Beja-Pereira, A., Mitra, B., Palanichamy, M. G., Baig, M., Chaudhuri, T. K., Shen, Y.-Y., Kong, Q.-P., Murphy, R. W., Yao, Y.-G., & Zhang, Y.-P. (2013). Chicken domestication: an updated perspective based on mitochondrial genomes. Heredity, 110(3), 277–282. https://doi.org/10.1038/hdy.2012.83
AF128335	B	B09	Fu et al. 1999 Direct Submission
GU447627	B	B10	Miao, Y.-W., Peng, M.-S., Wu, G.-S., Ouyang, Y.-N., Yang, Z.-Y., Yu, N., Liang, J.-P., Pianchou, G., Beja-Pereira, A., Mitra, B., Palanichamy, M. G., Baig, M., Chaudhuri, T. K., Shen, Y.-Y., Kong, Q.-P., Murphy, R. W., Yao, Y.-G., & Zhang, Y.-P. (2013). Chicken domestication: an updated perspective based on mitochondrial genomes. Heredity, 110(3), 277–282. https://doi.org/10.1038/hdy.2012.83
GU448010	B	B11	Miao, Y.-W., Peng, M.-S., Wu, G.-S., Ouyang, Y.-N., Yang, Z.-Y., Yu, N., Liang, J.-P., Pianchou, G., Beja-Pereira, A., Mitra, B., Palanichamy, M. G., Baig, M., Chaudhuri, T. K., Shen, Y.-Y., Kong, Q.-P., Murphy, R. W., Yao, Y.-G., & Zhang, Y.-P. (2013). Chicken domestication: an updated perspective based on mitochondrial genomes. Heredity, 110(3), 277–282. https://doi.org/10.1038/hdy.2012.83
AF128342	B	B12	Fu et al. 1999 Direct Submission
GU447558	B	B13	Miao, Y.-W., Peng, M.-S., Wu, G.-S., Ouyang, Y.-N., Yang, Z.-Y., Yu, N., Liang, J.-P., Pianchou, G., Beja-Pereira, A., Mitra, B., Palanichamy, M. G., Baig, M., Chaudhuri, T. K., Shen, Y.-Y., Kong, Q.-P., Murphy, R. W., Yao, Y.-G., & Zhang, Y.-P. (2013). Chicken domestication: an updated perspective based on mitochondrial genomes. Heredity, 110(3), 277–282. https://doi.org/10.1038/hdy.2012.83
GU447912	B	B14	Miao, Y.-W., Peng, M.-S., Wu, G.-S., Ouyang, Y.-N., Yang, Z.-Y., Yu, N., Liang, J.-P., Pianchou, G., Beja-Pereira, A., Mitra, B., Palanichamy, M. G., Baig, M., Chaudhuri, T. K., Shen, Y.-Y., Kong, Q.-P., Murphy, R. W., Yao, Y.-G., & Zhang, Y.-P. (2013). Chicken domestication: an updated perspective based on mitochondrial genomes. Heredity, 110(3), 277–282. https://doi.org/10.1038/hdy.2012.83
GU448051	B	B15	Miao, Y.-W., Peng, M.-S., Wu, G.-S., Ouyang, Y.-N., Yang, Z.-Y., Yu, N., Liang, J.-P., Pianchou, G., Beja-Pereira, A., Mitra, B., Palanichamy, M. G., Baig, M., Chaudhuri, T. K., Shen, Y.-Y., Kong, Q.-P., Murphy, R. W., Yao, Y.-G., & Zhang, Y.-P. (2013). Chicken domestication: an updated perspective based on mitochondrial genomes. Heredity, 110(3), 277–282. https://doi.org/10.1038/hdy.2012.83
AB098665	B	B16	Komiyama, T., Ikeo, K., & Gojobori, T. (2003). Where is the origin of the Japanese gamecocks?. *Gene* , *317* (1-2), 195–202. https://doi.org/10.1016/s0378-1119(03)00703-0
AB098667	B	B17	Komiyama, T., Ikeo, K., & Gojobori, T. (2003). Where is the origin of the Japanese gamecocks?. *Gene* , *317* (1-2), 195–202. https://doi.org/10.1016/s0378-1119(03)00703-0
AY465985	B	B18	Liu et al. 2003 Direct Submission
GU447956	B	B19	Miao, Y.-W., Peng, M.-S., Wu, G.-S., Ouyang, Y.-N., Yang, Z.-Y., Yu, N., Liang, J.-P., Pianchou, G., Beja-Pereira, A., Mitra, B., Palanichamy, M. G., Baig, M., Chaudhuri, T. K., Shen, Y.-Y., Kong, Q.-P., Murphy, R. W., Yao, Y.-G., & Zhang, Y.-P. (2013). Chicken domestication: an updated perspective based on mitochondrial genomes. Heredity, 110(3), 277–282. https://doi.org/10.1038/hdy.2012.83
AY588616	B	B20	Qu et al. 2004 Direct Submission
AY588629	B	B21	Qu et al. 2004 Direct Submission
AY588633	B	B22	Qu et al. 2004 Direct Submission
GU448064	B	B23	Miao, Y.-W., Peng, M.-S., Wu, G.-S., Ouyang, Y.-N., Yang, Z.-Y., Yu, N., Liang, J.-P., Pianchou, G., Beja-Pereira, A., Mitra, B., Palanichamy, M. G., Baig, M., Chaudhuri, T. K., Shen, Y.-Y., Kong, Q.-P., Murphy, R. W., Yao, Y.-G., & Zhang, Y.-P. (2013). Chicken domestication: an updated perspective based on mitochondrial genomes. Heredity, 110(3), 277–282. https://doi.org/10.1038/hdy.2012.83
GU448009	B	B24	Miao, Y.-W., Peng, M.-S., Wu, G.-S., Ouyang, Y.-N., Yang, Z.-Y., Yu, N., Liang, J.-P., Pianchou, G., Beja-Pereira, A., Mitra, B., Palanichamy, M. G., Baig, M., Chaudhuri, T. K., Shen, Y.-Y., Kong, Q.-P., Murphy, R. W., Yao, Y.-G., & Zhang, Y.-P. (2013). Chicken domestication: an updated perspective based on mitochondrial genomes. Heredity, 110(3), 277–282. https://doi.org/10.1038/hdy.2012.83
GU448567	B	B25	Miao, Y.-W., Peng, M.-S., Wu, G.-S., Ouyang, Y.-N., Yang, Z.-Y., Yu, N., Liang, J.-P., Pianchou, G., Beja-Pereira, A., Mitra, B., Palanichamy, M. G., Baig, M., Chaudhuri, T. K., Shen, Y.-Y., Kong, Q.-P., Murphy, R. W., Yao, Y.-G., & Zhang, Y.-P. (2013). Chicken domestication: an updated perspective based on mitochondrial genomes. Heredity, 110(3), 277–282. https://doi.org/10.1038/hdy.2012.83
GU448782	B	B26	Miao, Y.-W., Peng, M.-S., Wu, G.-S., Ouyang, Y.-N., Yang, Z.-Y., Yu, N., Liang, J.-P., Pianchou, G., Beja-Pereira, A., Mitra, B., Palanichamy, M. G., Baig, M., Chaudhuri, T. K., Shen, Y.-Y., Kong, Q.-P., Murphy, R. W., Yao, Y.-G., & Zhang, Y.-P. (2013). Chicken domestication: an updated perspective based on mitochondrial genomes. Heredity, 110(3), 277–282. https://doi.org/10.1038/hdy.2012.83
GU448502	B	B27	Miao, Y.-W., Peng, M.-S., Wu, G.-S., Ouyang, Y.-N., Yang, Z.-Y., Yu, N., Liang, J.-P., Pianchou, G., Beja-Pereira, A., Mitra, B., Palanichamy, M. G., Baig, M., Chaudhuri, T. K., Shen, Y.-Y., Kong, Q.-P., Murphy, R. W., Yao, Y.-G., & Zhang, Y.-P. (2013). Chicken domestication: an updated perspective based on mitochondrial genomes. Heredity, 110(3), 277–282. https://doi.org/10.1038/hdy.2012.83
GU448261	B	B28	Miao, Y.-W., Peng, M.-S., Wu, G.-S., Ouyang, Y.-N., Yang, Z.-Y., Yu, N., Liang, J.-P., Pianchou, G., Beja-Pereira, A., Mitra, B., Palanichamy, M. G., Baig, M., Chaudhuri, T. K., Shen, Y.-Y., Kong, Q.-P., Murphy, R. W., Yao, Y.-G., & Zhang, Y.-P. (2013). Chicken domestication: an updated perspective based on mitochondrial genomes. Heredity, 110(3), 277–282. https://doi.org/10.1038/hdy.2012.83
GU447414	B	B29	Miao, Y.-W., Peng, M.-S., Wu, G.-S., Ouyang, Y.-N., Yang, Z.-Y., Yu, N., Liang, J.-P., Pianchou, G., Beja-Pereira, A., Mitra, B., Palanichamy, M. G., Baig, M., Chaudhuri, T. K., Shen, Y.-Y., Kong, Q.-P., Murphy, R. W., Yao, Y.-G., & Zhang, Y.-P. (2013). Chicken domestication: an updated perspective based on mitochondrial genomes. Heredity, 110(3), 277–282. https://doi.org/10.1038/hdy.2012.83
GU447564	B	B30	Miao, Y.-W., Peng, M.-S., Wu, G.-S., Ouyang, Y.-N., Yang, Z.-Y., Yu, N., Liang, J.-P., Pianchou, G., Beja-Pereira, A., Mitra, B., Palanichamy, M. G., Baig, M., Chaudhuri, T. K., Shen, Y.-Y., Kong, Q.-P., Murphy, R. W., Yao, Y.-G., & Zhang, Y.-P. (2013). Chicken domestication: an updated perspective based on mitochondrial genomes. Heredity, 110(3), 277–282. https://doi.org/10.1038/hdy.2012.83
GU447475	B	B31	Miao, Y.-W., Peng, M.-S., Wu, G.-S., Ouyang, Y.-N., Yang, Z.-Y., Yu, N., Liang, J.-P., Pianchou, G., Beja-Pereira, A., Mitra, B., Palanichamy, M. G., Baig, M., Chaudhuri, T. K., Shen, Y.-Y., Kong, Q.-P., Murphy, R. W., Yao, Y.-G., & Zhang, Y.-P. (2013). Chicken domestication: an updated perspective based on mitochondrial genomes. Heredity, 110(3), 277–282. https://doi.org/10.1038/hdy.2012.83
GU447907	B	B32	Miao, Y.-W., Peng, M.-S., Wu, G.-S., Ouyang, Y.-N., Yang, Z.-Y., Yu, N., Liang, J.-P., Pianchou, G., Beja-Pereira, A., Mitra, B., Palanichamy, M. G., Baig, M., Chaudhuri, T. K., Shen, Y.-Y., Kong, Q.-P., Murphy, R. W., Yao, Y.-G., & Zhang, Y.-P. (2013). Chicken domestication: an updated perspective based on mitochondrial genomes. Heredity, 110(3), 277–282. https://doi.org/10.1038/hdy.2012.83
GU447660	B	B33	Miao, Y.-W., Peng, M.-S., Wu, G.-S., Ouyang, Y.-N., Yang, Z.-Y., Yu, N., Liang, J.-P., Pianchou, G., Beja-Pereira, A., Mitra, B., Palanichamy, M. G., Baig, M., Chaudhuri, T. K., Shen, Y.-Y., Kong, Q.-P., Murphy, R. W., Yao, Y.-G., & Zhang, Y.-P. (2013). Chicken domestication: an updated perspective based on mitochondrial genomes. Heredity, 110(3), 277–282. https://doi.org/10.1038/hdy.2012.83
GU448192	B	B34	Miao, Y.-W., Peng, M.-S., Wu, G.-S., Ouyang, Y.-N., Yang, Z.-Y., Yu, N., Liang, J.-P., Pianchou, G., Beja-Pereira, A., Mitra, B., Palanichamy, M. G., Baig, M., Chaudhuri, T. K., Shen, Y.-Y., Kong, Q.-P., Murphy, R. W., Yao, Y.-G., & Zhang, Y.-P. (2013). Chicken domestication: an updated perspective based on mitochondrial genomes. Heredity, 110(3), 277–282. https://doi.org/10.1038/hdy.2012.83
GU447429	B	B35	Miao, Y.-W., Peng, M.-S., Wu, G.-S., Ouyang, Y.-N., Yang, Z.-Y., Yu, N., Liang, J.-P., Pianchou, G., Beja-Pereira, A., Mitra, B., Palanichamy, M. G., Baig, M., Chaudhuri, T. K., Shen, Y.-Y., Kong, Q.-P., Murphy, R. W., Yao, Y.-G., & Zhang, Y.-P. (2013). Chicken domestication: an updated perspective based on mitochondrial genomes. Heredity, 110(3), 277–282. https://doi.org/10.1038/hdy.2012.83
GU447443	B	B36	Miao, Y.-W., Peng, M.-S., Wu, G.-S., Ouyang, Y.-N., Yang, Z.-Y., Yu, N., Liang, J.-P., Pianchou, G., Beja-Pereira, A., Mitra, B., Palanichamy, M. G., Baig, M., Chaudhuri, T. K., Shen, Y.-Y., Kong, Q.-P., Murphy, R. W., Yao, Y.-G., & Zhang, Y.-P. (2013). Chicken domestication: an updated perspective based on mitochondrial genomes. Heredity, 110(3), 277–282. https://doi.org/10.1038/hdy.2012.83
GU448581	B	B37	Miao, Y.-W., Peng, M.-S., Wu, G.-S., Ouyang, Y.-N., Yang, Z.-Y., Yu, N., Liang, J.-P., Pianchou, G., Beja-Pereira, A., Mitra, B., Palanichamy, M. G., Baig, M., Chaudhuri, T. K., Shen, Y.-Y., Kong, Q.-P., Murphy, R. W., Yao, Y.-G., & Zhang, Y.-P. (2013). Chicken domestication: an updated perspective based on mitochondrial genomes. Heredity, 110(3), 277–282. https://doi.org/10.1038/hdy.2012.83
GU448227	B	B38	Miao, Y.-W., Peng, M.-S., Wu, G.-S., Ouyang, Y.-N., Yang, Z.-Y., Yu, N., Liang, J.-P., Pianchou, G., Beja-Pereira, A., Mitra, B., Palanichamy, M. G., Baig, M., Chaudhuri, T. K., Shen, Y.-Y., Kong, Q.-P., Murphy, R. W., Yao, Y.-G., & Zhang, Y.-P. (2013). Chicken domestication: an updated perspective based on mitochondrial genomes. Heredity, 110(3), 277–282. https://doi.org/10.1038/hdy.2012.83
GU447949	B	B39	Miao, Y.-W., Peng, M.-S., Wu, G.-S., Ouyang, Y.-N., Yang, Z.-Y., Yu, N., Liang, J.-P., Pianchou, G., Beja-Pereira, A., Mitra, B., Palanichamy, M. G., Baig, M., Chaudhuri, T. K., Shen, Y.-Y., Kong, Q.-P., Murphy, R. W., Yao, Y.-G., & Zhang, Y.-P. (2013). Chicken domestication: an updated perspective based on mitochondrial genomes. Heredity, 110(3), 277–282. https://doi.org/10.1038/hdy.2012.83
GU447888	B	B40	Miao, Y.-W., Peng, M.-S., Wu, G.-S., Ouyang, Y.-N., Yang, Z.-Y., Yu, N., Liang, J.-P., Pianchou, G., Beja-Pereira, A., Mitra, B., Palanichamy, M. G., Baig, M., Chaudhuri, T. K., Shen, Y.-Y., Kong, Q.-P., Murphy, R. W., Yao, Y.-G., & Zhang, Y.-P. (2013). Chicken domestication: an updated perspective based on mitochondrial genomes. Heredity, 110(3), 277–282. https://doi.org/10.1038/hdy.2012.83

GU448134	B	B41	Miao, Y.-W., Peng, M.-S., Wu, G.-S., Ouyang, Y.-N., Yang, Z.-Y., Yu, N., Liang, J.-P., Pianchou, G., Beja-Pereira, A., Mitra, B., Palanichamy, M. G., Baig, M., Chaudhuri, T. K., Shen, Y.-Y., Kong, Q.-P., Murphy, R. W., Yao, Y.-G., & Zhang, Y.-P. (2013). Chicken domestication: an updated perspective based on mitochondrial genomes. Heredity, 110(3), 277–282. https://doi.org/10.1038/hdy.2012.83
GU447608	B	B42	Miao, Y.-W., Peng, M.-S., Wu, G.-S., Ouyang, Y.-N., Yang, Z.-Y., Yu, N., Liang, J.-P., Pianchou, G., Beja-Pereira, A., Mitra, B., Palanichamy, M. G., Baig, M., Chaudhuri, T. K., Shen, Y.-Y., Kong, Q.-P., Murphy, R. W., Yao, Y.-G., & Zhang, Y.-P. (2013). Chicken domestication: an updated perspective based on mitochondrial genomes. Heredity, 110(3), 277–282. https://doi.org/10.1038/hdy.2012.83
EU199937	B	B43	Silva, P., Guan, X., Ho-Shing, O., Jones, J., Xu, J., Hui, D., Notter, D., & Smith, E. (2009). Mitochondrial DNA-based analysis of genetic variation and relatedness among Sri Lankan indigenous chickens and the Ceylon junglefowl (Gallus lafayetti). *Animal genetics* , *40* (1), 1–9. https://doi.org/10.1111/j.1365-2052.2008.01783.x
GU447707	B	B44	Miao, Y.-W., Peng, M.-S., Wu, G.-S., Ouyang, Y.-N., Yang, Z.-Y., Yu, N., Liang, J.-P., Pianchou, G., Beja-Pereira, A., Mitra, B., Palanichamy, M. G., Baig, M., Chaudhuri, T. K., Shen, Y.-Y., Kong, Q.-P., Murphy, R. W., Yao, Y.-G., & Zhang, Y.-P. (2013). Chicken domestication: an updated perspective based on mitochondrial genomes. Heredity, 110(3), 277–282. https://doi.org/10.1038/hdy.2012.83
DQ462557	B	B45	Song and Jiang. 2006 Direct Submission
DQ462570	B	B46	Song and Jiang. 2006 Direct Submission
GU448253	B	B47	Miao, Y.-W., Peng, M.-S., Wu, G.-S., Ouyang, Y.-N., Yang, Z.-Y., Yu, N., Liang, J.-P., Pianchou, G., Beja-Pereira, A., Mitra, B., Palanichamy, M. G., Baig, M., Chaudhuri, T. K., Shen, Y.-Y., Kong, Q.-P., Murphy, R. W., Yao, Y.-G., & Zhang, Y.-P. (2013). Chicken domestication: an updated perspective based on mitochondrial genomes. Heredity, 110(3), 277–282. https://doi.org/10.1038/hdy.2012.83
GU448640	B	B48	Miao, Y.-W., Peng, M.-S., Wu, G.-S., Ouyang, Y.-N., Yang, Z.-Y., Yu, N., Liang, J.-P., Pianchou, G., Beja-Pereira, A., Mitra, B., Palanichamy, M. G., Baig, M., Chaudhuri, T. K., Shen, Y.-Y., Kong, Q.-P., Murphy, R. W., Yao, Y.-G., & Zhang, Y.-P. (2013). Chicken domestication: an updated perspective based on mitochondrial genomes. Heredity, 110(3), 277–282. https://doi.org/10.1038/hdy.2012.83
GU448624	B	B49	Miao, Y.-W., Peng, M.-S., Wu, G.-S., Ouyang, Y.-N., Yang, Z.-Y., Yu, N., Liang, J.-P., Pianchou, G., Beja-Pereira, A., Mitra, B., Palanichamy, M. G., Baig, M., Chaudhuri, T. K., Shen, Y.-Y., Kong, Q.-P., Murphy, R. W., Yao, Y.-G., & Zhang, Y.-P. (2013). Chicken domestication: an updated perspective based on mitochondrial genomes. Heredity, 110(3), 277–282. https://doi.org/10.1038/hdy.2012.83
GU447573	B	B50	Miao, Y.-W., Peng, M.-S., Wu, G.-S., Ouyang, Y.-N., Yang, Z.-Y., Yu, N., Liang, J.-P., Pianchou, G., Beja-Pereira, A., Mitra, B., Palanichamy, M. G., Baig, M., Chaudhuri, T. K., Shen, Y.-Y., Kong, Q.-P., Murphy, R. W., Yao, Y.-G., & Zhang, Y.-P. (2013). Chicken domestication: an updated perspective based on mitochondrial genomes. Heredity, 110(3), 277–282. https://doi.org/10.1038/hdy.2012.83
GU448623	B	B51	Miao, Y.-W., Peng, M.-S., Wu, G.-S., Ouyang, Y.-N., Yang, Z.-Y., Yu, N., Liang, J.-P., Pianchou, G., Beja-Pereira, A., Mitra, B., Palanichamy, M. G., Baig, M., Chaudhuri, T. K., Shen, Y.-Y., Kong, Q.-P., Murphy, R. W., Yao, Y.-G., & Zhang, Y.-P. (2013). Chicken domestication: an updated perspective based on mitochondrial genomes. Heredity, 110(3), 277–282. https://doi.org/10.1038/hdy.2012.83
GU447571	B	B52	Miao, Y.-W., Peng, M.-S., Wu, G.-S., Ouyang, Y.-N., Yang, Z.-Y., Yu, N., Liang, J.-P., Pianchou, G., Beja-Pereira, A., Mitra, B., Palanichamy, M. G., Baig, M., Chaudhuri, T. K., Shen, Y.-Y., Kong, Q.-P., Murphy, R. W., Yao, Y.-G., & Zhang, Y.-P. (2013). Chicken domestication: an updated perspective based on mitochondrial genomes. Heredity, 110(3), 277–282. https://doi.org/10.1038/hdy.2012.83
GU448651	B	B53	Miao, Y.-W., Peng, M.-S., Wu, G.-S., Ouyang, Y.-N., Yang, Z.-Y., Yu, N., Liang, J.-P., Pianchou, G., Beja-Pereira, A., Mitra, B., Palanichamy, M. G., Baig, M., Chaudhuri, T. K., Shen, Y.-Y., Kong, Q.-P., Murphy, R. W., Yao, Y.-G., & Zhang, Y.-P. (2013). Chicken domestication: an updated perspective based on mitochondrial genomes. Heredity, 110(3), 277–282. https://doi.org/10.1038/hdy.2012.83
GU448722	B	B54	Miao, Y.-W., Peng, M.-S., Wu, G.-S., Ouyang, Y.-N., Yang, Z.-Y., Yu, N., Liang, J.-P., Pianchou, G., Beja-Pereira, A., Mitra, B., Palanichamy, M. G., Baig, M., Chaudhuri, T. K., Shen, Y.-Y., Kong, Q.-P., Murphy, R. W., Yao, Y.-G., & Zhang, Y.-P. (2013). Chicken domestication: an updated perspective based on mitochondrial genomes. Heredity, 110(3), 277–282. https://doi.org/10.1038/hdy.2012.83
GU447570	B	B55	Miao, Y.-W., Peng, M.-S., Wu, G.-S., Ouyang, Y.-N., Yang, Z.-Y., Yu, N., Liang, J.-P., Pianchou, G., Beja-Pereira, A., Mitra, B., Palanichamy, M. G., Baig, M., Chaudhuri, T. K., Shen, Y.-Y., Kong, Q.-P., Murphy, R. W., Yao, Y.-G., & Zhang, Y.-P. (2013). Chicken domestication: an updated perspective based on mitochondrial genomes. Heredity, 110(3), 277–282. https://doi.org/10.1038/hdy.2012.83
GU447825	B	B56	Miao, Y.-W., Peng, M.-S., Wu, G.-S., Ouyang, Y.-N., Yang, Z.-Y., Yu, N., Liang, J.-P., Pianchou, G., Beja-Pereira, A., Mitra, B., Palanichamy, M. G., Baig, M., Chaudhuri, T. K., Shen, Y.-Y., Kong, Q.-P., Murphy, R. W., Yao, Y.-G., & Zhang, Y.-P. (2013). Chicken domestication: an updated perspective based on mitochondrial genomes. Heredity, 110(3), 277–282. https://doi.org/10.1038/hdy.2012.83
GU448029	B	B57	Miao, Y.-W., Peng, M.-S., Wu, G.-S., Ouyang, Y.-N., Yang, Z.-Y., Yu, N., Liang, J.-P., Pianchou, G., Beja-Pereira, A., Mitra, B., Palanichamy, M. G., Baig, M., Chaudhuri, T. K., Shen, Y.-Y., Kong, Q.-P., Murphy, R. W., Yao, Y.-G., & Zhang, Y.-P. (2013). Chicken domestication: an updated perspective based on mitochondrial genomes. Heredity, 110(3), 277–282. https://doi.org/10.1038/hdy.2012.83
GU448925	B	B58	Miao, Y.-W., Peng, M.-S., Wu, G.-S., Ouyang, Y.-N., Yang, Z.-Y., Yu, N., Liang, J.-P., Pianchou, G., Beja-Pereira, A., Mitra, B., Palanichamy, M. G., Baig, M., Chaudhuri, T. K., Shen, Y.-Y., Kong, Q.-P., Murphy, R. W., Yao, Y.-G., & Zhang, Y.-P. (2013). Chicken domestication: an updated perspective based on mitochondrial genomes. Heredity, 110(3), 277–282. https://doi.org/10.1038/hdy.2012.83
GU449044	B	B59	Miao, Y.-W., Peng, M.-S., Wu, G.-S., Ouyang, Y.-N., Yang, Z.-Y., Yu, N., Liang, J.-P., Pianchou, G., Beja-Pereira, A., Mitra, B., Palanichamy, M. G., Baig, M., Chaudhuri, T. K., Shen, Y.-Y., Kong, Q.-P., Murphy, R. W., Yao, Y.-G., & Zhang, Y.-P. (2013). Chicken domestication: an updated perspective based on mitochondrial genomes. Heredity, 110(3), 277–282. https://doi.org/10.1038/hdy.2012.83
HM462168	B	B60	Berthouly-Salazar, C., Rognon, X., Van, T., Gély, M., Chi, C. V., Tixier-Boichard, M., Bed'Hom, B., Bruneau, N., Verrier, E., Maillard, J. C., & Michaux, J. R. (2010). Vietnamese chickens: a gate towards Asian genetic diversity. *BMC genetics* , *11* , 53. https://doi.org/10.1186/1471-2156-11-53
HM462140	B	B61	Berthouly-Salazar, C., Rognon, X., Van, T., Gély, M., Chi, C. V., Tixier-Boichard, M., Bed'Hom, B., Bruneau, N., Verrier, E., Maillard, J. C., & Michaux, J. R. (2010). Vietnamese chickens: a gate towards Asian genetic diversity. *BMC genetics* , *11* , 53. https://doi.org/10.1186/1471-2156-11-54
HM462143	B	B62	Berthouly-Salazar, C., Rognon, X., Van, T., Gély, M., Chi, C. V., Tixier-Boichard, M., Bed'Hom, B., Bruneau, N., Verrier, E., Maillard, J. C., & Michaux, J. R. (2010). Vietnamese chickens: a gate towards Asian genetic diversity. *BMC genetics* , *11* , 53. https://doi.org/10.1186/1471-2156-11-55
HM462095	B	B63	Berthouly-Salazar, C., Rognon, X., Van, T., Gély, M., Chi, C. V., Tixier-Boichard, M., Bed'Hom, B., Bruneau, N., Verrier, E., Maillard, J. C., & Michaux, J. R. (2010). Vietnamese chickens: a gate towards Asian genetic diversity. *BMC genetics* , *11* , 53. https://doi.org/10.1186/1471-2156-11-56
HM462085	B	B64	Berthouly-Salazar, C., Rognon, X., Van, T., Gély, M., Chi, C. V., Tixier-Boichard, M., Bed'Hom, B., Bruneau, N., Verrier, E., Maillard, J. C., & Michaux, J. R. (2010). Vietnamese chickens: a gate towards Asian genetic diversity. *BMC genetics* , *11* , 53. https://doi.org/10.1186/1471-2156-11-57
GU448212	B	B67	Miao, Y.-W., Peng, M.-S., Wu, G.-S., Ouyang, Y.-N., Yang, Z.-Y., Yu, N., Liang, J.-P., Pianchou, G., Beja-Pereira, A., Mitra, B., Palanichamy, M. G., Baig, M., Chaudhuri, T. K., Shen, Y.-Y., Kong, Q.-P., Murphy, R. W., Yao, Y.-G., & Zhang, Y.-P. (2013). Chicken domestication: an updated perspective based on mitochondrial genomes. Heredity, 110(3), 277–282. https://doi.org/10.1038/hdy.2012.83
GU447595	B	B68	Miao, Y.-W., Peng, M.-S., Wu, G.-S., Ouyang, Y.-N., Yang, Z.-Y., Yu, N., Liang, J.-P., Pianchou, G., Beja-Pereira, A., Mitra, B., Palanichamy, M. G., Baig, M., Chaudhuri, T. K., Shen, Y.-Y., Kong, Q.-P., Murphy, R. W., Yao, Y.-G., & Zhang, Y.-P. (2013). Chicken domestication: an updated perspective based on mitochondrial genomes. Heredity, 110(3), 277–282. https://doi.org/10.1038/hdy.2012.83
GU447887	C1	C01	Miao, Y.-W., Peng, M.-S., Wu, G.-S., Ouyang, Y.-N., Yang, Z.-Y., Yu, N., Liang, J.-P., Pianchou, G., Beja-Pereira, A., Mitra, B., Palanichamy, M. G., Baig, M., Chaudhuri, T. K., Shen, Y.-Y., Kong, Q.-P., Murphy, R. W., Yao, Y.-G., & Zhang, Y.-P. (2013). Chicken domestication: an updated perspective based on mitochondrial genomes. Heredity, 110(3), 277–282. https://doi.org/10.1038/hdy.2012.83
GU447798	C1	C02	Miao, Y.-W., Peng, M.-S., Wu, G.-S., Ouyang, Y.-N., Yang, Z.-Y., Yu, N., Liang, J.-P., Pianchou, G., Beja-Pereira, A., Mitra, B., Palanichamy, M. G., Baig, M., Chaudhuri, T. K., Shen, Y.-Y., Kong, Q.-P., Murphy, R. W., Yao, Y.-G., & Zhang, Y.-P. (2013). Chicken domestication: an updated perspective based on mitochondrial genomes. Heredity, 110(3), 277–282. https://doi.org/10.1038/hdy.2012.83
GU449046	C1	C03	Miao, Y.-W., Peng, M.-S., Wu, G.-S., Ouyang, Y.-N., Yang, Z.-Y., Yu, N., Liang, J.-P., Pianchou, G., Beja-Pereira, A., Mitra, B., Palanichamy, M. G., Baig, M., Chaudhuri, T. K., Shen, Y.-Y., Kong, Q.-P., Murphy, R. W., Yao, Y.-G., & Zhang, Y.-P. (2013). Chicken domestication: an updated perspective based on mitochondrial genomes. Heredity, 110(3), 277–282. https://doi.org/10.1038/hdy.2012.83
GU447591	C1	C04	Miao, Y.-W., Peng, M.-S., Wu, G.-S., Ouyang, Y.-N., Yang, Z.-Y., Yu, N., Liang, J.-P., Pianchou, G., Beja-Pereira, A., Mitra, B., Palanichamy, M. G., Baig, M., Chaudhuri, T. K., Shen, Y.-Y., Kong, Q.-P., Murphy, R. W., Yao, Y.-G., & Zhang, Y.-P. (2013). Chicken domestication: an updated perspective based on mitochondrial genomes. Heredity, 110(3), 277–282. https://doi.org/10.1038/hdy.2012.83
GU448028	C1	C05	Miao, Y.-W., Peng, M.-S., Wu, G.-S., Ouyang, Y.-N., Yang, Z.-Y., Yu, N., Liang, J.-P., Pianchou, G., Beja-Pereira, A., Mitra, B., Palanichamy, M. G., Baig, M., Chaudhuri, T. K., Shen, Y.-Y., Kong, Q.-P., Murphy, R. W., Yao, Y.-G., & Zhang, Y.-P. (2013). Chicken domestication: an updated perspective based on mitochondrial genomes. Heredity, 110(3), 277–282. https://doi.org/10.1038/hdy.2012.83
GU447346	C1	C06	Miao, Y.-W., Peng, M.-S., Wu, G.-S., Ouyang, Y.-N., Yang, Z.-Y., Yu, N., Liang, J.-P., Pianchou, G., Beja-Pereira, A., Mitra, B., Palanichamy, M. G., Baig, M., Chaudhuri, T. K., Shen, Y.-Y., Kong, Q.-P., Murphy, R. W., Yao, Y.-G., & Zhang, Y.-P. (2013). Chicken domestication: an updated perspective based on mitochondrial genomes. Heredity, 110(3), 277–282. https://doi.org/10.1038/hdy.2012.83
AB007745	C1	C07	Miyake 2000 Direct Submission
AF512329	C1	C08	Liu, Y. P., Wu, G. S., Yao, Y. G., Miao, Y. W., Luikart, G., Baig, M., Beja-Pereira, A., Ding, Z. L., Palanichamy, M. G., & Zhang, Y. P. (2006). Multiple maternal origins of chickens: out of the Asian jungles. *Molecular phylogenetics and evolution* , *38* (1), 12–19. https://doi.org/10.1016/j.ympev.2005.09.014
GU448570	C1	C09	Miao, Y.-W., Peng, M.-S., Wu, G.-S., Ouyang, Y.-N., Yang, Z.-Y., Yu, N., Liang, J.-P., Pianchou, G., Beja-Pereira, A., Mitra, B., Palanichamy, M. G., Baig, M., Chaudhuri, T. K., Shen, Y.-Y., Kong, Q.-P., Murphy, R. W., Yao, Y.-G., & Zhang, Y.-P. (2013). Chicken domestication: an updated perspective based on mitochondrial genomes. Heredity, 110(3), 277–282. https://doi.org/10.1038/hdy.2012.83
AB098699	C1	C10	Komiyama, T., Ikeo, K., & Gojobori, T. (2003). Where is the origin of the Japanese gamecocks?. *Gene* , *317* (1-2), 195–202. https://doi.org/10.1016/s0378-1119(03)00703-0
AB098697	C1	C11	Komiyama, T., Ikeo, K., & Gojobori, T. (2003). Where is the origin of the Japanese gamecocks?. *Gene* , *317* (1-2), 195–202. https://doi.org/10.1016/s0378-1119(03)00703-1
AB098644	C1	C12	Komiyama, T., Ikeo, K., & Gojobori, T. (2003). Where is the origin of the Japanese gamecocks?. *Gene* , *317* (1-2), 195–202. https://doi.org/10.1016/s0378-1119(03)00703-2
AB098649	C1	C13	Komiyama, T., Ikeo, K., & Gojobori, T. (2003). Where is the origin of the Japanese gamecocks?. *Gene* , *317* (1-2), 195–202. https://doi.org/10.1016/s0378-1119(03)00703-3
AB098637	C1	C14	Komiyama, T., Ikeo, K., & Gojobori, T. (2003). Where is the origin of the Japanese gamecocks?. *Gene* , *317* (1-2), 195–202. https://doi.org/10.1016/s0378-1119(03)00703-4

AB098642	C1	C15	Komiyama, T., Ikeo, K., & Gojobori, T. (2003). Where is the origin of the Japanese gamecocks?. *Gene* , *317* (1-2), 195–202. https://doi.org/10.1016/s0378-1119(03)00703-5
AB098636	C1	C16	Komiyama, T., Ikeo, K., & Gojobori, T. (2003). Where is the origin of the Japanese gamecocks?. *Gene* , *317* (1-2), 195–202. https://doi.org/10.1016/s0378-1119(03)00703-6
AB098648	C1	C17	Komiyama, T., Ikeo, K., & Gojobori, T. (2003). Where is the origin of the Japanese gamecocks?. *Gene* , *317* (1-2), 195–202. https://doi.org/10.1016/s0378-1119(03)00703-7
AB114072	C1	C18	Komiyama, T., Ikeo, K., & Gojobori, T. (2004). The evolutionary origin of long-crowing chicken: its evolutionary relationship with fighting cocks disclosed by the mtDNA sequence analysis. *Gene* , *333* , 91–99. https://doi.org/10.1016/j.gene.2004.02.035
AB114075	C1	C19	Komiyama, T., Ikeo, K., & Gojobori, T. (2004). The evolutionary origin of long-crowing chicken: its evolutionary relationship with fighting cocks disclosed by the mtDNA sequence analysis. *Gene* , *333* , 91–99. https://doi.org/10.1016/j.gene.2004.02.035
GU447330	C1	C20	Miao, Y.-W., Peng, M.-S., Wu, G.-S., Ouyang, Y.-N., Yang, Z.-Y., Yu, N., Liang, J.-P., Pianchou, G., Beja-Pereira, A., Mitra, B., Palanichamy, M. G., Baig, M., Chaudhuri, T. K., Shen, Y.-Y., Kong, Q.-P., Murphy, R. W., Yao, Y.-G., & Zhang, Y.-P. (2013). Chicken domestication: an updated perspective based on mitochondrial genomes. Heredity, 110(3), 277–282. https://doi.org/10.1038/hdy.2012.83
GU447441	C1	C21	Miao, Y.-W., Peng, M.-S., Wu, G.-S., Ouyang, Y.-N., Yang, Z.-Y., Yu, N., Liang, J.-P., Pianchou, G., Beja-Pereira, A., Mitra, B., Palanichamy, M. G., Baig, M., Chaudhuri, T. K., Shen, Y.-Y., Kong, Q.-P., Murphy, R. W., Yao, Y.-G., & Zhang, Y.-P. (2013). Chicken domestication: an updated perspective based on mitochondrial genomes. Heredity, 110(3), 277–282. https://doi.org/10.1038/hdy.2012.83
GU448891	C1	C22	Miao, Y.-W., Peng, M.-S., Wu, G.-S., Ouyang, Y.-N., Yang, Z.-Y., Yu, N., Liang, J.-P., Pianchou, G., Beja-Pereira, A., Mitra, B., Palanichamy, M. G., Baig, M., Chaudhuri, T. K., Shen, Y.-Y., Kong, Q.-P., Murphy, R. W., Yao, Y.-G., & Zhang, Y.-P. (2013). Chicken domestication: an updated perspective based on mitochondrial genomes. Heredity, 110(3), 277–282. https://doi.org/10.1038/hdy.2012.83
GU447809	C1	C23	Miao, Y.-W., Peng, M.-S., Wu, G.-S., Ouyang, Y.-N., Yang, Z.-Y., Yu, N., Liang, J.-P., Pianchou, G., Beja-Pereira, A., Mitra, B., Palanichamy, M. G., Baig, M., Chaudhuri, T. K., Shen, Y.-Y., Kong, Q.-P., Murphy, R. W., Yao, Y.-G., & Zhang, Y.-P. (2013). Chicken domestication: an updated perspective based on mitochondrial genomes. Heredity, 110(3), 277–282. https://doi.org/10.1038/hdy.2012.83
GU447681	C1	C24	Miao, Y.-W., Peng, M.-S., Wu, G.-S., Ouyang, Y.-N., Yang, Z.-Y., Yu, N., Liang, J.-P., Pianchou, G., Beja-Pereira, A., Mitra, B., Palanichamy, M. G., Baig, M., Chaudhuri, T. K., Shen, Y.-Y., Kong, Q.-P., Murphy, R. W., Yao, Y.-G., & Zhang, Y.-P. (2013). Chicken domestication: an updated perspective based on mitochondrial genomes. Heredity, 110(3), 277–282. https://doi.org/10.1038/hdy.2012.83
GU448783	C1	C25	Miao, Y.-W., Peng, M.-S., Wu, G.-S., Ouyang, Y.-N., Yang, Z.-Y., Yu, N., Liang, J.-P., Pianchou, G., Beja-Pereira, A., Mitra, B., Palanichamy, M. G., Baig, M., Chaudhuri, T. K., Shen, Y.-Y., Kong, Q.-P., Murphy, R. W., Yao, Y.-G., & Zhang, Y.-P. (2013). Chicken domestication: an updated perspective based on mitochondrial genomes. Heredity, 110(3), 277–282. https://doi.org/10.1038/hdy.2012.83
GU447428	C1	C26	Miao, Y.-W., Peng, M.-S., Wu, G.-S., Ouyang, Y.-N., Yang, Z.-Y., Yu, N., Liang, J.-P., Pianchou, G., Beja-Pereira, A., Mitra, B., Palanichamy, M. G., Baig, M., Chaudhuri, T. K., Shen, Y.-Y., Kong, Q.-P., Murphy, R. W., Yao, Y.-G., & Zhang, Y.-P. (2013). Chicken domestication: an updated perspective based on mitochondrial genomes. Heredity, 110(3), 277–282. https://doi.org/10.1038/hdy.2012.83
GU448196	C1	C27	Miao, Y.-W., Peng, M.-S., Wu, G.-S., Ouyang, Y.-N., Yang, Z.-Y., Yu, N., Liang, J.-P., Pianchou, G., Beja-Pereira, A., Mitra, B., Palanichamy, M. G., Baig, M., Chaudhuri, T. K., Shen, Y.-Y., Kong, Q.-P., Murphy, R. W., Yao, Y.-G., & Zhang, Y.-P. (2013). Chicken domestication: an updated perspective based on mitochondrial genomes. Heredity, 110(3), 277–282. https://doi.org/10.1038/hdy.2012.83
GU447550	C1	C28	Miao, Y.-W., Peng, M.-S., Wu, G.-S., Ouyang, Y.-N., Yang, Z.-Y., Yu, N., Liang, J.-P., Pianchou, G., Beja-Pereira, A., Mitra, B., Palanichamy, M. G., Baig, M., Chaudhuri, T. K., Shen, Y.-Y., Kong, Q.-P., Murphy, R. W., Yao, Y.-G., & Zhang, Y.-P. (2013). Chicken domestication: an updated perspective based on mitochondrial genomes. Heredity, 110(3), 277–282. https://doi.org/10.1038/hdy.2012.83
GU448975	C1	C29	Miao, Y.-W., Peng, M.-S., Wu, G.-S., Ouyang, Y.-N., Yang, Z.-Y., Yu, N., Liang, J.-P., Pianchou, G., Beja-Pereira, A., Mitra, B., Palanichamy, M. G., Baig, M., Chaudhuri, T. K., Shen, Y.-Y., Kong, Q.-P., Murphy, R. W., Yao, Y.-G., & Zhang, Y.-P. (2013). Chicken domestication: an updated perspective based on mitochondrial genomes. Heredity, 110(3), 277–282. https://doi.org/10.1038/hdy.2012.83
GU448828	C1	C30	Miao, Y.-W., Peng, M.-S., Wu, G.-S., Ouyang, Y.-N., Yang, Z.-Y., Yu, N., Liang, J.-P., Pianchou, G., Beja-Pereira, A., Mitra, B., Palanichamy, M. G., Baig, M., Chaudhuri, T. K., Shen, Y.-Y., Kong, Q.-P., Murphy, R. W., Yao, Y.-G., & Zhang, Y.-P. (2013). Chicken domestication: an updated perspective based on mitochondrial genomes. Heredity, 110(3), 277–282. https://doi.org/10.1038/hdy.2012.83
GU448200	C1	C31	Miao, Y.-W., Peng, M.-S., Wu, G.-S., Ouyang, Y.-N., Yang, Z.-Y., Yu, N., Liang, J.-P., Pianchou, G., Beja-Pereira, A., Mitra, B., Palanichamy, M. G., Baig, M., Chaudhuri, T. K., Shen, Y.-Y., Kong, Q.-P., Murphy, R. W., Yao, Y.-G., & Zhang, Y.-P. (2013). Chicken domestication: an updated perspective based on mitochondrial genomes. Heredity, 110(3), 277–282. https://doi.org/10.1038/hdy.2012.83
GU448015	C1	C32	Miao, Y.-W., Peng, M.-S., Wu, G.-S., Ouyang, Y.-N., Yang, Z.-Y., Yu, N., Liang, J.-P., Pianchou, G., Beja-Pereira, A., Mitra, B., Palanichamy, M. G., Baig, M., Chaudhuri, T. K., Shen, Y.-Y., Kong, Q.-P., Murphy, R. W., Yao, Y.-G., & Zhang, Y.-P. (2013). Chicken domestication: an updated perspective based on mitochondrial genomes. Heredity, 110(3), 277–282. https://doi.org/10.1038/hdy.2012.83
AB268538	C1	C33	Oka, T., Ino, Y., Nomura, K., Kawashima, S., Kuwayama, T., Hanada, H., Amano, T., Takada, M., Takahata, N., Hayashi, Y., & Akishinonomiya, F. (2007). Analysis of mtDNA sequences shows Japanese native chickens have multiple origins. *Animal genetics* , *38* (3), 287–293. https://doi.org/10.1111/j.1365-2052.2007.01604.x
GU448493	C1	C34	Miao, Y.-W., Peng, M.-S., Wu, G.-S., Ouyang, Y.-N., Yang, Z.-Y., Yu, N., Liang, J.-P., Pianchou, G., Beja-Pereira, A., Mitra, B., Palanichamy, M. G., Baig, M., Chaudhuri, T. K., Shen, Y.-Y., Kong, Q.-P., Murphy, R. W., Yao, Y.-G., & Zhang, Y.-P. (2013). Chicken domestication: an updated perspective based on mitochondrial genomes. Heredity, 110(3), 277–282. https://doi.org/10.1038/hdy.2012.83
GU448278	C1	C35	Miao, Y.-W., Peng, M.-S., Wu, G.-S., Ouyang, Y.-N., Yang, Z.-Y., Yu, N., Liang, J.-P., Pianchou, G., Beja-Pereira, A., Mitra, B., Palanichamy, M. G., Baig, M., Chaudhuri, T. K., Shen, Y.-Y., Kong, Q.-P., Murphy, R. W., Yao, Y.-G., & Zhang, Y.-P. (2013). Chicken domestication: an updated perspective based on mitochondrial genomes. Heredity, 110(3), 277–282. https://doi.org/10.1038/hdy.2012.83
GU448204	C1	C36	Miao, Y.-W., Peng, M.-S., Wu, G.-S., Ouyang, Y.-N., Yang, Z.-Y., Yu, N., Liang, J.-P., Pianchou, G., Beja-Pereira, A., Mitra, B., Palanichamy, M. G., Baig, M., Chaudhuri, T. K., Shen, Y.-Y., Kong, Q.-P., Murphy, R. W., Yao, Y.-G., & Zhang, Y.-P. (2013). Chicken domestication: an updated perspective based on mitochondrial genomes. Heredity, 110(3), 277–282. https://doi.org/10.1038/hdy.2012.83
AB263962	C1	C37	Wada et al. 2008 Direct Submission
GU447590	C1	C38	Miao, Y.-W., Peng, M.-S., Wu, G.-S., Ouyang, Y.-N., Yang, Z.-Y., Yu, N., Liang, J.-P., Pianchou, G., Beja-Pereira, A., Mitra, B., Palanichamy, M. G., Baig, M., Chaudhuri, T. K., Shen, Y.-Y., Kong, Q.-P., Murphy, R. W., Yao, Y.-G., & Zhang, Y.-P. (2013). Chicken domestication: an updated perspective based on mitochondrial genomes. Heredity, 110(3), 277–282. https://doi.org/10.1038/hdy.2012.83
GU447762	C1	C39	Miao, Y.-W., Peng, M.-S., Wu, G.-S., Ouyang, Y.-N., Yang, Z.-Y., Yu, N., Liang, J.-P., Pianchou, G., Beja-Pereira, A., Mitra, B., Palanichamy, M. G., Baig, M., Chaudhuri, T. K., Shen, Y.-Y., Kong, Q.-P., Murphy, R. W., Yao, Y.-G., & Zhang, Y.-P. (2013). Chicken domestication: an updated perspective based on mitochondrial genomes. Heredity, 110(3), 277–282. https://doi.org/10.1038/hdy.2012.83
EU095094	C1	C40	Mwacharo et al. 2008 Direct Submission
AB263960	C1	C41	Wada et al. 2008 Direct Submission
GU448951	C3	C42	Miao, Y.-W., Peng, M.-S., Wu, G.-S., Ouyang, Y.-N., Yang, Z.-Y., Yu, N., Liang, J.-P., Pianchou, G., Beja-Pereira, A., Mitra, B., Palanichamy, M. G., Baig, M., Chaudhuri, T. K., Shen, Y.-Y., Kong, Q.-P., Murphy, R. W., Yao, Y.-G., & Zhang, Y.-P. (2013). Chicken domestication: an updated perspective based on mitochondrial genomes. Heredity, 110(3), 277–282. https://doi.org/10.1038/hdy.2012.83
GU447583	C3	C43	Miao, Y.-W., Peng, M.-S., Wu, G.-S., Ouyang, Y.-N., Yang, Z.-Y., Yu, N., Liang, J.-P., Pianchou, G., Beja-Pereira, A., Mitra, B., Palanichamy, M. G., Baig, M., Chaudhuri, T. K., Shen, Y.-Y., Kong, Q.-P., Murphy, R. W., Yao, Y.-G., & Zhang, Y.-P. (2013). Chicken domestication: an updated perspective based on mitochondrial genomes. Heredity, 110(3), 277–282. https://doi.org/10.1038/hdy.2012.83
GU448724	C2	C44	Miao, Y.-W., Peng, M.-S., Wu, G.-S., Ouyang, Y.-N., Yang, Z.-Y., Yu, N., Liang, J.-P., Pianchou, G., Beja-Pereira, A., Mitra, B., Palanichamy, M. G., Baig, M., Chaudhuri, T. K., Shen, Y.-Y., Kong, Q.-P., Murphy, R. W., Yao, Y.-G., & Zhang, Y.-P. (2013). Chicken domestication: an updated perspective based on mitochondrial genomes. Heredity, 110(3), 277–282. https://doi.org/10.1038/hdy.2012.83
EU847751	C	C45	Kanginakudru, S., Metta, M., Jakati, R. D., & Nagaraju, J. (2008). Genetic evidence from Indian red jungle fowl corroborates multiple domestication of modern day chicken. *BMC evolutionary biology* , *8* , 174. https://doi.org/10.1186/1471-2148-8-174
EU847784	C	C46	Kanginakudru, S., Metta, M., Jakati, R. D., & Nagaraju, J. (2008). Genetic evidence from Indian red jungle fowl corroborates multiple domestication of modern day chicken. *BMC evolutionary biology* , *8* , 174. https://doi.org/10.1186/1471-2148-8-174
EU847789	C	C47	Kanginakudru, S., Metta, M., Jakati, R. D., & Nagaraju, J. (2008). Genetic evidence from Indian red jungle fowl corroborates multiple domestication of modern day chicken. *BMC evolutionary biology* , *8* , 174. https://doi.org/10.1186/1471-2148-8-174
EU847781	C	C48	Kanginakudru, S., Metta, M., Jakati, R. D., & Nagaraju, J. (2008). Genetic evidence from Indian red jungle fowl corroborates multiple domestication of modern day chicken. *BMC evolutionary biology* , *8* , 174. https://doi.org/10.1186/1471-2148-8-174
EU847782	C	C49	Kanginakudru, S., Metta, M., Jakati, R. D., & Nagaraju, J. (2008). Genetic evidence from Indian red jungle fowl corroborates multiple domestication of modern day chicken. *BMC evolutionary biology* , *8* , 174. https://doi.org/10.1186/1471-2148-8-174
EU847772	C	C50	Kanginakudru, S., Metta, M., Jakati, R. D., & Nagaraju, J. (2008). Genetic evidence from Indian red jungle fowl corroborates multiple domestication of modern day chicken. *BMC evolutionary biology* , *8* , 174. https://doi.org/10.1186/1471-2148-8-174
EU847787	C	C51	Kanginakudru, S., Metta, M., Jakati, R. D., & Nagaraju, J. (2008). Genetic evidence from Indian red jungle fowl corroborates multiple domestication of modern day chicken. *BMC evolutionary biology* , *8* , 174. https://doi.org/10.1186/1471-2148-8-174
EU847749	C	C52	Kanginakudru, S., Metta, M., Jakati, R. D., & Nagaraju, J. (2008). Genetic evidence from Indian red jungle fowl corroborates multiple domestication of modern day chicken. *BMC evolutionary biology* , *8* , 174. https://doi.org/10.1186/1471-2148-8-174
EU847795	C	C53	Kanginakudru, S., Metta, M., Jakati, R. D., & Nagaraju, J. (2008). Genetic evidence from Indian red jungle fowl corroborates multiple domestication of modern day chicken. *BMC evolutionary biology* , *8* , 174. https://doi.org/10.1186/1471-2148-8-174
EU847791	C	C54	Kanginakudru, S., Metta, M., Jakati, R. D., & Nagaraju, J. (2008). Genetic evidence from Indian red jungle fowl corroborates multiple domestication of modern day chicken. *BMC evolutionary biology* , *8* , 174. https://doi.org/10.1186/1471-2148-8-174

EU847796	C	C55	Kanginakudru, S., Metta, M., Jakati, R. D., & Nagaraju, J. (2008). Genetic evidence from Indian red jungle fowl corroborates multiple domestication of modern day chicken. *BMC evolutionary biology* , *8* , 174. https://doi.org/10.1186/1471-2148-8-174
EU847750	C	C56	Kanginakudru, S., Metta, M., Jakati, R. D., & Nagaraju, J. (2008). Genetic evidence from Indian red jungle fowl corroborates multiple domestication of modern day chicken. *BMC evolutionary biology* , *8* , 174. https://doi.org/10.1186/1471-2148-8-174
EU847800	C	C57	Kanginakudru, S., Metta, M., Jakati, R. D., & Nagaraju, J. (2008). Genetic evidence from Indian red jungle fowl corroborates multiple domestication of modern day chicken. *BMC evolutionary biology* , *8* , 174. https://doi.org/10.1186/1471-2148-8-174
GQ293096	C	C58	Ahmed et al. 2009 Direct Submission
HM462202	C	C60	Berthouly-Salazar, C., Rognon, X., Van, T., Gély, M., Chi, C. V., Tixier-Boichard, M., Bed'Hom, B., Bruneau, N., Verrier, E., Maillard, J. C., & Michaux, J. R. (2010). Vietnamese chickens: a gate towards Asian genetic diversity. *BMC genetics* , *11* , 53. https://doi.org/10.1186/1471-2156-11-53
HM462206	C	C61	Berthouly-Salazar, C., Rognon, X., Van, T., Gély, M., Chi, C. V., Tixier-Boichard, M., Bed'Hom, B., Bruneau, N., Verrier, E., Maillard, J. C., & Michaux, J. R. (2010). Vietnamese chickens: a gate towards Asian genetic diversity. *BMC genetics* , *11* , 53. https://doi.org/10.1186/1471-2156-11-53
HM462208	C	C62	Berthouly-Salazar, C., Rognon, X., Van, T., Gély, M., Chi, C. V., Tixier-Boichard, M., Bed'Hom, B., Bruneau, N., Verrier, E., Maillard, J. C., & Michaux, J. R. (2010). Vietnamese chickens: a gate towards Asian genetic diversity. *BMC genetics* , *11* , 53. https://doi.org/10.1186/1471-2156-11-53
EU199944	D	D01	Silva, P., Guan, X., Ho-Shing, O., Jones, J., Xu, J., Hui, D., Notter, D., & Smith, E. (2009). Mitochondrial DNA-based analysis of genetic variation and relatedness among Sri Lankan indigenous chickens and the Ceylon junglefowl (Gallus lafayetti). *Animal genetics* , *40* (1), 1–9. https://doi.org/10.1111/j.1365-2052.2008.01783.x
GU448859	D	D02	Miao, Y.-W., Peng, M.-S., Wu, G.-S., Ouyang, Y.-N., Yang, Z.-Y., Yu, N., Liang, J.-P., Pianchou, G., Beja-Pereira, A., Mitra, B., Palanichamy, M. G., Baig, M., Chaudhuri, T. K., Shen, Y.-Y., Kong, Q.-P., Murphy, R. W., Yao, Y.-G., & Zhang, Y.-P. (2013). Chicken domestication: an updated perspective based on mitochondrial genomes. Heredity, 110(3), 277–282. https://doi.org/10.1038/hdy.2012.83
AB009441	D	D03	Miyake 2000 Direct Submission
AB007741	D	D04	Miyake 2000 Direct Submission
D82900	D	D05	Fumihito, A., Miyake, T., Takada, M., Shingu, R., Endo, T., Gojobori, T., Kondo, N., & Ohno, S. (1996). Monophyletic origin and unique dispersal patterns of domestic fowls. *Proceedings of the National Academy of Sciences of the United States of America* , *93* (13), 6792–6795. https://doi.org/10.1073/pnas.93.13.6792
GU448997	D	D06	Miao, Y.-W., Peng, M.-S., Wu, G.-S., Ouyang, Y.-N., Yang, Z.-Y., Yu, N., Liang, J.-P., Pianchou, G., Beja-Pereira, A., Mitra, B., Palanichamy, M. G., Baig, M., Chaudhuri, T. K., Shen, Y.-Y., Kong, Q.-P., Murphy, R. W., Yao, Y.-G., & Zhang, Y.-P. (2013). Chicken domestication: an updated perspective based on mitochondrial genomes. Heredity, 110(3), 277–282. https://doi.org/10.1038/hdy.2012.83
AB009437	D	D07	Miyake 2000 Direct Submission
D82917	D	D08	Fumihito, A., Miyake, T., Takada, M., Shingu, R., Endo, T., Gojobori, T., Kondo, N., & Ohno, S. (1996). Monophyletic origin and unique dispersal patterns of domestic fowls. *Proceedings of the National Academy of Sciences of the United States of America* , *93* (13), 6792–6795. https://doi.org/10.1073/pnas.93.13.6792
D82918	D	D09	Fumihito, A., Miyake, T., Takada, M., Shingu, R., Endo, T., Gojobori, T., Kondo, N., & Ohno, S. (1996). Monophyletic origin and unique dispersal patterns of domestic fowls. *Proceedings of the National Academy of Sciences of the United States of America* , *93* (13), 6792–6795. https://doi.org/10.1073/pnas.93.13.6792
GU448713	D	D10	Miao, Y.-W., Peng, M.-S., Wu, G.-S., Ouyang, Y.-N., Yang, Z.-Y., Yu, N., Liang, J.-P., Pianchou, G., Beja-Pereira, A., Mitra, B., Palanichamy, M. G., Baig, M., Chaudhuri, T. K., Shen, Y.-Y., Kong, Q.-P., Murphy, R. W., Yao, Y.-G., & Zhang, Y.-P. (2013). Chicken domestication: an updated perspective based on mitochondrial genomes. Heredity, 110(3), 277–282. https://doi.org/10.1038/hdy.2012.83
AB009432	D	D11	Miyake 2000 Direct Submission
D82902	D	D12	Fumihito, A., Miyake, T., Takada, M., Shingu, R., Endo, T., Gojobori, T., Kondo, N., & Ohno, S. (1996). Monophyletic origin and unique dispersal patterns of domestic fowls. *Proceedings of the National Academy of Sciences of the United States of America* , *93* (13), 6792–6795. https://doi.org/10.1073/pnas.93.13.6792
GU448996	D	D13	Miao, Y.-W., Peng, M.-S., Wu, G.-S., Ouyang, Y.-N., Yang, Z.-Y., Yu, N., Liang, J.-P., Pianchou, G., Beja-Pereira, A., Mitra, B., Palanichamy, M. G., Baig, M., Chaudhuri, T. K., Shen, Y.-Y., Kong, Q.-P., Murphy, R. W., Yao, Y.-G., & Zhang, Y.-P. (2013). Chicken domestication: an updated perspective based on mitochondrial genomes. Heredity, 110(3), 277–282. https://doi.org/10.1038/hdy.2012.83
D82919	D	D14	Fumihito, A., Miyake, T., Takada, M., Shingu, R., Endo, T., Gojobori, T., Kondo, N., & Ohno, S. (1996). Monophyletic origin and unique dispersal patterns of domestic fowls. *Proceedings of the National Academy of Sciences of the United States of America* , *93* (13), 6792–6795. https://doi.org/10.1073/pnas.93.13.6792
AF512152	D	D15	Liu, Y. P., Wu, G. S., Yao, Y. G., Miao, Y. W., Luikart, G., Baig, M., Beja-Pereira, A., Ding, Z. L., Palanichamy, M. G., & Zhang, Y. P. (2006). Multiple maternal origins of chickens: out of the Asian jungles. *Molecular phylogenetics and evolution* , *38* (1), 12–19. https://doi.org/10.1016/j.ympev.2005.09.014
GU448400	D	D16	Miao, Y.-W., Peng, M.-S., Wu, G.-S., Ouyang, Y.-N., Yang, Z.-Y., Yu, N., Liang, J.-P., Pianchou, G., Beja-Pereira, A., Mitra, B., Palanichamy, M. G., Baig, M., Chaudhuri, T. K., Shen, Y.-Y., Kong, Q.-P., Murphy, R. W., Yao, Y.-G., & Zhang, Y.-P. (2013). Chicken domestication: an updated perspective based on mitochondrial genomes. Heredity, 110(3), 277–282. https://doi.org/10.1038/hdy.2012.83
GU448475	D	D17	Miao, Y.-W., Peng, M.-S., Wu, G.-S., Ouyang, Y.-N., Yang, Z.-Y., Yu, N., Liang, J.-P., Pianchou, G., Beja-Pereira, A., Mitra, B., Palanichamy, M. G., Baig, M., Chaudhuri, T. K., Shen, Y.-Y., Kong, Q.-P., Murphy, R. W., Yao, Y.-G., & Zhang, Y.-P. (2013). Chicken domestication: an updated perspective based on mitochondrial genomes. Heredity, 110(3), 277–282. https://doi.org/10.1038/hdy.2012.83
AM746044	D	D18	Muchadeyi, F. C., Eding, H., Simianer, H., Wollny, C. B., Groeneveld, E., & Weigend, S. (2008). Mitochondrial DNA D-loop sequences suggest a Southeast Asian and Indian origin of Zimbabwean village chickens. *Animal genetics* , *39* (6), 615–622. https://doi.org/10.1111/j.1365-2052.2008.01785.x
EU199942	D	D19	Silva, P., Guan, X., Ho-Shing, O., Jones, J., Xu, J., Hui, D., Notter, D., & Smith, E. (2009). Mitochondrial DNA-based analysis of genetic variation and relatedness among Sri Lankan indigenous chickens and the Ceylon junglefowl (Gallus lafayetti). *Animal genetics* , *40* (1), 1–9. https://doi.org/10.1111/j.1365-2052.2008.01783.x
EF535242	D	D20	Storey, A. A., Ramírez, J. M., Quiroz, D., Burley, D. V., Addison, D. J., Walter, R., Anderson, A. J., Hunt, T. L., Athens, J. S., Huynen, L., & Matisoo-Smith, E. A. (2007). Radiocarbon and DNA evidence for a pre-Columbian introduction of Polynesian chickens to Chile. *Proceedings of the National Academy of Sciences of the United States of America* , *104* (25), 10335–10339. https://doi.org/10.1073/pnas.0703993104
AB268526	D	D21	Oka, T., Ino, Y., Nomura, K., Kawashima, S., Kuwayama, T., Hanada, H., Amano, T., Takada, M., Takahata, N., Hayashi, Y., & Akishinonomiya, F. (2007). Analysis of mtDNA sequences shows Japanese native chickens have multiple origins. *Animal genetics* , *38* (3), 287–293. https://doi.org/10.1111/j.1365-2052.2007.01604.x
AB268524	D	D22	Oka, T., Ino, Y., Nomura, K., Kawashima, S., Kuwayama, T., Hanada, H., Amano, T., Takada, M., Takahata, N., Hayashi, Y., & Akishinonomiya, F. (2007). Analysis of mtDNA sequences shows Japanese native chickens have multiple origins. *Animal genetics* , *38* (3), 287–293. https://doi.org/10.1111/j.1365-2052.2007.01604.x
AY704704	D	D25	Liu, Y. P., Wu, G. S., Yao, Y. G., Miao, Y. W., Luikart, G., Baig, M., Beja-Pereira, A., Ding, Z. L., Palanichamy, M. G., & Zhang, Y. P. (2006). Multiple maternal origins of chickens: out of the Asian jungles. *Molecular phylogenetics and evolution* , *38* (1), 12–19. https://doi.org/10.1016/j.ympev.2005.09.014
AY588637	D	D26	Qu et al. 2004 Direct Submission
AM746027	D	D27	Muchadeyi, F. C., Eding, H., Simianer, H., Wollny, C. B., Groeneveld, E., & Weigend, S. (2008). Mitochondrial DNA D-loop sequences suggest a Southeast Asian and Indian origin of Zimbabwean village chickens. *Animal genetics* , *39* (6), 615–622. https://doi.org/10.1111/j.1365-2052.2008.01785.x
AB268527	D	D28	Oka, T., Ino, Y., Nomura, K., Kawashima, S., Kuwayama, T., Hanada, H., Amano, T., Takada, M., Takahata, N., Hayashi, Y., & Akishinonomiya, F. (2007). Analysis of mtDNA sequences shows Japanese native chickens have multiple origins. *Animal genetics* , *38* (3), 287–293. https://doi.org/10.1111/j.1365-2052.2007.01604.x
AM746030	D	D29	Muchadeyi, F. C., Eding, H., Simianer, H., Wollny, C. B., Groeneveld, E., & Weigend, S. (2008). Mitochondrial DNA D-loop sequences suggest a Southeast Asian and Indian origin of Zimbabwean village chickens. *Animal genetics* , *39* (6), 615–622. https://doi.org/10.1111/j.1365-2052.2008.01785.x
GU448726	D	D30	Miao, Y.-W., Peng, M.-S., Wu, G.-S., Ouyang, Y.-N., Yang, Z.-Y., Yu, N., Liang, J.-P., Pianchou, G., Beja-Pereira, A., Mitra, B., Palanichamy, M. G., Baig, M., Chaudhuri, T. K., Shen, Y.-Y., Kong, Q.-P., Murphy, R. W., Yao, Y.-G., & Zhang, Y.-P. (2013). Chicken domestication: an updated perspective based on mitochondrial genomes. Heredity, 110(3), 277–282. https://doi.org/10.1038/hdy.2012.83
GU447616	D	D31	Miao, Y.-W., Peng, M.-S., Wu, G.-S., Ouyang, Y.-N., Yang, Z.-Y., Yu, N., Liang, J.-P., Pianchou, G., Beja-Pereira, A., Mitra, B., Palanichamy, M. G., Baig, M., Chaudhuri, T. K., Shen, Y.-Y., Kong, Q.-P., Murphy, R. W., Yao, Y.-G., & Zhang, Y.-P. (2013). Chicken domestication: an updated perspective based on mitochondrial genomes. Heredity, 110(3), 277–282. https://doi.org/10.1038/hdy.2012.83
FJ914364	D	D32	Dancause, K. N., Vilar, M. G., Steffy, R., & Lum, J. K. (2011). Characterizing genetic diversity of contemporary pacific chickens using mitochondrial DNA analyses. *PloS one* , *6* (2), e16843. https://doi.org/10.1371/journal.pone.0016843
FJ914347	D	D33	Dancause, K. N., Vilar, M. G., Steffy, R., & Lum, J. K. (2011). Characterizing genetic diversity of contemporary pacific chickens using mitochondrial DNA analyses. *PloS one* , *6* (2), e16843. https://doi.org/10.1371/journal.pone.0016843
FJ914346	D	D34	Dancause, K. N., Vilar, M. G., Steffy, R., & Lum, J. K. (2011). Characterizing genetic diversity of contemporary pacific chickens using mitochondrial DNA analyses. *PloS one* , *6* (2), e16843. https://doi.org/10.1371/journal.pone.0016843
FJ914363	D	D35	Dancause, K. N., Vilar, M. G., Steffy, R., & Lum, J. K. (2011). Characterizing genetic diversity of contemporary pacific chickens using mitochondrial DNA analyses. *PloS one* , *6* (2), e16843. https://doi.org/10.1371/journal.pone.0016843

FJ914323	D	D36	Dancause, K. N., Vilar, M. G., Steffy, R., & Lum, J. K. (2011). Characterizing genetic diversity of contemporary pacific chickens using mitochondrial DNA analyses. *PloS one* , *6* (2), e16843. https://doi.org/10.1371/journal.pone.0016843
FJ914362	D	D37	Dancause, K. N., Vilar, M. G., Steffy, R., & Lum, J. K. (2011). Characterizing genetic diversity of contemporary pacific chickens using mitochondrial DNA analyses. *PloS one* , *6* (2), e16843. https://doi.org/10.1371/journal.pone.0016843
FJ914333	D	D38	Dancause, K. N., Vilar, M. G., Steffy, R., & Lum, J. K. (2011). Characterizing genetic diversity of contemporary pacific chickens using mitochondrial DNA analyses. *PloS one* , *6* (2), e16843. https://doi.org/10.1371/journal.pone.0016843
EU329410	D	D39	Eriksson, J., Larson, G., Gunnarsson, U., Bed'hom, B., Tixier-Boichard, M., Strömstedt, L., Wright, D., Jungerius, A., Vereijken, A., Randi, E., Jensen, P., & Andersson, L. (2008). Identification of the yellow skin gene reveals a hybrid origin of the domestic chicken. *PLoS genetics* , *4* (2), e1000010. https://doi.org/10.1371/journal.pgen.1000010
AB368437	D	D40	Sakahira 2007 Direct Submission
EF535247	D	D41	Storey, A. A., Ramírez, J. M., Quiroz, D., Burley, D. V., Addison, D. J., Walter, R., Anderson, A. J., Hunt, T. L., Athens, J. S., Huynen, L., & Matisoo-Smith, E. A. (2007). Radiocarbon and DNA evidence for a pre-Columbian introduction of Polynesian chickens to Chile. *Proceedings of the National Academy of Sciences of the United States of America* , *104* (25), 10335–10339. https://doi.org/10.1073/pnas.0703993104
EF535244	D	D42	Storey, A. A., Ramírez, J. M., Quiroz, D., Burley, D. V., Addison, D. J., Walter, R., Anderson, A. J., Hunt, T. L., Athens, J. S., Huynen, L., & Matisoo-Smith, E. A. (2007). Radiocarbon and DNA evidence for a pre-Columbian introduction of Polynesian chickens to Chile. *Proceedings of the National Academy of Sciences of the United States of America* , *104* (25), 10335–10339. https://doi.org/10.1073/pnas.0703993104
GU557143	D	D43	Arora et al. 2010 Direct Submission
GU557145	D	D44	Arora et al. 2010 Direct Submission
EU199943	D	D45	Silva, P., Guan, X., Ho-Shing, O., Jones, J., Xu, J., Hui, D., Notter, D., & Smith, E. (2009). Mitochondrial DNA-based analysis of genetic variation and relatedness among Sri Lankan indigenous chickens and the Ceylon junglefowl (Gallus lafayetti). *Animal genetics* , *40* (1), 1–9. https://doi.org/10.1111/j.1365-2052.2008.01783.x
EU095034	D	D46	Mwacharo et al. 2008 Direct Submission
AM746026	D	D47	Muchadeyi, F. C., Eding, H., Simianer, H., Wollny, C. B., Groeneveld, E., & Weigend, S. (2008). Mitochondrial DNA D-loop sequences suggest a Southeast Asian and Indian origin of Zimbabwean village chickens. *Animal genetics* , *39* (6), 615–622. https://doi.org/10.1111/j.1365-2052.2008.01785.x
EU095054	D	D48	Mwacharo et al. 2008 Direct Submission
EU095045	D	D49	Mwacharo et al. 2008 Direct Submission
EU095037	D	D50	Mwacharo et al. 2008 Direct Submission
AM746025	D	D51	Muchadeyi, F. C., Eding, H., Simianer, H., Wollny, C. B., Groeneveld, E., & Weigend, S. (2008). Mitochondrial DNA D-loop sequences suggest a Southeast Asian and Indian origin of Zimbabwean village chickens. *Animal genetics* , *39* (6), 615–622. https://doi.org/10.1111/j.1365-2052.2008.01785.x
EU095082	D	D52	Mwacharo et al. 2008 Direct Submission
EU095051	D	D53	Mwacharo et al. 2008 Direct Submission
GU449070	E1	E01	Miao, Y.-W., Peng, M.-S., Wu, G.-S., Ouyang, Y.-N., Yang, Z.-Y., Yu, N., Liang, J.-P., Pianchou, G., Beja-Pereira, A., Mitra, B., Palanichamy, M. G., Baig, M., Chaudhuri, T. K., Shen, Y.-Y., Kong, Q.-P., Murphy, R. W., Yao, Y.-G., & Zhang, Y.-P. (2013). Chicken domestication: an updated perspective based on mitochondrial genomes. Heredity, 110(3), 277–282. https://doi.org/10.1038/hdy.2012.83
GU447958	E1	E02	Miao, Y.-W., Peng, M.-S., Wu, G.-S., Ouyang, Y.-N., Yang, Z.-Y., Yu, N., Liang, J.-P., Pianchou, G., Beja-Pereira, A., Mitra, B., Palanichamy, M. G., Baig, M., Chaudhuri, T. K., Shen, Y.-Y., Kong, Q.-P., Murphy, R. W., Yao, Y.-G., & Zhang, Y.-P. (2013). Chicken domestication: an updated perspective based on mitochondrial genomes. Heredity, 110(3), 277–282. https://doi.org/10.1038/hdy.2012.83
GU449088	E1	E03	Miao, Y.-W., Peng, M.-S., Wu, G.-S., Ouyang, Y.-N., Yang, Z.-Y., Yu, N., Liang, J.-P., Pianchou, G., Beja-Pereira, A., Mitra, B., Palanichamy, M. G., Baig, M., Chaudhuri, T. K., Shen, Y.-Y., Kong, Q.-P., Murphy, R. W., Yao, Y.-G., & Zhang, Y.-P. (2013). Chicken domestication: an updated perspective based on mitochondrial genomes. Heredity, 110(3), 277–282. https://doi.org/10.1038/hdy.2012.83
GU448982	E1	E04	Miao, Y.-W., Peng, M.-S., Wu, G.-S., Ouyang, Y.-N., Yang, Z.-Y., Yu, N., Liang, J.-P., Pianchou, G., Beja-Pereira, A., Mitra, B., Palanichamy, M. G., Baig, M., Chaudhuri, T. K., Shen, Y.-Y., Kong, Q.-P., Murphy, R. W., Yao, Y.-G., & Zhang, Y.-P. (2013). Chicken domestication: an updated perspective based on mitochondrial genomes. Heredity, 110(3), 277–282. https://doi.org/10.1038/hdy.2012.83
AF512230	E1	E05	Liu, Y. P., Wu, G. S., Yao, Y. G., Miao, Y. W., Luikart, G., Baig, M., Beja-Pereira, A., Ding, Z. L., Palanichamy, M. G., & Zhang, Y. P. (2006). Multiple maternal origins of chickens: out of the Asian jungles. *Molecular phylogenetics and evolution* , *38* (1), 12–19. https://doi.org/10.1016/j.ympev.2005.09.014
GU447818	E1	E06	Miao, Y.-W., Peng, M.-S., Wu, G.-S., Ouyang, Y.-N., Yang, Z.-Y., Yu, N., Liang, J.-P., Pianchou, G., Beja-Pereira, A., Mitra, B., Palanichamy, M. G., Baig, M., Chaudhuri, T. K., Shen, Y.-Y., Kong, Q.-P., Murphy, R. W., Yao, Y.-G., & Zhang, Y.-P. (2013). Chicken domestication: an updated perspective based on mitochondrial genomes. Heredity, 110(3), 277–282. https://doi.org/10.1038/hdy.2012.83
GU447973	E1	E07	Miao, Y.-W., Peng, M.-S., Wu, G.-S., Ouyang, Y.-N., Yang, Z.-Y., Yu, N., Liang, J.-P., Pianchou, G., Beja-Pereira, A., Mitra, B., Palanichamy, M. G., Baig, M., Chaudhuri, T. K., Shen, Y.-Y., Kong, Q.-P., Murphy, R. W., Yao, Y.-G., & Zhang, Y.-P. (2013). Chicken domestication: an updated perspective based on mitochondrial genomes. Heredity, 110(3), 277–282. https://doi.org/10.1038/hdy.2012.83
GU447952	E1	E08	Miao, Y.-W., Peng, M.-S., Wu, G.-S., Ouyang, Y.-N., Yang, Z.-Y., Yu, N., Liang, J.-P., Pianchou, G., Beja-Pereira, A., Mitra, B., Palanichamy, M. G., Baig, M., Chaudhuri, T. K., Shen, Y.-Y., Kong, Q.-P., Murphy, R. W., Yao, Y.-G., & Zhang, Y.-P. (2013). Chicken domestication: an updated perspective based on mitochondrial genomes. Heredity, 110(3), 277–282. https://doi.org/10.1038/hdy.2012.83
GU449095	E1	E09	Miao, Y.-W., Peng, M.-S., Wu, G.-S., Ouyang, Y.-N., Yang, Z.-Y., Yu, N., Liang, J.-P., Pianchou, G., Beja-Pereira, A., Mitra, B., Palanichamy, M. G., Baig, M., Chaudhuri, T. K., Shen, Y.-Y., Kong, Q.-P., Murphy, R. W., Yao, Y.-G., & Zhang, Y.-P. (2013). Chicken domestication: an updated perspective based on mitochondrial genomes. Heredity, 110(3), 277–282. https://doi.org/10.1038/hdy.2012.83
GU447581	E1	E10	Miao, Y.-W., Peng, M.-S., Wu, G.-S., Ouyang, Y.-N., Yang, Z.-Y., Yu, N., Liang, J.-P., Pianchou, G., Beja-Pereira, A., Mitra, B., Palanichamy, M. G., Baig, M., Chaudhuri, T. K., Shen, Y.-Y., Kong, Q.-P., Murphy, R. W., Yao, Y.-G., & Zhang, Y.-P. (2013). Chicken domestication: an updated perspective based on mitochondrial genomes. Heredity, 110(3), 277–282. https://doi.org/10.1038/hdy.2012.83
GU449066	E1	E11	Miao, Y.-W., Peng, M.-S., Wu, G.-S., Ouyang, Y.-N., Yang, Z.-Y., Yu, N., Liang, J.-P., Pianchou, G., Beja-Pereira, A., Mitra, B., Palanichamy, M. G., Baig, M., Chaudhuri, T. K., Shen, Y.-Y., Kong, Q.-P., Murphy, R. W., Yao, Y.-G., & Zhang, Y.-P. (2013). Chicken domestication: an updated perspective based on mitochondrial genomes. Heredity, 110(3), 277–282. https://doi.org/10.1038/hdy.2012.83
EU095125	E1	E12	Mwacharo et al. 2008 Direct Submission
GU447586	E3	E13	Miao, Y.-W., Peng, M.-S., Wu, G.-S., Ouyang, Y.-N., Yang, Z.-Y., Yu, N., Liang, J.-P., Pianchou, G., Beja-Pereira, A., Mitra, B., Palanichamy, M. G., Baig, M., Chaudhuri, T. K., Shen, Y.-Y., Kong, Q.-P., Murphy, R. W., Yao, Y.-G., & Zhang, Y.-P. (2013). Chicken domestication: an updated perspective based on mitochondrial genomes. Heredity, 110(3), 277–282. https://doi.org/10.1038/hdy.2012.83
AM746045	E1	E14	Muchadeyi, F. C., Eding, H., Simianer, H., Wollny, C. B., Groeneveld, E., & Weigend, S. (2008). Mitochondrial DNA D-loop sequences suggest a Southeast Asian and Indian origin of Zimbabwean village chickens. *Animal genetics* , *39* (6), 615–622. https://doi.org/10.1111/j.1365-2052.2008.01785.x
GU448514	E1	E15	Miao, Y.-W., Peng, M.-S., Wu, G.-S., Ouyang, Y.-N., Yang, Z.-Y., Yu, N., Liang, J.-P., Pianchou, G., Beja-Pereira, A., Mitra, B., Palanichamy, M. G., Baig, M., Chaudhuri, T. K., Shen, Y.-Y., Kong, Q.-P., Murphy, R. W., Yao, Y.-G., & Zhang, Y.-P. (2013). Chicken domestication: an updated perspective based on mitochondrial genomes. Heredity, 110(3), 277–282. https://doi.org/10.1038/hdy.2012.83
AY704713	E1	E16	Liu, Y. P., Wu, G. S., Yao, Y. G., Miao, Y. W., Luikart, G., Baig, M., Beja-Pereira, A., Ding, Z. L., Palanichamy, M. G., & Zhang, Y. P. (2006). Multiple maternal origins of chickens: out of the Asian jungles. *Molecular phylogenetics and evolution* , *38* (1), 12–19. https://doi.org/10.1016/j.ympev.2005.09.014
GU448358	E2	E17	Miao, Y.-W., Peng, M.-S., Wu, G.-S., Ouyang, Y.-N., Yang, Z.-Y., Yu, N., Liang, J.-P., Pianchou, G., Beja-Pereira, A., Mitra, B., Palanichamy, M. G., Baig, M., Chaudhuri, T. K., Shen, Y.-Y., Kong, Q.-P., Murphy, R. W., Yao, Y.-G., & Zhang, Y.-P. (2013). Chicken domestication: an updated perspective based on mitochondrial genomes. Heredity, 110(3), 277–282. https://doi.org/10.1038/hdy.2012.83
GU448362	E3	E18	Miao, Y.-W., Peng, M.-S., Wu, G.-S., Ouyang, Y.-N., Yang, Z.-Y., Yu, N., Liang, J.-P., Pianchou, G., Beja-Pereira, A., Mitra, B., Palanichamy, M. G., Baig, M., Chaudhuri, T. K., Shen, Y.-Y., Kong, Q.-P., Murphy, R. W., Yao, Y.-G., & Zhang, Y.-P. (2013). Chicken domestication: an updated perspective based on mitochondrial genomes. Heredity, 110(3), 277–282. https://doi.org/10.1038/hdy.2012.83
GU447791	E1	E19	Miao, Y.-W., Peng, M.-S., Wu, G.-S., Ouyang, Y.-N., Yang, Z.-Y., Yu, N., Liang, J.-P., Pianchou, G., Beja-Pereira, A., Mitra, B., Palanichamy, M. G., Baig, M., Chaudhuri, T. K., Shen, Y.-Y., Kong, Q.-P., Murphy, R. W., Yao, Y.-G., & Zhang, Y.-P. (2013). Chicken domestication: an updated perspective based on mitochondrial genomes. Heredity, 110(3), 277–282. https://doi.org/10.1038/hdy.2012.83
EU847809	E3	E20	Kanginakudru, S., Metta, M., Jakati, R. D., & Nagaraju, J. (2008). Genetic evidence from Indian red jungle fowl corroborates multiple domestication of modern day chicken. *BMC evolutionary biology* , *8* , 174. https://doi.org/10.1186/1471-2148-8-174
GU448948	E1	E21	Miao, Y.-W., Peng, M.-S., Wu, G.-S., Ouyang, Y.-N., Yang, Z.-Y., Yu, N., Liang, J.-P., Pianchou, G., Beja-Pereira, A., Mitra, B., Palanichamy, M. G., Baig, M., Chaudhuri, T. K., Shen, Y.-Y., Kong, Q.-P., Murphy, R. W., Yao, Y.-G., & Zhang, Y.-P. (2013). Chicken domestication: an updated perspective based on mitochondrial genomes. Heredity, 110(3), 277–282. https://doi.org/10.1038/hdy.2012.83
GU448458	E2	E22	Miao, Y.-W., Peng, M.-S., Wu, G.-S., Ouyang, Y.-N., Yang, Z.-Y., Yu, N., Liang, J.-P., Pianchou, G., Beja-Pereira, A., Mitra, B., Palanichamy, M. G., Baig, M., Chaudhuri, T. K., Shen, Y.-Y., Kong, Q.-P., Murphy, R. W., Yao, Y.-G., & Zhang, Y.-P. (2013). Chicken domestication: an updated perspective based on mitochondrial genomes. Heredity, 110(3), 277–282. https://doi.org/10.1038/hdy.2012.83

GU448450	E2	E24	Miao, Y.-W., Peng, M.-S., Wu, G.-S., Ouyang, Y.-N., Yang, Z.-Y., Yu, N., Liang, J.-P., Pianchou, G., Beja-Pereira, A., Mitra, B., Palanichamy, M. G., Baig, M., Chaudhuri, T. K., Shen, Y.-Y., Kong, Q.-P., Murphy, R. W., Yao, Y.-G., & Zhang, Y.-P. (2013). Chicken domestication: an updated perspective based on mitochondrial genomes. Heredity, 110(3), 277–282. https://doi.org/10.1038/hdy.2012.83
GU448469	E2	E25	Miao, Y.-W., Peng, M.-S., Wu, G.-S., Ouyang, Y.-N., Yang, Z.-Y., Yu, N., Liang, J.-P., Pianchou, G., Beja-Pereira, A., Mitra, B., Palanichamy, M. G., Baig, M., Chaudhuri, T. K., Shen, Y.-Y., Kong, Q.-P., Murphy, R. W., Yao, Y.-G., & Zhang, Y.-P. (2013). Chicken domestication: an updated perspective based on mitochondrial genomes. Heredity, 110(3), 277–282. https://doi.org/10.1038/hdy.2012.83
GU448950	E3	E26	Miao, Y.-W., Peng, M.-S., Wu, G.-S., Ouyang, Y.-N., Yang, Z.-Y., Yu, N., Liang, J.-P., Pianchou, G., Beja-Pereira, A., Mitra, B., Palanichamy, M. G., Baig, M., Chaudhuri, T. K., Shen, Y.-Y., Kong, Q.-P., Murphy, R. W., Yao, Y.-G., & Zhang, Y.-P. (2013). Chicken domestication: an updated perspective based on mitochondrial genomes. Heredity, 110(3), 277–282. https://doi.org/10.1038/hdy.2012.83
GU448964	E3	E27	Miao, Y.-W., Peng, M.-S., Wu, G.-S., Ouyang, Y.-N., Yang, Z.-Y., Yu, N., Liang, J.-P., Pianchou, G., Beja-Pereira, A., Mitra, B., Palanichamy, M. G., Baig, M., Chaudhuri, T. K., Shen, Y.-Y., Kong, Q.-P., Murphy, R. W., Yao, Y.-G., & Zhang, Y.-P. (2013). Chicken domestication: an updated perspective based on mitochondrial genomes. Heredity, 110(3), 277–282. https://doi.org/10.1038/hdy.2012.83
GU448953	E3	E28	Miao, Y.-W., Peng, M.-S., Wu, G.-S., Ouyang, Y.-N., Yang, Z.-Y., Yu, N., Liang, J.-P., Pianchou, G., Beja-Pereira, A., Mitra, B., Palanichamy, M. G., Baig, M., Chaudhuri, T. K., Shen, Y.-Y., Kong, Q.-P., Murphy, R. W., Yao, Y.-G., & Zhang, Y.-P. (2013). Chicken domestication: an updated perspective based on mitochondrial genomes. Heredity, 110(3), 277–282. https://doi.org/10.1038/hdy.2012.83
GU448758	E3	E29	Miao, Y.-W., Peng, M.-S., Wu, G.-S., Ouyang, Y.-N., Yang, Z.-Y., Yu, N., Liang, J.-P., Pianchou, G., Beja-Pereira, A., Mitra, B., Palanichamy, M. G., Baig, M., Chaudhuri, T. K., Shen, Y.-Y., Kong, Q.-P., Murphy, R. W., Yao, Y.-G., & Zhang, Y.-P. (2013). Chicken domestication: an updated perspective based on mitochondrial genomes. Heredity, 110(3), 277–282. https://doi.org/10.1038/hdy.2012.83
GU447982	E3	E30	Miao, Y.-W., Peng, M.-S., Wu, G.-S., Ouyang, Y.-N., Yang, Z.-Y., Yu, N., Liang, J.-P., Pianchou, G., Beja-Pereira, A., Mitra, B., Palanichamy, M. G., Baig, M., Chaudhuri, T. K., Shen, Y.-Y., Kong, Q.-P., Murphy, R. W., Yao, Y.-G., & Zhang, Y.-P. (2013). Chicken domestication: an updated perspective based on mitochondrial genomes. Heredity, 110(3), 277–282. https://doi.org/10.1038/hdy.2012.83
GU448363	E2	E31	Miao, Y.-W., Peng, M.-S., Wu, G.-S., Ouyang, Y.-N., Yang, Z.-Y., Yu, N., Liang, J.-P., Pianchou, G., Beja-Pereira, A., Mitra, B., Palanichamy, M. G., Baig, M., Chaudhuri, T. K., Shen, Y.-Y., Kong, Q.-P., Murphy, R. W., Yao, Y.-G., & Zhang, Y.-P. (2013). Chicken domestication: an updated perspective based on mitochondrial genomes. Heredity, 110(3), 277–282. https://doi.org/10.1038/hdy.2012.83
GU448973	E1	E32	Miao, Y.-W., Peng, M.-S., Wu, G.-S., Ouyang, Y.-N., Yang, Z.-Y., Yu, N., Liang, J.-P., Pianchou, G., Beja-Pereira, A., Mitra, B., Palanichamy, M. G., Baig, M., Chaudhuri, T. K., Shen, Y.-Y., Kong, Q.-P., Murphy, R. W., Yao, Y.-G., & Zhang, Y.-P. (2013). Chicken domestication: an updated perspective based on mitochondrial genomes. Heredity, 110(3), 277–282. https://doi.org/10.1038/hdy.2012.83
GU448648	E1	E33	Miao, Y.-W., Peng, M.-S., Wu, G.-S., Ouyang, Y.-N., Yang, Z.-Y., Yu, N., Liang, J.-P., Pianchou, G., Beja-Pereira, A., Mitra, B., Palanichamy, M. G., Baig, M., Chaudhuri, T. K., Shen, Y.-Y., Kong, Q.-P., Murphy, R. W., Yao, Y.-G., & Zhang, Y.-P. (2013). Chicken domestication: an updated perspective based on mitochondrial genomes. Heredity, 110(3), 277–282. https://doi.org/10.1038/hdy.2012.83
GU448468	E1	E34	Miao, Y.-W., Peng, M.-S., Wu, G.-S., Ouyang, Y.-N., Yang, Z.-Y., Yu, N., Liang, J.-P., Pianchou, G., Beja-Pereira, A., Mitra, B., Palanichamy, M. G., Baig, M., Chaudhuri, T. K., Shen, Y.-Y., Kong, Q.-P., Murphy, R. W., Yao, Y.-G., & Zhang, Y.-P. (2013). Chicken domestication: an updated perspective based on mitochondrial genomes. Heredity, 110(3), 277–282. https://doi.org/10.1038/hdy.2012.83
GU448601	E1	E35	Miao, Y.-W., Peng, M.-S., Wu, G.-S., Ouyang, Y.-N., Yang, Z.-Y., Yu, N., Liang, J.-P., Pianchou, G., Beja-Pereira, A., Mitra, B., Palanichamy, M. G., Baig, M., Chaudhuri, T. K., Shen, Y.-Y., Kong, Q.-P., Murphy, R. W., Yao, Y.-G., & Zhang, Y.-P. (2013). Chicken domestication: an updated perspective based on mitochondrial genomes. Heredity, 110(3), 277–282. https://doi.org/10.1038/hdy.2012.83
GU448453	E1	E36	Miao, Y.-W., Peng, M.-S., Wu, G.-S., Ouyang, Y.-N., Yang, Z.-Y., Yu, N., Liang, J.-P., Pianchou, G., Beja-Pereira, A., Mitra, B., Palanichamy, M. G., Baig, M., Chaudhuri, T. K., Shen, Y.-Y., Kong, Q.-P., Murphy, R. W., Yao, Y.-G., & Zhang, Y.-P. (2013). Chicken domestication: an updated perspective based on mitochondrial genomes. Heredity, 110(3), 277–282. https://doi.org/10.1038/hdy.2012.83
GU448952	E2	E37	Miao, Y.-W., Peng, M.-S., Wu, G.-S., Ouyang, Y.-N., Yang, Z.-Y., Yu, N., Liang, J.-P., Pianchou, G., Beja-Pereira, A., Mitra, B., Palanichamy, M. G., Baig, M., Chaudhuri, T. K., Shen, Y.-Y., Kong, Q.-P., Murphy, R. W., Yao, Y.-G., & Zhang, Y.-P. (2013). Chicken domestication: an updated perspective based on mitochondrial genomes. Heredity, 110(3), 277–282. https://doi.org/10.1038/hdy.2012.83
EF535248	E1	E38	Storey, A. A., Ramírez, J. M., Quiroz, D., Burley, D. V., Addison, D. J., Walter, R., Anderson, A. J., Hunt, T. L., Athens, J. S., Huynen, L., & Matisoo-Smith, E. A. (2007). Radiocarbon and DNA evidence for a pre-Columbian introduction of Polynesian chickens to Chile. *Proceedings of the National Academy of Sciences of the United States of America* , *104* (25), 10335–10339. https://doi.org/10.1073/pnas.0703993104
GU448414	E1	E39	Miao, Y.-W., Peng, M.-S., Wu, G.-S., Ouyang, Y.-N., Yang, Z.-Y., Yu, N., Liang, J.-P., Pianchou, G., Beja-Pereira, A., Mitra, B., Palanichamy, M. G., Baig, M., Chaudhuri, T. K., Shen, Y.-Y., Kong, Q.-P., Murphy, R. W., Yao, Y.-G., & Zhang, Y.-P. (2013). Chicken domestication: an updated perspective based on mitochondrial genomes. Heredity, 110(3), 277–282. https://doi.org/10.1038/hdy.2012.83
GU448364	E1	E40	Miao, Y.-W., Peng, M.-S., Wu, G.-S., Ouyang, Y.-N., Yang, Z.-Y., Yu, N., Liang, J.-P., Pianchou, G., Beja-Pereira, A., Mitra, B., Palanichamy, M. G., Baig, M., Chaudhuri, T. K., Shen, Y.-Y., Kong, Q.-P., Murphy, R. W., Yao, Y.-G., & Zhang, Y.-P. (2013). Chicken domestication: an updated perspective based on mitochondrial genomes. Heredity, 110(3), 277–282. https://doi.org/10.1038/hdy.2012.83
GU448409	E1	E41	Miao, Y.-W., Peng, M.-S., Wu, G.-S., Ouyang, Y.-N., Yang, Z.-Y., Yu, N., Liang, J.-P., Pianchou, G., Beja-Pereira, A., Mitra, B., Palanichamy, M. G., Baig, M., Chaudhuri, T. K., Shen, Y.-Y., Kong, Q.-P., Murphy, R. W., Yao, Y.-G., & Zhang, Y.-P. (2013). Chicken domestication: an updated perspective based on mitochondrial genomes. Heredity, 110(3), 277–282. https://doi.org/10.1038/hdy.2012.83
GU448766	E2	E42	Miao, Y.-W., Peng, M.-S., Wu, G.-S., Ouyang, Y.-N., Yang, Z.-Y., Yu, N., Liang, J.-P., Pianchou, G., Beja-Pereira, A., Mitra, B., Palanichamy, M. G., Baig, M., Chaudhuri, T. K., Shen, Y.-Y., Kong, Q.-P., Murphy, R. W., Yao, Y.-G., & Zhang, Y.-P. (2013). Chicken domestication: an updated perspective based on mitochondrial genomes. Heredity, 110(3), 277–282. https://doi.org/10.1038/hdy.2012.83
GU448378	E1	E43	Miao, Y.-W., Peng, M.-S., Wu, G.-S., Ouyang, Y.-N., Yang, Z.-Y., Yu, N., Liang, J.-P., Pianchou, G., Beja-Pereira, A., Mitra, B., Palanichamy, M. G., Baig, M., Chaudhuri, T. K., Shen, Y.-Y., Kong, Q.-P., Murphy, R. W., Yao, Y.-G., & Zhang, Y.-P. (2013). Chicken domestication: an updated perspective based on mitochondrial genomes. Heredity, 110(3), 277–282. https://doi.org/10.1038/hdy.2012.83
GU448389	E1	E44	Miao, Y.-W., Peng, M.-S., Wu, G.-S., Ouyang, Y.-N., Yang, Z.-Y., Yu, N., Liang, J.-P., Pianchou, G., Beja-Pereira, A., Mitra, B., Palanichamy, M. G., Baig, M., Chaudhuri, T. K., Shen, Y.-Y., Kong, Q.-P., Murphy, R. W., Yao, Y.-G., & Zhang, Y.-P. (2013). Chicken domestication: an updated perspective based on mitochondrial genomes. Heredity, 110(3), 277–282. https://doi.org/10.1038/hdy.2012.83
GU448411	E1	E45	Miao, Y.-W., Peng, M.-S., Wu, G.-S., Ouyang, Y.-N., Yang, Z.-Y., Yu, N., Liang, J.-P., Pianchou, G., Beja-Pereira, A., Mitra, B., Palanichamy, M. G., Baig, M., Chaudhuri, T. K., Shen, Y.-Y., Kong, Q.-P., Murphy, R. W., Yao, Y.-G., & Zhang, Y.-P. (2013). Chicken domestication: an updated perspective based on mitochondrial genomes. Heredity, 110(3), 277–282. https://doi.org/10.1038/hdy.2012.83
GU448413	E1	E46	Miao, Y.-W., Peng, M.-S., Wu, G.-S., Ouyang, Y.-N., Yang, Z.-Y., Yu, N., Liang, J.-P., Pianchou, G., Beja-Pereira, A., Mitra, B., Palanichamy, M. G., Baig, M., Chaudhuri, T. K., Shen, Y.-Y., Kong, Q.-P., Murphy, R. W., Yao, Y.-G., & Zhang, Y.-P. (2013). Chicken domestication: an updated perspective based on mitochondrial genomes. Heredity, 110(3), 277–282. https://doi.org/10.1038/hdy.2012.83
GU447988	E1	E47	Miao, Y.-W., Peng, M.-S., Wu, G.-S., Ouyang, Y.-N., Yang, Z.-Y., Yu, N., Liang, J.-P., Pianchou, G., Beja-Pereira, A., Mitra, B., Palanichamy, M. G., Baig, M., Chaudhuri, T. K., Shen, Y.-Y., Kong, Q.-P., Murphy, R. W., Yao, Y.-G., & Zhang, Y.-P. (2013). Chicken domestication: an updated perspective based on mitochondrial genomes. Heredity, 110(3), 277–282. https://doi.org/10.1038/hdy.2012.83
AM746046	E1	E48	Muchadeyi, F. C., Eding, H., Simianer, H., Wollny, C. B., Groeneveld, E., & Weigend, S. (2008). Mitochondrial DNA D-loop sequences suggest a Southeast Asian and Indian origin of Zimbabwean village chickens. *Animal genetics* , *39* (6), 615–622. https://doi.org/10.1111/j.1365-2052.2008.01785.x
GQ258691	E1	E49	Revay, T., Bodzsar, N., Mobegi, V. E., Hanotte, O., & Hidas, A. (2010). Origin of Hungarian indigenous chicken breeds inferred from mitochondrial DNA D-loop sequences. *Animal genetics* , *41* (5), 548–550. https://doi.org/10.1111/j.1365-2052.2010.02041.x
EU847811	E2	E50	Kanginakudru, S., Metta, M., Jakati, R. D., & Nagaraju, J. (2008). Genetic evidence from Indian red jungle fowl corroborates multiple domestication of modern day chicken. *BMC evolutionary biology* , *8* , 174. https://doi.org/10.1186/1471-2148-8-174
EF190837	E1	E52	Gongora, J., Rawlence, N. J., Mobegi, V. A., Jianlin, H., Alcalde, J. A., Matus, J. T., Hanotte, O., Moran, C., Austin, J. J., Ulm, S., Anderson, A. J., Larson, G., & Cooper, A. (2008). Indo-European and Asian origins for Chilean and Pacific chickens revealed by mtDNA. *Proceedings of the National Academy of Sciences of the United States of America* , *105* (30), 10308–10313. https://doi.org/10.1073/pnas.0801991105
EU199915	E2	E54	Silva, P., Guan, X., Ho-Shing, O., Jones, J., Xu, J., Hui, D., Notter, D., & Smith, E. (2009). Mitochondrial DNA-based analysis of genetic variation and relatedness among Sri Lankan indigenous chickens and the Ceylon junglefowl (Gallus lafayetti). *Animal genetics* , *40* (1), 1–9. https://doi.org/10.1111/j.1365-2052.2008.01783.x
EU199910	E1	E55	Silva, P., Guan, X., Ho-Shing, O., Jones, J., Xu, J., Hui, D., Notter, D., & Smith, E. (2009). Mitochondrial DNA-based analysis of genetic variation and relatedness among Sri Lankan indigenous chickens and the Ceylon junglefowl (Gallus lafayetti). *Animal genetics* , *40* (1), 1–9. https://doi.org/10.1111/j.1365-2052.2008.01783.x
EU199924	E1	E56	Silva, P., Guan, X., Ho-Shing, O., Jones, J., Xu, J., Hui, D., Notter, D., & Smith, E. (2009). Mitochondrial DNA-based analysis of genetic variation and relatedness among Sri Lankan indigenous chickens and the Ceylon junglefowl (Gallus lafayetti). *Animal genetics* , *40* (1), 1–9. https://doi.org/10.1111/j.1365-2052.2008.01783.x
EU199907	E1	E57	Silva, P., Guan, X., Ho-Shing, O., Jones, J., Xu, J., Hui, D., Notter, D., & Smith, E. (2009). Mitochondrial DNA-based analysis of genetic variation and relatedness among Sri Lankan indigenous chickens and the Ceylon junglefowl (Gallus lafayetti). *Animal genetics* , *40* (1), 1–9. https://doi.org/10.1111/j.1365-2052.2008.01783.x
EU199908	E1	E58	Silva, P., Guan, X., Ho-Shing, O., Jones, J., Xu, J., Hui, D., Notter, D., & Smith, E. (2009). Mitochondrial DNA-based analysis of genetic variation and relatedness among Sri Lankan indigenous chickens and the Ceylon junglefowl (Gallus lafayetti). *Animal genetics* , *40* (1), 1–9. https://doi.org/10.1111/j.1365-2052.2008.01783.x
EU199909	E1	E59	Silva, P., Guan, X., Ho-Shing, O., Jones, J., Xu, J., Hui, D., Notter, D., & Smith, E. (2009). Mitochondrial DNA-based analysis of genetic variation and relatedness among Sri Lankan indigenous chickens and the Ceylon junglefowl (Gallus lafayetti). *Animal genetics* , *40* (1), 1–9. https://doi.org/10.1111/j.1365-2052.2008.01783.x
EU199914	E1	E60	Silva, P., Guan, X., Ho-Shing, O., Jones, J., Xu, J., Hui, D., Notter, D., & Smith, E. (2009). Mitochondrial DNA-based analysis of genetic variation and relatedness among Sri Lankan indigenous chickens and the Ceylon junglefowl (Gallus lafayetti). *Animal genetics* , *40* (1), 1–9. https://doi.org/10.1111/j.1365-2052.2008.01783.x
EU199920	E3	E61	Silva, P., Guan, X., Ho-Shing, O., Jones, J., Xu, J., Hui, D., Notter, D., & Smith, E. (2009). Mitochondrial DNA-based analysis of genetic variation and relatedness among Sri Lankan indigenous chickens and the Ceylon junglefowl (Gallus lafayetti). *Animal genetics* , *40* (1), 1–9. https://doi.org/10.1111/j.1365-2052.2008.01783.x
EU199917	E2	E62	Silva, P., Guan, X., Ho-Shing, O., Jones, J., Xu, J., Hui, D., Notter, D., & Smith, E. (2009). Mitochondrial DNA-based analysis of genetic variation and relatedness among Sri Lankan indigenous chickens and the Ceylon junglefowl (Gallus lafayetti). *Animal genetics* , *40* (1), 1–9. https://doi.org/10.1111/j.1365-2052.2008.01783.x
EU199911	E1	E63	Silva, P., Guan, X., Ho-Shing, O., Jones, J., Xu, J., Hui, D., Notter, D., & Smith, E. (2009). Mitochondrial DNA-based analysis of genetic variation and relatedness among Sri Lankan indigenous chickens and the Ceylon junglefowl (Gallus lafayetti). *Animal genetics* , *40* (1), 1–9. https://doi.org/10.1111/j.1365-2052.2008.01783.x
EU199919	E3	E64	Silva, P., Guan, X., Ho-Shing, O., Jones, J., Xu, J., Hui, D., Notter, D., & Smith, E. (2009). Mitochondrial DNA-based analysis of genetic variation and relatedness among Sri Lankan indigenous chickens and the Ceylon junglefowl (Gallus lafayetti). *Animal genetics* , *40* (1), 1–9. https://doi.org/10.1111/j.1365-2052.2008.01783.x
EU199912	E1	E65	Silva, P., Guan, X., Ho-Shing, O., Jones, J., Xu, J., Hui, D., Notter, D., & Smith, E. (2009). Mitochondrial DNA-based analysis of genetic variation and relatedness among Sri Lankan indigenous chickens and the Ceylon junglefowl (Gallus lafayetti). *Animal genetics* , *40* (1), 1–9. https://doi.org/10.1111/j.1365-2052.2008.01783.x

EU095123	E1	E66	Mwacharo et al. 2008 Direct Submission
EU095057	E1	E67	Mwacharo et al. 2008 Direct Submission
EU095141	E1	E68	Mwacharo et al. 2008 Direct Submission
EU095122	E1	E69	Mwacharo et al. 2008 Direct Submission
EU095153	E1	E70	Mwacharo et al. 2008 Direct Submission
EU095059	E1	E71	Mwacharo et al. 2008 Direct Submission
EU095101	E1	E72	Mwacharo et al. 2008 Direct Submission
EU095036	E1	E73	Mwacharo et al. 2008 Direct Submission
EU095038	E1	E74	Mwacharo et al. 2008 Direct Submission
EU095159	E1	E75	Mwacharo et al. 2008 Direct Submission
EU095121	E1	E76	Mwacharo et al. 2008 Direct Submission
EU095163	E1	E77	Mwacharo et al. 2008 Direct Submission
EU095149	E1	E78	Mwacharo et al. 2008 Direct Submission
EU095169	E1	E79	Mwacharo et al. 2008 Direct Submission
AM886307	E1	E80	Muchadeyi, F. C., Eding, H., Simianer, H., Wollny, C. B., Groeneveld, E., & Weigend, S. (2008). Mitochondrial DNA D-loop sequences suggest a Southeast Asian and Indian origin of Zimbabwean village chickens. *Animal genetics* , *39* (6), 615–622. https://doi.org/10.1111/j.1365-2052.2008.01785.x
AM886306	E1	E81	Muchadeyi, F. C., Eding, H., Simianer, H., Wollny, C. B., Groeneveld, E., & Weigend, S. (2008). Mitochondrial DNA D-loop sequences suggest a Southeast Asian and Indian origin of Zimbabwean village chickens. *Animal genetics* , *39* (6), 615–622. https://doi.org/10.1111/j.1365-2052.2008.01785.x
GU447585	E2	E82	Miao, Y.-W., Peng, M.-S., Wu, G.-S., Ouyang, Y.-N., Yang, Z.-Y., Yu, N., Liang, J.-P., Pianchou, G., Beja-Pereira, A., Mitra, B., Palanichamy, M. G., Baig, M., Chaudhuri, T. K., Shen, Y.-Y., Kong, Q.-P., Murphy, R. W., Yao, Y.-G., & Zhang, Y.-P. (2013). Chicken domestication: an updated perspective based on mitochondrial genomes. Heredity, 110(3), 277–282. https://doi.org/10.1038/hdy.2012.83
AM746050	E1	E89	Muchadeyi, F. C., Eding, H., Simianer, H., Wollny, C. B., Groeneveld, E., & Weigend, S. (2008). Mitochondrial DNA D-loop sequences suggest a Southeast Asian and Indian origin of Zimbabwean village chickens. *Animal genetics* , *39* (6), 615–622. https://doi.org/10.1111/j.1365-2052.2008.01785.x
HQ189520	E1	E106	Arora et al. 2010 Direct Submission
GU557144	E1	E110	Arora et al. 2010 Direct Submission
HQ189522	E1	E111	Arora et al. 2010 Direct Submission
HQ189521	E1	E112	Arora et al. 2010 Direct Submission
GU557146	E3	E113	Arora et al. 2010 Direct Submission
HM189678	E1	E114	Storey, A. A., Spriggs, M., Bedford, S., Hawkins, S. C., Robins, J. H., Huynen, L., & Matisoo-Smith, E. (2010). Mitochondrial DNA from 3000-year old chickens at the Teouma site, Vanuatu. *Journal of Archaeological Science* , *37* (10), 2459–2468. https://doi.org/10.1016/j.jas.2010.05.006
AM746055	E1	E115	Muchadeyi, F. C., Eding, H., Simianer, H., Wollny, C. B., Groeneveld, E., & Weigend, S. (2008). Mitochondrial DNA D-loop sequences suggest a Southeast Asian and Indian origin of Zimbabwean village chickens. *Animal genetics* , *39* (6), 615–622. https://doi.org/10.1111/j.1365-2052.2008.01785.x
GU561992	E1	E116	Pratap et al. 2010 Direct Submission
AM746048	E1	E117	Muchadeyi, F. C., Eding, H., Simianer, H., Wollny, C. B., Groeneveld, E., & Weigend, S. (2008). Mitochondrial DNA D-loop sequences suggest a Southeast Asian and Indian origin of Zimbabwean village chickens. *Animal genetics* , *39* (6), 615–622. https://doi.org/10.1111/j.1365-2052.2008.01785.x
AM746052	E1	E118	Muchadeyi, F. C., Eding, H., Simianer, H., Wollny, C. B., Groeneveld, E., & Weigend, S. (2008). Mitochondrial DNA D-loop sequences suggest a Southeast Asian and Indian origin of Zimbabwean village chickens. *Animal genetics* , *39* (6), 615–622. https://doi.org/10.1111/j.1365-2052.2008.01785.x
GU561993	E1	E119	Pratap et al. 2010 Direct Submission
EU199926	E1	E120	Silva, P., Guan, X., Ho-Shing, O., Jones, J., Xu, J., Hui, D., Notter, D., & Smith, E. (2009). Mitochondrial DNA-based analysis of genetic variation and relatedness among Sri Lankan indigenous chickens and the Ceylon junglefowl (Gallus lafayetti). *Animal genetics* , *40* (1), 1–9. https://doi.org/10.1111/j.1365-2052.2008.01783.x
HM015611	E1	E121	Dana, N., Megens, H. J., Crooijmans, R. P., Hanotte, O., Mwacharo, J., Groenen, M. A., & van Arendonk, J. A. (2011). East Asian contributions to Dutch traditional and western commercial chickens inferred from mtDNA analysis. *Animal genetics* , *42* (2), 125–133. https://doi.org/10.1111/j.1365-2052.2010.02134.x
HM015613	E3	E122	Dana, N., Megens, H. J., Crooijmans, R. P., Hanotte, O., Mwacharo, J., Groenen, M. A., & van Arendonk, J. A. (2011). East Asian contributions to Dutch traditional and western commercial chickens inferred from mtDNA analysis. *Animal genetics* , *42* (2), 125–133. https://doi.org/10.1111/j.1365-2052.2010.02134.x
HM015615	E1	E123	Dana, N., Megens, H. J., Crooijmans, R. P., Hanotte, O., Mwacharo, J., Groenen, M. A., & van Arendonk, J. A. (2011). East Asian contributions to Dutch traditional and western commercial chickens inferred from mtDNA analysis. *Animal genetics* , *42* (2), 125–133. https://doi.org/10.1111/j.1365-2052.2010.02134.x
HM015618	E1	E124	Dana, N., Megens, H. J., Crooijmans, R. P., Hanotte, O., Mwacharo, J., Groenen, M. A., & van Arendonk, J. A. (2011). East Asian contributions to Dutch traditional and western commercial chickens inferred from mtDNA analysis. *Animal genetics* , *42* (2), 125–133. https://doi.org/10.1111/j.1365-2052.2010.02134.x
HM015620	E1	E125	Dana, N., Megens, H. J., Crooijmans, R. P., Hanotte, O., Mwacharo, J., Groenen, M. A., & van Arendonk, J. A. (2011). East Asian contributions to Dutch traditional and western commercial chickens inferred from mtDNA analysis. *Animal genetics* , *42* (2), 125–133. https://doi.org/10.1111/j.1365-2052.2010.02134.x
AB263948	E1	E127	Wada et al. 2008 Direct Submission
AM746029	E1	E128	Muchadeyi, F. C., Eding, H., Simianer, H., Wollny, C. B., Groeneveld, E., & Weigend, S. (2008). Mitochondrial DNA D-loop sequences suggest a Southeast Asian and Indian origin of Zimbabwean village chickens. *Animal genetics* , *39* (6), 615–622. https://doi.org/10.1111/j.1365-2052.2008.01785.x
AM746043	E1	E129	Muchadeyi, F. C., Eding, H., Simianer, H., Wollny, C. B., Groeneveld, E., & Weigend, S. (2008). Mitochondrial DNA D-loop sequences suggest a Southeast Asian and Indian origin of Zimbabwean village chickens. *Animal genetics* , *39* (6), 615–622. https://doi.org/10.1111/j.1365-2052.2008.01785.x
EU847815	E2	E130	Kanginakudru, S., Metta, M., Jakati, R. D., & Nagaraju, J. (2008). Genetic evidence from Indian red jungle fowl corroborates multiple domestication of modern day chicken. *BMC evolutionary biology* , *8* , 174. https://doi.org/10.1186/1471-2148-8-174
EF535246	E1	E131	Storey, A. A., Ramírez, J. M., Quiroz, D., Burley, D. V., Addison, D. J., Walter, R., Anderson, A. J., Hunt, T. L., Athens, J. S., Huynen, L., & Matisoo-Smith, E. A. (2007). Radiocarbon and DNA evidence for a pre-Columbian introduction of Polynesian chickens to Chile. *Proceedings of the National Academy of Sciences of the United States of America* , *104* (25), 10335–10339. https://doi.org/10.1073/pnas.0703993104

AF512285	F	F01	Liu, Y. P., Wu, G. S., Yao, Y. G., Miao, Y. W., Luikart, G., Baig, M., Beja-Pereira, A., Ding, Z. L., Palanichamy, M. G., & Zhang, Y. P. (2006). Multiple maternal origins of chickens: out of the Asian jungles. *Molecular phylogenetics and evolution* , *38* (1), 12–19. https://doi.org/10.1016/j.ympev.2005.09.014
GU447893	F	F02	Miao, Y.-W., Peng, M.-S., Wu, G.-S., Ouyang, Y.-N., Yang, Z.-Y., Yu, N., Liang, J.-P., Pianchou, G., Beja-Pereira, A., Mitra, B., Palanichamy, M. G., Baig, M., Chaudhuri, T. K., Shen, Y.-Y., Kong, Q.-P., Murphy, R. W., Yao, Y.-G., & Zhang, Y.-P. (2013). Chicken domestication: an updated perspective based on mitochondrial genomes. Heredity, 110(3), 277–282. https://doi.org/10.1038/hdy.2012.83
AF512185	F	F03	Liu, Y. P., Wu, G. S., Yao, Y. G., Miao, Y. W., Luikart, G., Baig, M., Beja-Pereira, A., Ding, Z. L., Palanichamy, M. G., & Zhang, Y. P. (2006). Multiple maternal origins of chickens: out of the Asian jungles. *Molecular phylogenetics and evolution* , *38* (1), 12–19. https://doi.org/10.1016/j.ympev.2005.09.014
AY392213	F	F04	Liu, Y. P., Wu, G. S., Yao, Y. G., Miao, Y. W., Luikart, G., Baig, M., Beja-Pereira, A., Ding, Z. L., Palanichamy, M. G., & Zhang, Y. P. (2006). Multiple maternal origins of chickens: out of the Asian jungles. *Molecular phylogenetics and evolution* , *38* (1), 12–19. https://doi.org/10.1016/j.ympev.2005.09.014
AF512153	F	F05	Liu, Y. P., Wu, G. S., Yao, Y. G., Miao, Y. W., Luikart, G., Baig, M., Beja-Pereira, A., Ding, Z. L., Palanichamy, M. G., & Zhang, Y. P. (2006). Multiple maternal origins of chickens: out of the Asian jungles. *Molecular phylogenetics and evolution* , *38* (1), 12–19. https://doi.org/10.1016/j.ympev.2005.09.014
AF512157	F	F06	Liu, Y. P., Wu, G. S., Yao, Y. G., Miao, Y. W., Luikart, G., Baig, M., Beja-Pereira, A., Ding, Z. L., Palanichamy, M. G., & Zhang, Y. P. (2006). Multiple maternal origins of chickens: out of the Asian jungles. *Molecular phylogenetics and evolution* , *38* (1), 12–19. https://doi.org/10.1016/j.ympev.2005.09.014
GU447992	F	F07	Miao, Y.-W., Peng, M.-S., Wu, G.-S., Ouyang, Y.-N., Yang, Z.-Y., Yu, N., Liang, J.-P., Pianchou, G., Beja-Pereira, A., Mitra, B., Palanichamy, M. G., Baig, M., Chaudhuri, T. K., Shen, Y.-Y., Kong, Q.-P., Murphy, R. W., Yao, Y.-G., & Zhang, Y.-P. (2013). Chicken domestication: an updated perspective based on mitochondrial genomes. Heredity, 110(3), 277–282. https://doi.org/10.1038/hdy.2012.83
D82907	F	F08	Fumihito, A., Miyake, T., Takada, M., Shingu, R., Endo, T., Gojobori, T., Kondo, N., & Ohno, S. (1996). Monophyletic origin and unique dispersal patterns of domestic fowls. *Proceedings of the National Academy of Sciences of the United States of America* , *93* (13), 6792–6795. https://doi.org/10.1073/pnas.93.13.6792
AB009443	F	F09	Miyake 2000 Direct Submission
AY392312	F	F10	Liu, Y. P., Wu, G. S., Yao, Y. G., Miao, Y. W., Luikart, G., Baig, M., Beja-Pereira, A., Ding, Z. L., Palanichamy, M. G., & Zhang, Y. P. (2006). Multiple maternal origins of chickens: out of the Asian jungles. *Molecular phylogenetics and evolution* , *38* (1), 12–19. https://doi.org/10.1016/j.ympev.2005.09.014
AY392328	F	F11	Liu, Y. P., Wu, G. S., Yao, Y. G., Miao, Y. W., Luikart, G., Baig, M., Beja-Pereira, A., Ding, Z. L., Palanichamy, M. G., & Zhang, Y. P. (2006). Multiple maternal origins of chickens: out of the Asian jungles. *Molecular phylogenetics and evolution* , *38* (1), 12–19. https://doi.org/10.1016/j.ympev.2005.09.014
AY392305	F	F12	Liu, Y. P., Wu, G. S., Yao, Y. G., Miao, Y. W., Luikart, G., Baig, M., Beja-Pereira, A., Ding, Z. L., Palanichamy, M. G., & Zhang, Y. P. (2006). Multiple maternal origins of chickens: out of the Asian jungles. *Molecular phylogenetics and evolution* , *38* (1), 12–19. https://doi.org/10.1016/j.ympev.2005.09.014
AY392311	F	F13	Liu, Y. P., Wu, G. S., Yao, Y. G., Miao, Y. W., Luikart, G., Baig, M., Beja-Pereira, A., Ding, Z. L., Palanichamy, M. G., & Zhang, Y. P. (2006). Multiple maternal origins of chickens: out of the Asian jungles. *Molecular phylogenetics and evolution* , *38* (1), 12–19. https://doi.org/10.1016/j.ympev.2005.09.014
AY392309	F	F14	Liu, Y. P., Wu, G. S., Yao, Y. G., Miao, Y. W., Luikart, G., Baig, M., Beja-Pereira, A., Ding, Z. L., Palanichamy, M. G., & Zhang, Y. P. (2006). Multiple maternal origins of chickens: out of the Asian jungles. *Molecular phylogenetics and evolution* , *38* (1), 12–19. https://doi.org/10.1016/j.ympev.2005.09.014
GU447697	F	F15	Miao, Y.-W., Peng, M.-S., Wu, G.-S., Ouyang, Y.-N., Yang, Z.-Y., Yu, N., Liang, J.-P., Pianchou, G., Beja-Pereira, A., Mitra, B., Palanichamy, M. G., Baig, M., Chaudhuri, T. K., Shen, Y.-Y., Kong, Q.-P., Murphy, R. W., Yao, Y.-G., & Zhang, Y.-P. (2013). Chicken domestication: an updated perspective based on mitochondrial genomes. Heredity, 110(3), 277–282. https://doi.org/10.1038/hdy.2012.83
AY392173	F	F16	Liu, Y. P., Wu, G. S., Yao, Y. G., Miao, Y. W., Luikart, G., Baig, M., Beja-Pereira, A., Ding, Z. L., Palanichamy, M. G., & Zhang, Y. P. (2006). Multiple maternal origins of chickens: out of the Asian jungles. *Molecular phylogenetics and evolution* , *38* (1), 12–19. https://doi.org/10.1016/j.ympev.2005.09.014
AY392314	F	F17	Liu, Y. P., Wu, G. S., Yao, Y. G., Miao, Y. W., Luikart, G., Baig, M., Beja-Pereira, A., Ding, Z. L., Palanichamy, M. G., & Zhang, Y. P. (2006). Multiple maternal origins of chickens: out of the Asian jungles. *Molecular phylogenetics and evolution* , *38* (1), 12–19. https://doi.org/10.1016/j.ympev.2005.09.014
GU447606	F	F18	Miao, Y.-W., Peng, M.-S., Wu, G.-S., Ouyang, Y.-N., Yang, Z.-Y., Yu, N., Liang, J.-P., Pianchou, G., Beja-Pereira, A., Mitra, B., Palanichamy, M. G., Baig, M., Chaudhuri, T. K., Shen, Y.-Y., Kong, Q.-P., Murphy, R. W., Yao, Y.-G., & Zhang, Y.-P. (2013). Chicken domestication: an updated perspective based on mitochondrial genomes. Heredity, 110(3), 277–282. https://doi.org/10.1038/hdy.2012.83
GU448935	F	F19	Miao, Y.-W., Peng, M.-S., Wu, G.-S., Ouyang, Y.-N., Yang, Z.-Y., Yu, N., Liang, J.-P., Pianchou, G., Beja-Pereira, A., Mitra, B., Palanichamy, M. G., Baig, M., Chaudhuri, T. K., Shen, Y.-Y., Kong, Q.-P., Murphy, R. W., Yao, Y.-G., & Zhang, Y.-P. (2013). Chicken domestication: an updated perspective based on mitochondrial genomes. Heredity, 110(3), 277–282. https://doi.org/10.1038/hdy.2012.83
GU447672	F	F20	Miao, Y.-W., Peng, M.-S., Wu, G.-S., Ouyang, Y.-N., Yang, Z.-Y., Yu, N., Liang, J.-P., Pianchou, G., Beja-Pereira, A., Mitra, B., Palanichamy, M. G., Baig, M., Chaudhuri, T. K., Shen, Y.-Y., Kong, Q.-P., Murphy, R. W., Yao, Y.-G., & Zhang, Y.-P. (2013). Chicken domestication: an updated perspective based on mitochondrial genomes. Heredity, 110(3), 277–282. https://doi.org/10.1038/hdy.2012.83
GU447621	F	F21	Miao, Y.-W., Peng, M.-S., Wu, G.-S., Ouyang, Y.-N., Yang, Z.-Y., Yu, N., Liang, J.-P., Pianchou, G., Beja-Pereira, A., Mitra, B., Palanichamy, M. G., Baig, M., Chaudhuri, T. K., Shen, Y.-Y., Kong, Q.-P., Murphy, R. W., Yao, Y.-G., & Zhang, Y.-P. (2013). Chicken domestication: an updated perspective based on mitochondrial genomes. Heredity, 110(3), 277–282. https://doi.org/10.1038/hdy.2012.83
GU447612	F	F22	Miao, Y.-W., Peng, M.-S., Wu, G.-S., Ouyang, Y.-N., Yang, Z.-Y., Yu, N., Liang, J.-P., Pianchou, G., Beja-Pereira, A., Mitra, B., Palanichamy, M. G., Baig, M., Chaudhuri, T. K., Shen, Y.-Y., Kong, Q.-P., Murphy, R. W., Yao, Y.-G., & Zhang, Y.-P. (2013). Chicken domestication: an updated perspective based on mitochondrial genomes. Heredity, 110(3), 277–282. https://doi.org/10.1038/hdy.2012.83
GU448497	F	F23	Miao, Y.-W., Peng, M.-S., Wu, G.-S., Ouyang, Y.-N., Yang, Z.-Y., Yu, N., Liang, J.-P., Pianchou, G., Beja-Pereira, A., Mitra, B., Palanichamy, M. G., Baig, M., Chaudhuri, T. K., Shen, Y.-Y., Kong, Q.-P., Murphy, R. W., Yao, Y.-G., & Zhang, Y.-P. (2013). Chicken domestication: an updated perspective based on mitochondrial genomes. Heredity, 110(3), 277–282. https://doi.org/10.1038/hdy.2012.83
GU447670	F	F24	Miao, Y.-W., Peng, M.-S., Wu, G.-S., Ouyang, Y.-N., Yang, Z.-Y., Yu, N., Liang, J.-P., Pianchou, G., Beja-Pereira, A., Mitra, B., Palanichamy, M. G., Baig, M., Chaudhuri, T. K., Shen, Y.-Y., Kong, Q.-P., Murphy, R. W., Yao, Y.-G., & Zhang, Y.-P. (2013). Chicken domestication: an updated perspective based on mitochondrial genomes. Heredity, 110(3), 277–282. https://doi.org/10.1038/hdy.2012.83
GU448092	F	F25	Miao, Y.-W., Peng, M.-S., Wu, G.-S., Ouyang, Y.-N., Yang, Z.-Y., Yu, N., Liang, J.-P., Pianchou, G., Beja-Pereira, A., Mitra, B., Palanichamy, M. G., Baig, M., Chaudhuri, T. K., Shen, Y.-Y., Kong, Q.-P., Murphy, R. W., Yao, Y.-G., & Zhang, Y.-P. (2013). Chicken domestication: an updated perspective based on mitochondrial genomes. Heredity, 110(3), 277–282. https://doi.org/10.1038/hdy.2012.83
GU448659	F	F26	Miao, Y.-W., Peng, M.-S., Wu, G.-S., Ouyang, Y.-N., Yang, Z.-Y., Yu, N., Liang, J.-P., Pianchou, G., Beja-Pereira, A., Mitra, B., Palanichamy, M. G., Baig, M., Chaudhuri, T. K., Shen, Y.-Y., Kong, Q.-P., Murphy, R. W., Yao, Y.-G., & Zhang, Y.-P. (2013). Chicken domestication: an updated perspective based on mitochondrial genomes. Heredity, 110(3), 277–282. https://doi.org/10.1038/hdy.2012.83
GU447738	F	F27	Miao, Y.-W., Peng, M.-S., Wu, G.-S., Ouyang, Y.-N., Yang, Z.-Y., Yu, N., Liang, J.-P., Pianchou, G., Beja-Pereira, A., Mitra, B., Palanichamy, M. G., Baig, M., Chaudhuri, T. K., Shen, Y.-Y., Kong, Q.-P., Murphy, R. W., Yao, Y.-G., & Zhang, Y.-P. (2013). Chicken domestication: an updated perspective based on mitochondrial genomes. Heredity, 110(3), 277–282. https://doi.org/10.1038/hdy.2012.83
GU447906	F	F28	Miao, Y.-W., Peng, M.-S., Wu, G.-S., Ouyang, Y.-N., Yang, Z.-Y., Yu, N., Liang, J.-P., Pianchou, G., Beja-Pereira, A., Mitra, B., Palanichamy, M. G., Baig, M., Chaudhuri, T. K., Shen, Y.-Y., Kong, Q.-P., Murphy, R. W., Yao, Y.-G., & Zhang, Y.-P. (2013). Chicken domestication: an updated perspective based on mitochondrial genomes. Heredity, 110(3), 277–282. https://doi.org/10.1038/hdy.2012.83
GU447601	F	F29	Miao, Y.-W., Peng, M.-S., Wu, G.-S., Ouyang, Y.-N., Yang, Z.-Y., Yu, N., Liang, J.-P., Pianchou, G., Beja-Pereira, A., Mitra, B., Palanichamy, M. G., Baig, M., Chaudhuri, T. K., Shen, Y.-Y., Kong, Q.-P., Murphy, R. W., Yao, Y.-G., & Zhang, Y.-P. (2013). Chicken domestication: an updated perspective based on mitochondrial genomes. Heredity, 110(3), 277–282. https://doi.org/10.1038/hdy.2012.83
HM462182	F	F30	Berthouly-Salazar, C., Rognon, X., Van, T., Gély, M., Chi, C. V., Tixier-Boichard, M., Bed'Hom, B., Bruneau, N., Verrier, E., Maillard, J. C., & Michaux, J. R. (2010). Vietnamese chickens: a gate towards Asian genetic diversity. *BMC genetics* , *11* , 53. https://doi.org/10.1186/1471-2156-11-53
HM015609	F	F32	Dana, N., Megens, H. J., Crooijmans, R. P., Hanotte, O., Mwacharo, J., Groenen, M. A., & van Arendonk, J. A. (2011). East Asian contributions to Dutch traditional and western commercial chickens inferred from mtDNA analysis. *Animal genetics* , *42* (2), 125–133. https://doi.org/10.1111/j.1365-2052.2010.02134.x
GU447638	F	F33	Miao, Y.-W., Peng, M.-S., Wu, G.-S., Ouyang, Y.-N., Yang, Z.-Y., Yu, N., Liang, J.-P., Pianchou, G., Beja-Pereira, A., Mitra, B., Palanichamy, M. G., Baig, M., Chaudhuri, T. K., Shen, Y.-Y., Kong, Q.-P., Murphy, R. W., Yao, Y.-G., & Zhang, Y.-P. (2013). Chicken domestication: an updated perspective based on mitochondrial genomes. Heredity, 110(3), 277–282. https://doi.org/10.1038/hdy.2012.83
HM462142	F	F34	Berthouly-Salazar, C., Rognon, X., Van, T., Gély, M., Chi, C. V., Tixier-Boichard, M., Bed'Hom, B., Bruneau, N., Verrier, E., Maillard, J. C., & Michaux, J. R. (2010). Vietnamese chickens: a gate towards Asian genetic diversity. *BMC genetics* , *11* , 53. https://doi.org/10.1186/1471-2156-11-53
GU448505	G	G01	Miao, Y.-W., Peng, M.-S., Wu, G.-S., Ouyang, Y.-N., Yang, Z.-Y., Yu, N., Liang, J.-P., Pianchou, G., Beja-Pereira, A., Mitra, B., Palanichamy, M. G., Baig, M., Chaudhuri, T. K., Shen, Y.-Y., Kong, Q.-P., Murphy, R. W., Yao, Y.-G., & Zhang, Y.-P. (2013). Chicken domestication: an updated perspective based on mitochondrial genomes. Heredity, 110(3), 277–282. https://doi.org/10.1038/hdy.2012.83
AF512112	G	G02	Liu, Y. P., Wu, G. S., Yao, Y. G., Miao, Y. W., Luikart, G., Baig, M., Beja-Pereira, A., Ding, Z. L., Palanichamy, M. G., & Zhang, Y. P. (2006). Multiple maternal origins of chickens: out of the Asian jungles. *Molecular phylogenetics and evolution* , *38* (1), 12–19. https://doi.org/10.1016/j.ympev.2005.09.014
AF512146	G	G03	Liu, Y. P., Wu, G. S., Yao, Y. G., Miao, Y. W., Luikart, G., Baig, M., Beja-Pereira, A., Ding, Z. L., Palanichamy, M. G., & Zhang, Y. P. (2006). Multiple maternal origins of chickens: out of the Asian jungles. *Molecular phylogenetics and evolution* , *38* (1), 12–19. https://doi.org/10.1016/j.ympev.2005.09.014
AF512249	G	G04	Liu, Y. P., Wu, G. S., Yao, Y. G., Miao, Y. W., Luikart, G., Baig, M., Beja-Pereira, A., Ding, Z. L., Palanichamy, M. G., & Zhang, Y. P. (2006). Multiple maternal origins of chickens: out of the Asian jungles. *Molecular phylogenetics and evolution* , *38* (1), 12–19. https://doi.org/10.1016/j.ympev.2005.09.014
AF512324	G	G05	Liu, Y. P., Wu, G. S., Yao, Y. G., Miao, Y. W., Luikart, G., Baig, M., Beja-Pereira, A., Ding, Z. L., Palanichamy, M. G., & Zhang, Y. P. (2006). Multiple maternal origins of chickens: out of the Asian jungles. *Molecular phylogenetics and evolution* , *38* (1), 12–19. https://doi.org/10.1016/j.ympev.2005.09.014
AY392220	G	G07	Liu, Y. P., Wu, G. S., Yao, Y. G., Miao, Y. W., Luikart, G., Baig, M., Beja-Pereira, A., Ding, Z. L., Palanichamy, M. G., & Zhang, Y. P. (2006). Multiple maternal origins of chickens: out of the Asian jungles. *Molecular phylogenetics and evolution* , *38* (1), 12–19. https://doi.org/10.1016/j.ympev.2005.09.014
AY392346	G	G08	Liu, Y. P., Wu, G. S., Yao, Y. G., Miao, Y. W., Luikart, G., Baig, M., Beja-Pereira, A., Ding, Z. L., Palanichamy, M. G., & Zhang, Y. P. (2006). Multiple maternal origins of chickens: out of the Asian jungles. *Molecular phylogenetics and evolution* , *38* (1), 12–19. https://doi.org/10.1016/j.ympev.2005.09.014

AY392232	G	G09	Liu, Y. P., Wu, G. S., Yao, Y. G., Miao, Y. W., Luikart, G., Baig, M., Beja-Pereira, A., Ding, Z. L., Palanichamy, M. G., & Zhang, Y. P. (2006). Multiple maternal origins of chickens: out of the Asian jungles. *Molecular phylogenetics and evolution* , *38* (1), 12–19. https://doi.org/10.1016/j.ympev.2005.09.014
AY392263	G	G10	Liu, Y. P., Wu, G. S., Yao, Y. G., Miao, Y. W., Luikart, G., Baig, M., Beja-Pereira, A., Ding, Z. L., Palanichamy, M. G., & Zhang, Y. P. (2006). Multiple maternal origins of chickens: out of the Asian jungles. *Molecular phylogenetics and evolution* , *38* (1), 12–19. https://doi.org/10.1016/j.ympev.2005.09.014
AY392241	G	G11	Liu, Y. P., Wu, G. S., Yao, Y. G., Miao, Y. W., Luikart, G., Baig, M., Beja-Pereira, A., Ding, Z. L., Palanichamy, M. G., & Zhang, Y. P. (2006). Multiple maternal origins of chickens: out of the Asian jungles. *Molecular phylogenetics and evolution* , *38* (1), 12–19. https://doi.org/10.1016/j.ympev.2005.09.014
AY392251	G	G12	Liu, Y. P., Wu, G. S., Yao, Y. G., Miao, Y. W., Luikart, G., Baig, M., Beja-Pereira, A., Ding, Z. L., Palanichamy, M. G., & Zhang, Y. P. (2006). Multiple maternal origins of chickens: out of the Asian jungles. *Molecular phylogenetics and evolution* , *38* (1), 12–19. https://doi.org/10.1016/j.ympev.2005.09.014
AY392260	G	G13	Liu, Y. P., Wu, G. S., Yao, Y. G., Miao, Y. W., Luikart, G., Baig, M., Beja-Pereira, A., Ding, Z. L., Palanichamy, M. G., & Zhang, Y. P. (2006). Multiple maternal origins of chickens: out of the Asian jungles. *Molecular phylogenetics and evolution* , *38* (1), 12–19. https://doi.org/10.1016/j.ympev.2005.09.014
AY392215	G	G14	Liu, Y. P., Wu, G. S., Yao, Y. G., Miao, Y. W., Luikart, G., Baig, M., Beja-Pereira, A., Ding, Z. L., Palanichamy, M. G., & Zhang, Y. P. (2006). Multiple maternal origins of chickens: out of the Asian jungles. *Molecular phylogenetics and evolution* , *38* (1), 12–19. https://doi.org/10.1016/j.ympev.2005.09.014
AY392239	G	G15	Liu, Y. P., Wu, G. S., Yao, Y. G., Miao, Y. W., Luikart, G., Baig, M., Beja-Pereira, A., Ding, Z. L., Palanichamy, M. G., & Zhang, Y. P. (2006). Multiple maternal origins of chickens: out of the Asian jungles. *Molecular phylogenetics and evolution* , *38* (1), 12–19. https://doi.org/10.1016/j.ympev.2005.09.014
AY392209	G	G16	Liu, Y. P., Wu, G. S., Yao, Y. G., Miao, Y. W., Luikart, G., Baig, M., Beja-Pereira, A., Ding, Z. L., Palanichamy, M. G., & Zhang, Y. P. (2006). Multiple maternal origins of chickens: out of the Asian jungles. *Molecular phylogenetics and evolution* , *38* (1), 12–19. https://doi.org/10.1016/j.ympev.2005.09.014
AY392351	G	G17	Liu, Y. P., Wu, G. S., Yao, Y. G., Miao, Y. W., Luikart, G., Baig, M., Beja-Pereira, A., Ding, Z. L., Palanichamy, M. G., & Zhang, Y. P. (2006). Multiple maternal origins of chickens: out of the Asian jungles. *Molecular phylogenetics and evolution* , *38* (1), 12–19. https://doi.org/10.1016/j.ympev.2005.09.014
AY392282	G	G18	Liu, Y. P., Wu, G. S., Yao, Y. G., Miao, Y. W., Luikart, G., Baig, M., Beja-Pereira, A., Ding, Z. L., Palanichamy, M. G., & Zhang, Y. P. (2006). Multiple maternal origins of chickens: out of the Asian jungles. *Molecular phylogenetics and evolution* , *38* (1), 12–19. https://doi.org/10.1016/j.ympev.2005.09.014
AY392292	G	G19	Liu, Y. P., Wu, G. S., Yao, Y. G., Miao, Y. W., Luikart, G., Baig, M., Beja-Pereira, A., Ding, Z. L., Palanichamy, M. G., & Zhang, Y. P. (2006). Multiple maternal origins of chickens: out of the Asian jungles. *Molecular phylogenetics and evolution* , *38* (1), 12–19. https://doi.org/10.1016/j.ympev.2005.09.014
AY392290	G	G20	Liu, Y. P., Wu, G. S., Yao, Y. G., Miao, Y. W., Luikart, G., Baig, M., Beja-Pereira, A., Ding, Z. L., Palanichamy, M. G., & Zhang, Y. P. (2006). Multiple maternal origins of chickens: out of the Asian jungles. *Molecular phylogenetics and evolution* , *38* (1), 12–19. https://doi.org/10.1016/j.ympev.2005.09.014
GU448146	G	G21	Miao, Y.-W., Peng, M.-S., Wu, G.-S., Ouyang, Y.-N., Yang, Z.-Y., Yu, N., Liang, J.-P., Pianchou, G., Beja-Pereira, A., Mitra, B., Palanichamy, M. G., Baig, M., Chaudhuri, T. K., Shen, Y.-Y., Kong, Q.-P., Murphy, R. W., Yao, Y.-G., & Zhang, Y.-P. (2013). Chicken domestication: an updated perspective based on mitochondrial genomes. Heredity, 110(3), 277–282. https://doi.org/10.1038/hdy.2012.83
AY392335	G	G22	Liu, Y. P., Wu, G. S., Yao, Y. G., Miao, Y. W., Luikart, G., Baig, M., Beja-Pereira, A., Ding, Z. L., Palanichamy, M. G., & Zhang, Y. P. (2006). Multiple maternal origins of chickens: out of the Asian jungles. *Molecular phylogenetics and evolution* , *38* (1), 12–19. https://doi.org/10.1016/j.ympev.2005.09.014
GU448144	G	G23	Miao, Y.-W., Peng, M.-S., Wu, G.-S., Ouyang, Y.-N., Yang, Z.-Y., Yu, N., Liang, J.-P., Pianchou, G., Beja-Pereira, A., Mitra, B., Palanichamy, M. G., Baig, M., Chaudhuri, T. K., Shen, Y.-Y., Kong, Q.-P., Murphy, R. W., Yao, Y.-G., & Zhang, Y.-P. (2013). Chicken domestication: an updated perspective based on mitochondrial genomes. Heredity, 110(3), 277–282. https://doi.org/10.1038/hdy.2012.83
AY465997	G	G24	Liu et al. 2003 Direct Submission
GU447577	G	G25	Miao, Y.-W., Peng, M.-S., Wu, G.-S., Ouyang, Y.-N., Yang, Z.-Y., Yu, N., Liang, J.-P., Pianchou, G., Beja-Pereira, A., Mitra, B., Palanichamy, M. G., Baig, M., Chaudhuri, T. K., Shen, Y.-Y., Kong, Q.-P., Murphy, R. W., Yao, Y.-G., & Zhang, Y.-P. (2013). Chicken domestication: an updated perspective based on mitochondrial genomes. Heredity, 110(3), 277–282. https://doi.org/10.1038/hdy.2012.83
GU448101	G	G26	Miao, Y.-W., Peng, M.-S., Wu, G.-S., Ouyang, Y.-N., Yang, Z.-Y., Yu, N., Liang, J.-P., Pianchou, G., Beja-Pereira, A., Mitra, B., Palanichamy, M. G., Baig, M., Chaudhuri, T. K., Shen, Y.-Y., Kong, Q.-P., Murphy, R. W., Yao, Y.-G., & Zhang, Y.-P. (2013). Chicken domestication: an updated perspective based on mitochondrial genomes. Heredity, 110(3), 277–282. https://doi.org/10.1038/hdy.2012.83
GU447732	G	G27	Miao, Y.-W., Peng, M.-S., Wu, G.-S., Ouyang, Y.-N., Yang, Z.-Y., Yu, N., Liang, J.-P., Pianchou, G., Beja-Pereira, A., Mitra, B., Palanichamy, M. G., Baig, M., Chaudhuri, T. K., Shen, Y.-Y., Kong, Q.-P., Murphy, R. W., Yao, Y.-G., & Zhang, Y.-P. (2013). Chicken domestication: an updated perspective based on mitochondrial genomes. Heredity, 110(3), 277–282. https://doi.org/10.1038/hdy.2012.83
GU448341	G	G28	Miao, Y.-W., Peng, M.-S., Wu, G.-S., Ouyang, Y.-N., Yang, Z.-Y., Yu, N., Liang, J.-P., Pianchou, G., Beja-Pereira, A., Mitra, B., Palanichamy, M. G., Baig, M., Chaudhuri, T. K., Shen, Y.-Y., Kong, Q.-P., Murphy, R. W., Yao, Y.-G., & Zhang, Y.-P. (2013). Chicken domestication: an updated perspective based on mitochondrial genomes. Heredity, 110(3), 277–282. https://doi.org/10.1038/hdy.2012.83
GU447683	G	G29	Miao, Y.-W., Peng, M.-S., Wu, G.-S., Ouyang, Y.-N., Yang, Z.-Y., Yu, N., Liang, J.-P., Pianchou, G., Beja-Pereira, A., Mitra, B., Palanichamy, M. G., Baig, M., Chaudhuri, T. K., Shen, Y.-Y., Kong, Q.-P., Murphy, R. W., Yao, Y.-G., & Zhang, Y.-P. (2013). Chicken domestication: an updated perspective based on mitochondrial genomes. Heredity, 110(3), 277–282. https://doi.org/10.1038/hdy.2012.83
GU448340	G	G30	Miao, Y.-W., Peng, M.-S., Wu, G.-S., Ouyang, Y.-N., Yang, Z.-Y., Yu, N., Liang, J.-P., Pianchou, G., Beja-Pereira, A., Mitra, B., Palanichamy, M. G., Baig, M., Chaudhuri, T. K., Shen, Y.-Y., Kong, Q.-P., Murphy, R. W., Yao, Y.-G., & Zhang, Y.-P. (2013). Chicken domestication: an updated perspective based on mitochondrial genomes. Heredity, 110(3), 277–282. https://doi.org/10.1038/hdy.2012.83
GU448338	G	G31	Miao, Y.-W., Peng, M.-S., Wu, G.-S., Ouyang, Y.-N., Yang, Z.-Y., Yu, N., Liang, J.-P., Pianchou, G., Beja-Pereira, A., Mitra, B., Palanichamy, M. G., Baig, M., Chaudhuri, T. K., Shen, Y.-Y., Kong, Q.-P., Murphy, R. W., Yao, Y.-G., & Zhang, Y.-P. (2013). Chicken domestication: an updated perspective based on mitochondrial genomes. Heredity, 110(3), 277–282. https://doi.org/10.1038/hdy.2012.83
GU448294	G	G32	Miao, Y.-W., Peng, M.-S., Wu, G.-S., Ouyang, Y.-N., Yang, Z.-Y., Yu, N., Liang, J.-P., Pianchou, G., Beja-Pereira, A., Mitra, B., Palanichamy, M. G., Baig, M., Chaudhuri, T. K., Shen, Y.-Y., Kong, Q.-P., Murphy, R. W., Yao, Y.-G., & Zhang, Y.-P. (2013). Chicken domestication: an updated perspective based on mitochondrial genomes. Heredity, 110(3), 277–282. https://doi.org/10.1038/hdy.2012.83
GU448290	G	G33	Miao, Y.-W., Peng, M.-S., Wu, G.-S., Ouyang, Y.-N., Yang, Z.-Y., Yu, N., Liang, J.-P., Pianchou, G., Beja-Pereira, A., Mitra, B., Palanichamy, M. G., Baig, M., Chaudhuri, T. K., Shen, Y.-Y., Kong, Q.-P., Murphy, R. W., Yao, Y.-G., & Zhang, Y.-P. (2013). Chicken domestication: an updated perspective based on mitochondrial genomes. Heredity, 110(3), 277–282. https://doi.org/10.1038/hdy.2012.83
GU448893	G	G34	Miao, Y.-W., Peng, M.-S., Wu, G.-S., Ouyang, Y.-N., Yang, Z.-Y., Yu, N., Liang, J.-P., Pianchou, G., Beja-Pereira, A., Mitra, B., Palanichamy, M. G., Baig, M., Chaudhuri, T. K., Shen, Y.-Y., Kong, Q.-P., Murphy, R. W., Yao, Y.-G., & Zhang, Y.-P. (2013). Chicken domestication: an updated perspective based on mitochondrial genomes. Heredity, 110(3), 277–282. https://doi.org/10.1038/hdy.2012.83
EU199927	G	G35	Silva, P., Guan, X., Ho-Shing, O., Jones, J., Xu, J., Hui, D., Notter, D., & Smith, E. (2009). Mitochondrial DNA-based analysis of genetic variation and relatedness among Sri Lankan indigenous chickens and the Ceylon junglefowl (Gallus lafayetti). *Animal genetics* , *40* (1), 1–9. https://doi.org/10.1111/j.1365-2052.2008.01783.x
EU199929	G	G36	Silva, P., Guan, X., Ho-Shing, O., Jones, J., Xu, J., Hui, D., Notter, D., & Smith, E. (2009). Mitochondrial DNA-based analysis of genetic variation and relatedness among Sri Lankan indigenous chickens and the Ceylon junglefowl (Gallus lafayetti). *Animal genetics* , *40* (1), 1–9. https://doi.org/10.1111/j.1365-2052.2008.01783.x
EU199934	G	G37	Silva, P., Guan, X., Ho-Shing, O., Jones, J., Xu, J., Hui, D., Notter, D., & Smith, E. (2009). Mitochondrial DNA-based analysis of genetic variation and relatedness among Sri Lankan indigenous chickens and the Ceylon junglefowl (Gallus lafayetti). *Animal genetics* , *40* (1), 1–9. https://doi.org/10.1111/j.1365-2052.2008.01783.x
EU199933	G	G38	Silva, P., Guan, X., Ho-Shing, O., Jones, J., Xu, J., Hui, D., Notter, D., & Smith, E. (2009). Mitochondrial DNA-based analysis of genetic variation and relatedness among Sri Lankan indigenous chickens and the Ceylon junglefowl (Gallus lafayetti). *Animal genetics* , *40* (1), 1–9. https://doi.org/10.1111/j.1365-2052.2008.01783.x
EU199930	G	G39	Silva, P., Guan, X., Ho-Shing, O., Jones, J., Xu, J., Hui, D., Notter, D., & Smith, E. (2009). Mitochondrial DNA-based analysis of genetic variation and relatedness among Sri Lankan indigenous chickens and the Ceylon junglefowl (Gallus lafayetti). *Animal genetics* , *40* (1), 1–9. https://doi.org/10.1111/j.1365-2052.2008.01783.x
EU199932	G	G40	Silva, P., Guan, X., Ho-Shing, O., Jones, J., Xu, J., Hui, D., Notter, D., & Smith, E. (2009). Mitochondrial DNA-based analysis of genetic variation and relatedness among Sri Lankan indigenous chickens and the Ceylon junglefowl (Gallus lafayetti). *Animal genetics* , *40* (1), 1–9. https://doi.org/10.1111/j.1365-2052.2008.01783.x
EU199935	G	G41	Silva, P., Guan, X., Ho-Shing, O., Jones, J., Xu, J., Hui, D., Notter, D., & Smith, E. (2009). Mitochondrial DNA-based analysis of genetic variation and relatedness among Sri Lankan indigenous chickens and the Ceylon junglefowl (Gallus lafayetti). *Animal genetics* , *40* (1), 1–9. https://doi.org/10.1111/j.1365-2052.2008.01783.x
GU448160	G	G42	Miao, Y.-W., Peng, M.-S., Wu, G.-S., Ouyang, Y.-N., Yang, Z.-Y., Yu, N., Liang, J.-P., Pianchou, G., Beja-Pereira, A., Mitra, B., Palanichamy, M. G., Baig, M., Chaudhuri, T. K., Shen, Y.-Y., Kong, Q.-P., Murphy, R. W., Yao, Y.-G., & Zhang, Y.-P. (2013). Chicken domestication: an updated perspective based on mitochondrial genomes. Heredity, 110(3), 277–282. https://doi.org/10.1038/hdy.2012.83
GU447822	G	G43	Miao, Y.-W., Peng, M.-S., Wu, G.-S., Ouyang, Y.-N., Yang, Z.-Y., Yu, N., Liang, J.-P., Pianchou, G., Beja-Pereira, A., Mitra, B., Palanichamy, M. G., Baig, M., Chaudhuri, T. K., Shen, Y.-Y., Kong, Q.-P., Murphy, R. W., Yao, Y.-G., & Zhang, Y.-P. (2013). Chicken domestication: an updated perspective based on mitochondrial genomes. Heredity, 110(3), 277–282. https://doi.org/10.1038/hdy.2012.83
GU447861	G	G44	Miao, Y.-W., Peng, M.-S., Wu, G.-S., Ouyang, Y.-N., Yang, Z.-Y., Yu, N., Liang, J.-P., Pianchou, G., Beja-Pereira, A., Mitra, B., Palanichamy, M. G., Baig, M., Chaudhuri, T. K., Shen, Y.-Y., Kong, Q.-P., Murphy, R. W., Yao, Y.-G., & Zhang, Y.-P. (2013). Chicken domestication: an updated perspective based on mitochondrial genomes. Heredity, 110(3), 277–282. https://doi.org/10.1038/hdy.2012.83
GU447864	G	G45	Miao, Y.-W., Peng, M.-S., Wu, G.-S., Ouyang, Y.-N., Yang, Z.-Y., Yu, N., Liang, J.-P., Pianchou, G., Beja-Pereira, A., Mitra, B., Palanichamy, M. G., Baig, M., Chaudhuri, T. K., Shen, Y.-Y., Kong, Q.-P., Murphy, R. W., Yao, Y.-G., & Zhang, Y.-P. (2013). Chicken domestication: an updated perspective based on mitochondrial genomes. Heredity, 110(3), 277–282. https://doi.org/10.1038/hdy.2012.83
GU448139	G	G46	Miao, Y.-W., Peng, M.-S., Wu, G.-S., Ouyang, Y.-N., Yang, Z.-Y., Yu, N., Liang, J.-P., Pianchou, G., Beja-Pereira, A., Mitra, B., Palanichamy, M. G., Baig, M., Chaudhuri, T. K., Shen, Y.-Y., Kong, Q.-P., Murphy, R. W., Yao, Y.-G., & Zhang, Y.-P. (2013). Chicken domestication: an updated perspective based on mitochondrial genomes. Heredity, 110(3), 277–282. https://doi.org/10.1038/hdy.2012.83
HM462175	G	G49	Berthouly-Salazar, C., Rognon, X., Van, T., Gély, M., Chi, C. V., Tixier-Boichard, M., Bed'Hom, B., Bruneau, N., Verrier, E., Maillard, J. C., & Michaux, J. R. (2010). Vietnamese chickens: a gate towards Asian genetic diversity. *BMC genetics* , *11* , 53. https://doi.org/10.1186/1471-2156-11-53
AY392185	H	H01	Liu, Y. P., Wu, G. S., Yao, Y. G., Miao, Y. W., Luikart, G., Baig, M., Beja-Pereira, A., Ding, Z. L., Palanichamy, M. G., & Zhang, Y. P. (2006). Multiple maternal origins of chickens: out of the Asian jungles. *Molecular phylogenetics and evolution* , *38* (1), 12–19. https://doi.org/10.1016/j.ympev.2005.09.014

GU447859	H	H02	Miao, Y.-W., Peng, M.-S., Wu, G.-S., Ouyang, Y.-N., Yang, Z.-Y., Yu, N., Liang, J.-P., Pianchou, G., Beja-Pereira, A., Mitra, B., Palanichamy, M. G., Baig, M., Chaudhuri, T. K., Shen, Y.-Y., Kong, Q.-P., Murphy, R. W., Yao, Y.-G., & Zhang, Y.-P. (2013). Chicken domestication: an updated perspective based on mitochondrial genomes. Heredity, 110(3), 277–282. https://doi.org/10.1038/hdy.2012.83
AY392176	H	H03	Liu, Y. P., Wu, G. S., Yao, Y. G., Miao, Y. W., Luikart, G., Baig, M., Beja-Pereira, A., Ding, Z. L., Palanichamy, M. G., & Zhang, Y. P. (2006). Multiple maternal origins of chickens: out of the Asian jungles. *Molecular phylogenetics and evolution* , *38* (1), 12–19. https://doi.org/10.1016/j.ympev.2005.09.014
AB098646	H	H04	Komiyama, T., Ikeo, K., & Gojobori, T. (2003). Where is the origin of the Japanese gamecocks?. *Gene* , *317* (1-2), 195–202. https://doi.org/10.1016/s0378-1119(03)00703-0
AB098639	H	H05	Komiyama, T., Ikeo, K., & Gojobori, T. (2003). Where is the origin of the Japanese gamecocks?. *Gene* , *317* (1-2), 195–202. https://doi.org/10.1016/s0378-1119(03)00703-0
AB098662	H	H06	Komiyama, T., Ikeo, K., & Gojobori, T. (2003). Where is the origin of the Japanese gamecocks?. *Gene* , *317* (1-2), 195–202. https://doi.org/10.1016/s0378-1119(03)00703-0
AB098638	H	H07	Komiyama, T., Ikeo, K., & Gojobori, T. (2003). Where is the origin of the Japanese gamecocks?. *Gene* , *317* (1-2), 195–202. https://doi.org/10.1016/s0378-1119(03)00703-0
GU448374	I		Miao, Y.-W., Peng, M.-S., Wu, G.-S., Ouyang, Y.-N., Yang, Z.-Y., Yu, N., Liang, J.-P., Pianchou, G., Beja-Pereira, A., Mitra, B., Palanichamy, M. G., Baig, M., Chaudhuri, T. K., Shen, Y.-Y., Kong, Q.-P., Murphy, R. W., Yao, Y.-G., & Zhang, Y.-P. (2013). Chicken domestication: an updated perspective based on mitochondrial genomes. Heredity, 110(3), 277–282. https://doi.org/10.1038/hdy.2012.83
GU448603	W		Miao, Y.-W., Peng, M.-S., Wu, G.-S., Ouyang, Y.-N., Yang, Z.-Y., Yu, N., Liang, J.-P., Pianchou, G., Beja-Pereira, A., Mitra, B., Palanichamy, M. G., Baig, M., Chaudhuri, T. K., Shen, Y.-Y., Kong, Q.-P., Murphy, R. W., Yao, Y.-G., & Zhang, Y.-P. (2013). Chicken domestication: an updated perspective based on mitochondrial genomes. Heredity, 110(3), 277–282. https://doi.org/10.1038/hdy.2012.83
GU447597	X		Miao, Y.-W., Peng, M.-S., Wu, G.-S., Ouyang, Y.-N., Yang, Z.-Y., Yu, N., Liang, J.-P., Pianchou, G., Beja-Pereira, A., Mitra, B., Palanichamy, M. G., Baig, M., Chaudhuri, T. K., Shen, Y.-Y., Kong, Q.-P., Murphy, R. W., Yao, Y.-G., & Zhang, Y.-P. (2013). Chicken domestication: an updated perspective based on mitochondrial genomes. Heredity, 110(3), 277–282. https://doi.org/10.1038/hdy.2012.83
GU447596	Y		Miao, Y.-W., Peng, M.-S., Wu, G.-S., Ouyang, Y.-N., Yang, Z.-Y., Yu, N., Liang, J.-P., Pianchou, G., Beja-Pereira, A., Mitra, B., Palanichamy, M. G., Baig, M., Chaudhuri, T. K., Shen, Y.-Y., Kong, Q.-P., Murphy, R. W., Yao, Y.-G., & Zhang, Y.-P. (2013). Chicken domestication: an updated perspective based on mitochondrial genomes. Heredity, 110(3), 277–282. https://doi.org/10.1038/hdy.2012.83
GU447643	Z		Miao, Y.-W., Peng, M.-S., Wu, G.-S., Ouyang, Y.-N., Yang, Z.-Y., Yu, N., Liang, J.-P., Pianchou, G., Beja-Pereira, A., Mitra, B., Palanichamy, M. G., Baig, M., Chaudhuri, T. K., Shen, Y.-Y., Kong, Q.-P., Murphy, R. W., Yao, Y.-G., & Zhang, Y.-P. (2013). Chicken domestication: an updated perspective based on mitochondrial genomes. Heredity, 110(3), 277–282. https://doi.org/10.1038/hdy.2012.83
**Ancient references**			
KF753251	E1	E01	Girdland Flink, L., Allen, R., Barnett, R., Malmström, H., Peters, J., Eriksson, J., Andersson, L., Dobney, K., & Larson, G. (2014). Establishing the validity of domestication genes using DNA from ancient chickens. *Proceedings of the National Academy of Sciences* , *111* (17), 6184–6189. https://doi.org/10.1073/pnas.1308939110
KF753252	E1	E01	Girdland Flink, L., Allen, R., Barnett, R., Malmström, H., Peters, J., Eriksson, J., Andersson, L., Dobney, K., & Larson, G. (2014). Establishing the validity of domestication genes using DNA from ancient chickens. *Proceedings of the National Academy of Sciences* , *111* (17), 6184–6189. https://doi.org/10.1073/pnas.1308939110
KF753253	E1	E01	Girdland Flink, L., Allen, R., Barnett, R., Malmström, H., Peters, J., Eriksson, J., Andersson, L., Dobney, K., & Larson, G. (2014). Establishing the validity of domestication genes using DNA from ancient chickens. *Proceedings of the National Academy of Sciences* , *111* (17), 6184–6189. https://doi.org/10.1073/pnas.1308939110
KF753254	E1	E01	Girdland Flink, L., Allen, R., Barnett, R., Malmström, H., Peters, J., Eriksson, J., Andersson, L., Dobney, K., & Larson, G. (2014). Establishing the validity of domestication genes using DNA from ancient chickens. *Proceedings of the National Academy of Sciences* , *111* (17), 6184–6189. https://doi.org/10.1073/pnas.1308939110
KF753255	E1	E01	Girdland Flink, L., Allen, R., Barnett, R., Malmström, H., Peters, J., Eriksson, J., Andersson, L., Dobney, K., & Larson, G. (2014). Establishing the validity of domestication genes using DNA from ancient chickens. *Proceedings of the National Academy of Sciences* , *111* (17), 6184–6189. https://doi.org/10.1073/pnas.1308939110
KF753256	E1	E01	Girdland Flink, L., Allen, R., Barnett, R., Malmström, H., Peters, J., Eriksson, J., Andersson, L., Dobney, K., & Larson, G. (2014). Establishing the validity of domestication genes using DNA from ancient chickens. *Proceedings of the National Academy of Sciences* , *111* (17), 6184–6189. https://doi.org/10.1073/pnas.1308939110
KF753257	E1	E01	Girdland Flink, L., Allen, R., Barnett, R., Malmström, H., Peters, J., Eriksson, J., Andersson, L., Dobney, K., & Larson, G. (2014). Establishing the validity of domestication genes using DNA from ancient chickens. *Proceedings of the National Academy of Sciences* , *111* (17), 6184–6189. https://doi.org/10.1073/pnas.1308939110
KF753258	E1	E01	Girdland Flink, L., Allen, R., Barnett, R., Malmström, H., Peters, J., Eriksson, J., Andersson, L., Dobney, K., & Larson, G. (2014). Establishing the validity of domestication genes using DNA from ancient chickens. *Proceedings of the National Academy of Sciences* , *111* (17), 6184–6189. https://doi.org/10.1073/pnas.1308939110
KF753259	E1	E01	Girdland Flink, L., Allen, R., Barnett, R., Malmström, H., Peters, J., Eriksson, J., Andersson, L., Dobney, K., & Larson, G. (2014). Establishing the validity of domestication genes using DNA from ancient chickens. *Proceedings of the National Academy of Sciences* , *111* (17), 6184–6189. https://doi.org/10.1073/pnas.1308939110
KF753260	E1	E01	Girdland Flink, L., Allen, R., Barnett, R., Malmström, H., Peters, J., Eriksson, J., Andersson, L., Dobney, K., & Larson, G. (2014). Establishing the validity of domestication genes using DNA from ancient chickens. *Proceedings of the National Academy of Sciences* , *111* (17), 6184–6189. https://doi.org/10.1073/pnas.1308939110
KF753261	E1	E01	Girdland Flink, L., Allen, R., Barnett, R., Malmström, H., Peters, J., Eriksson, J., Andersson, L., Dobney, K., & Larson, G. (2014). Establishing the validity of domestication genes using DNA from ancient chickens. *Proceedings of the National Academy of Sciences* , *111* (17), 6184–6189. https://doi.org/10.1073/pnas.1308939110
KF753262	E1	E01	Girdland Flink, L., Allen, R., Barnett, R., Malmström, H., Peters, J., Eriksson, J., Andersson, L., Dobney, K., & Larson, G. (2014). Establishing the validity of domestication genes using DNA from ancient chickens. *Proceedings of the National Academy of Sciences* , *111* (17), 6184–6189. https://doi.org/10.1073/pnas.1308939110
KF753263	E1	E01	Girdland Flink, L., Allen, R., Barnett, R., Malmström, H., Peters, J., Eriksson, J., Andersson, L., Dobney, K., & Larson, G. (2014). Establishing the validity of domestication genes using DNA from ancient chickens. *Proceedings of the National Academy of Sciences* , *111* (17), 6184–6189. https://doi.org/10.1073/pnas.1308939110
KF753264	E1	E01	Girdland Flink, L., Allen, R., Barnett, R., Malmström, H., Peters, J., Eriksson, J., Andersson, L., Dobney, K., & Larson, G. (2014). Establishing the validity of domestication genes using DNA from ancient chickens. *Proceedings of the National Academy of Sciences* , *111* (17), 6184–6189. https://doi.org/10.1073/pnas.1308939110
KF753265	E1	E01	Girdland Flink, L., Allen, R., Barnett, R., Malmström, H., Peters, J., Eriksson, J., Andersson, L., Dobney, K., & Larson, G. (2014). Establishing the validity of domestication genes using DNA from ancient chickens. *Proceedings of the National Academy of Sciences* , *111* (17), 6184–6189. https://doi.org/10.1073/pnas.1308939110
KF753266	E1	E01	Girdland Flink, L., Allen, R., Barnett, R., Malmström, H., Peters, J., Eriksson, J., Andersson, L., Dobney, K., & Larson, G. (2014). Establishing the validity of domestication genes using DNA from ancient chickens. *Proceedings of the National Academy of Sciences* , *111* (17), 6184–6189. https://doi.org/10.1073/pnas.1308939110
KF753267	E1	E01	Girdland Flink, L., Allen, R., Barnett, R., Malmström, H., Peters, J., Eriksson, J., Andersson, L., Dobney, K., & Larson, G. (2014). Establishing the validity of domestication genes using DNA from ancient chickens. *Proceedings of the National Academy of Sciences* , *111* (17), 6184–6189. https://doi.org/10.1073/pnas.1308939110
KF753268	E1	E01	Girdland Flink, L., Allen, R., Barnett, R., Malmström, H., Peters, J., Eriksson, J., Andersson, L., Dobney, K., & Larson, G. (2014). Establishing the validity of domestication genes using DNA from ancient chickens. *Proceedings of the National Academy of Sciences* , *111* (17), 6184–6189. https://doi.org/10.1073/pnas.1308939110
KF753269	E1	E01	Girdland Flink, L., Allen, R., Barnett, R., Malmström, H., Peters, J., Eriksson, J., Andersson, L., Dobney, K., & Larson, G. (2014). Establishing the validity of domestication genes using DNA from ancient chickens. *Proceedings of the National Academy of Sciences* , *111* (17), 6184–6189. https://doi.org/10.1073/pnas.1308939110
KF753270	E1	E01	Girdland Flink, L., Allen, R., Barnett, R., Malmström, H., Peters, J., Eriksson, J., Andersson, L., Dobney, K., & Larson, G. (2014). Establishing the validity of domestication genes using DNA from ancient chickens. *Proceedings of the National Academy of Sciences* , *111* (17), 6184–6189. https://doi.org/10.1073/pnas.1308939110
KF753271	E1	E01	Girdland Flink, L., Allen, R., Barnett, R., Malmström, H., Peters, J., Eriksson, J., Andersson, L., Dobney, K., & Larson, G. (2014). Establishing the validity of domestication genes using DNA from ancient chickens. *Proceedings of the National Academy of Sciences* , *111* (17), 6184–6189. https://doi.org/10.1073/pnas.1308939110
KF753272	E1	E01	Girdland Flink, L., Allen, R., Barnett, R., Malmström, H., Peters, J., Eriksson, J., Andersson, L., Dobney, K., & Larson, G. (2014). Establishing the validity of domestication genes using DNA from ancient chickens. *Proceedings of the National Academy of Sciences* , *111* (17), 6184–6189. https://doi.org/10.1073/pnas.1308939110
KF753273	E1	E01	Girdland Flink, L., Allen, R., Barnett, R., Malmström, H., Peters, J., Eriksson, J., Andersson, L., Dobney, K., & Larson, G. (2014). Establishing the validity of domestication genes using DNA from ancient chickens. *Proceedings of the National Academy of Sciences* , *111* (17), 6184–6189. https://doi.org/10.1073/pnas.1308939110
KF753274	E1	E01	Girdland Flink, L., Allen, R., Barnett, R., Malmström, H., Peters, J., Eriksson, J., Andersson, L., Dobney, K., & Larson, G. (2014). Establishing the validity of domestication genes using DNA from ancient chickens. *Proceedings of the National Academy of Sciences* , *111* (17), 6184–6189. https://doi.org/10.1073/pnas.1308939110
KF753275	E1	E01	Girdland Flink, L., Allen, R., Barnett, R., Malmström, H., Peters, J., Eriksson, J., Andersson, L., Dobney, K., & Larson, G. (2014). Establishing the validity of domestication genes using DNA from ancient chickens. *Proceedings of the National Academy of Sciences* , *111* (17), 6184–6189. https://doi.org/10.1073/pnas.1308939110
KF753276	E1	E01	Girdland Flink, L., Allen, R., Barnett, R., Malmström, H., Peters, J., Eriksson, J., Andersson, L., Dobney, K., & Larson, G. (2014). Establishing the validity of domestication genes using DNA from ancient chickens. *Proceedings of the National Academy of Sciences* , *111* (17), 6184–6189. https://doi.org/10.1073/pnas.1308939110
KF753277	E1	E01	Girdland Flink, L., Allen, R., Barnett, R., Malmström, H., Peters, J., Eriksson, J., Andersson, L., Dobney, K., & Larson, G. (2014). Establishing the validity of domestication genes using DNA from ancient chickens. *Proceedings of the National Academy of Sciences* , *111* (17), 6184–6189. https://doi.org/10.1073/pnas.1308939110
KF753278	E1	E01	Girdland Flink, L., Allen, R., Barnett, R., Malmström, H., Peters, J., Eriksson, J., Andersson, L., Dobney, K., & Larson, G. (2014). Establishing the validity of domestication genes using DNA from ancient chickens. *Proceedings of the National Academy of Sciences* , *111* (17), 6184–6189. https://doi.org/10.1073/pnas.1308939110

KF753279	E1	E01	Girdland Flink, L., Allen, R., Barnett, R., Malmström, H., Peters, J., Eriksson, J., Andersson, L., Dobney, K., & Larson, G. (2014). Establishing the validity of domestication genes using DNA from ancient chickens. *Proceedings of the National Academy of Sciences* , *111* (17), 6184–6189. https://doi.org/10.1073/pnas.1308939110
KF753280	E1	E01	Girdland Flink, L., Allen, R., Barnett, R., Malmström, H., Peters, J., Eriksson, J., Andersson, L., Dobney, K., & Larson, G. (2014). Establishing the validity of domestication genes using DNA from ancient chickens. *Proceedings of the National Academy of Sciences* , *111* (17), 6184–6189. https://doi.org/10.1073/pnas.1308939110
KF753281	E1	E01	Girdland Flink, L., Allen, R., Barnett, R., Malmström, H., Peters, J., Eriksson, J., Andersson, L., Dobney, K., & Larson, G. (2014). Establishing the validity of domestication genes using DNA from ancient chickens. *Proceedings of the National Academy of Sciences* , *111* (17), 6184–6189. https://doi.org/10.1073/pnas.1308939110
KF753282	E1	E01	Girdland Flink, L., Allen, R., Barnett, R., Malmström, H., Peters, J., Eriksson, J., Andersson, L., Dobney, K., & Larson, G. (2014). Establishing the validity of domestication genes using DNA from ancient chickens. *Proceedings of the National Academy of Sciences* , *111* (17), 6184–6189. https://doi.org/10.1073/pnas.1308939110
KF753283	E1	E01	Girdland Flink, L., Allen, R., Barnett, R., Malmström, H., Peters, J., Eriksson, J., Andersson, L., Dobney, K., & Larson, G. (2014). Establishing the validity of domestication genes using DNA from ancient chickens. *Proceedings of the National Academy of Sciences* , *111* (17), 6184–6189. https://doi.org/10.1073/pnas.1308939110
KF753284	E1	E01	Girdland Flink, L., Allen, R., Barnett, R., Malmström, H., Peters, J., Eriksson, J., Andersson, L., Dobney, K., & Larson, G. (2014). Establishing the validity of domestication genes using DNA from ancient chickens. *Proceedings of the National Academy of Sciences* , *111* (17), 6184–6189. https://doi.org/10.1073/pnas.1308939110
KF753285	E1	E01	Girdland Flink, L., Allen, R., Barnett, R., Malmström, H., Peters, J., Eriksson, J., Andersson, L., Dobney, K., & Larson, G. (2014). Establishing the validity of domestication genes using DNA from ancient chickens. *Proceedings of the National Academy of Sciences* , *111* (17), 6184–6189. https://doi.org/10.1073/pnas.1308939110
KF753286	E1	E01	Girdland Flink, L., Allen, R., Barnett, R., Malmström, H., Peters, J., Eriksson, J., Andersson, L., Dobney, K., & Larson, G. (2014). Establishing the validity of domestication genes using DNA from ancient chickens. *Proceedings of the National Academy of Sciences* , *111* (17), 6184–6189. https://doi.org/10.1073/pnas.1308939110
KF753287	E1	E137*	Girdland Flink, L., Allen, R., Barnett, R., Malmström, H., Peters, J., Eriksson, J., Andersson, L., Dobney, K., & Larson, G. (2014). Establishing the validity of domestication genes using DNA from ancient chickens. *Proceedings of the National Academy of Sciences* , *111* (17), 6184–6189. https://doi.org/10.1073/pnas.1308939110
KF753288	E1	E137*	Girdland Flink, L., Allen, R., Barnett, R., Malmström, H., Peters, J., Eriksson, J., Andersson, L., Dobney, K., & Larson, G. (2014). Establishing the validity of domestication genes using DNA from ancient chickens. *Proceedings of the National Academy of Sciences* , *111* (17), 6184–6189. https://doi.org/10.1073/pnas.1308939110
KF753289	E1	E140*	Girdland Flink, L., Allen, R., Barnett, R., Malmström, H., Peters, J., Eriksson, J., Andersson, L., Dobney, K., & Larson, G. (2014). Establishing the validity of domestication genes using DNA from ancient chickens. *Proceedings of the National Academy of Sciences* , *111* (17), 6184–6189. https://doi.org/10.1073/pnas.1308939110
KP307147	E1	E138*	Dyomin, A. G., Danilova, M. I., Mwacharo, J. M., Masharsky, A. E., Panteleev, A. V., Druzhkova, A. S., Trifonov, V. A., & Galkina, S. A. (2017). Mitochondrial DNA D-loop haplogroup contributions to the genetic diversity of East European domestic chickens from Russia. Journal of Animal Breeding and Genetics, 134(2), 98–108. https://doi.org/10.1111/jbg.12248
KP307148	E1	E139*	Dyomin, A. G., Danilova, M. I., Mwacharo, J. M., Masharsky, A. E., Panteleev, A. V., Druzhkova, A. S., Trifonov, V. A., & Galkina, S. A. (2017). Mitochondrial DNA D-loop haplogroup contributions to the genetic diversity of East European domestic chickens from Russia. Journal of Animal Breeding and Genetics, 134(2), 98–108. https://doi.org/10.1111/jbg.12248
KP307149	C1	C01	Dyomin, A. G., Danilova, M. I., Mwacharo, J. M., Masharsky, A. E., Panteleev, A. V., Druzhkova, A. S., Trifonov, V. A., & Galkina, S. A. (2017). Mitochondrial DNA D-loop haplogroup contributions to the genetic diversity of East European domestic chickens from Russia. Journal of Animal Breeding and Genetics, 134(2), 98–108. https://doi.org/10.1111/jbg.12248
KP307150	E1	E137*	Dyomin, A. G., Danilova, M. I., Mwacharo, J. M., Masharsky, A. E., Panteleev, A. V., Druzhkova, A. S., Trifonov, V. A., & Galkina, S. A. (2017). Mitochondrial DNA D-loop haplogroup contributions to the genetic diversity of East European domestic chickens from Russia. Journal of Animal Breeding and Genetics, 134(2), 98–108. https://doi.org/10.1111/jbg.12248
KU258196	E1	E137*	Dyomin, A. G., Danilova, M. I., Mwacharo, J. M., Masharsky, A. E., Panteleev, A. V., Druzhkova, A. S., Trifonov, V. A., & Galkina, S. A. (2017). Mitochondrial DNA D-loop haplogroup contributions to the genetic diversity of East European domestic chickens from Russia. Journal of Animal Breeding and Genetics, 134(2), 98–108. https://doi.org/10.1111/jbg.12248

* new haplotype name given in this study
